# Supplementary material for: Effects of different aerobic exercises on the global cognitive function of the elderly with mild cognitive impairment: a meta-analysis
Source: BMJ Open. 2023 Jun 30;13(6):e067293. doi: 10.1136/bmjopen-2022-067293 (PMC10314475; doi:10.1136/bmjopen-2022-067293)
Supplement: Supplementary data [file bmjopen-2022-067293supp001.pdf]

Embase Search strategy

Search number	Search Details	Results
1#	'aerobic exercise'/exp	19335
2#	'aerobic dance':ab,ti OR 'aerobic dancing':ab,ti OR aerobics:ab,ti OR 'aerobics exercise':ab,ti OR 'dancing, aerobic':ab,ti OR 'exercise, aerobic':ab,ti OR 'low impact aerobic exercise':ab,ti OR 'low impact aerobics':ab,ti OR 'step aerobics':ab,ti	1495
3#	'aerobic exercise'/exp OR ('aerobic dance':ab,ti OR 'aerobic dancing':ab,ti OR aerobics:ab,ti OR 'aerobics exercise':ab,ti OR 'dancing, aerobic':ab,ti OR 'exercise, aerobic':ab,ti OR 'low impact aerobic exercise':ab,ti OR 'low impact aerobics':ab,ti OR 'step aerobics':ab,ti)	20255
4#	'exercise'/exp	399161
5#	'biometric exercise':ab,ti OR effort:ab,ti OR 'exercise capacity':ab,ti OR 'exercise performance':ab,ti OR 'exercise training':ab,ti OR exertion:ab,ti OR 'fitness training':ab,ti OR 'fitness workout':ab,ti OR 'physical conditioning':ab,ti OR human:ab,ti OR 'physical effort':ab,ti OR 'physical exercise':ab,ti OR 'physical exertion':ab,ti OR 'physical work-out':ab,ti OR 'physical workout':ab,ti	3576566
6#	'exercise'/exp OR ('biometric exercise':ab,ti OR effort:ab,ti OR 'exercise capacity':ab,ti OR 'exercise performance':ab,ti OR 'exercise training':ab,ti OR exertion:ab,ti OR 'fitness training':ab,ti OR 'fitness workout':ab,ti OR 'physical conditioning':ab,ti OR human:ab,ti OR 'physical effort':ab,ti OR 'physical exercise':ab,ti OR 'physical exertion':ab,ti OR 'physical work-out':ab,ti OR 'physical workout':ab,ti)	3884161
7#	'physical activity'/exp	485896
8#	'activity, physical':ab,ti	1355
9#	'physical activity'/exp OR 'activity, physical':ab,ti	486507
10#	'cognitive defect'/exp	557350
11#	'cognition disorders':ab,ti OR 'cognitive defects':ab,ti OR 'cognitive deficit':ab,ti OR 'cognitive disability':ab,ti OR 'cognitive disorder':ab,ti OR 'cognitive disorders':ab,ti OR 'cognitive dysfunction':ab,ti OR 'cognitive impairment':ab,ti OR 'delirium, dementia, amnestic, cognitive disorders':ab,ti OR overinclusion:ab,ti OR 'response interference':ab,ti	136389
12#	'cognitive defect'/exp OR ('cognition disorders':ab,ti OR 'cognitive defects':ab,ti OR 'cognitive deficit':ab,ti OR 'cognitive disability':ab,ti OR 'cognitive	577692

	disorder':ab,ti OR 'cognitive disorders':ab,ti OR 'cognitive dysfunction':ab,ti OR 'cognitive impairment':ab,ti OR 'delirium, dementia, amnestic, cognitive disorders':ab,ti OR overinclusion:ab,ti OR 'response interference':ab,ti)	
13#	'mild cognitive impairment'/exp	32022
14#	'amnestic mild cognitive impairment':ab,ti	3430
15	'mild cognitive impairment'/exp OR 'amnestic mild cognitive impairment':ab,ti	32561
16#	('aerobic exercise'/exp OR ('aerobic dance':ab,ti OR 'aerobic dancing':ab,ti OR aerobics:ab,ti OR 'aerobics exercise':ab,ti OR 'dancing, aerobic':ab,ti OR 'exercise, aerobic':ab,ti OR 'low impact aerobic exercise':ab,ti OR 'low impact aerobics':ab,ti OR 'step aerobics':ab,ti)) OR ('exercise'/exp OR ('biometric exercise':ab,ti OR effort:ab,ti OR 'exercise capacity':ab,ti OR 'exercise performance':ab,ti OR 'exercise training':ab,ti OR exertion:ab,ti OR 'fitness training':ab,ti OR 'fitness workout':ab,ti OR 'physical conditioning':ab,ti OR human:ab,ti OR 'physical effort':ab,ti OR 'physical exercise':ab,ti OR 'physical exertion':ab,ti OR 'physical work-out':ab,ti OR 'physical workout':ab,ti)) OR ('physical activity'/exp OR 'activity, physical':ab,ti)	4258734
17#	('cognitive defect'/exp OR ('cognition disorders':ab,ti OR 'cognitive defects':ab,ti OR 'cognitive deficit':ab,ti OR 'cognitive disability':ab,ti OR 'cognitive disorder':ab,ti OR 'cognitive disorders':ab,ti OR 'cognitive dysfunction':ab,ti OR 'cognitive impairment':ab,ti OR 'delirium, dementia, amnestic, cognitive disorders':ab,ti OR overinclusion:ab,ti OR 'response interference':ab,ti)) OR ('mild cognitive impairment'/exp OR 'amnestic mild cognitive impairment':ab,ti)	577692
18#	(('aerobic exercise'/exp OR ('aerobic dance':ab,ti OR 'aerobic dancing':ab,ti OR aerobics:ab,ti OR 'aerobics exercise':ab,ti OR 'dancing, aerobic':ab,ti OR 'exercise, aerobic':ab,ti OR 'low impact aerobic exercise':ab,ti OR 'low impact aerobics':ab,ti OR 'step aerobics':ab,ti)) OR ('exercise'/exp OR ('biometric exercise':ab,ti OR effort:ab,ti OR 'exercise capacity':ab,ti OR 'exercise performance':ab,ti OR 'exercise training':ab,ti OR exertion:ab,ti OR 'fitness training':ab,ti OR 'fitness workout':ab,ti OR 'physical conditioning':ab,ti OR human:ab,ti OR 'physical effort':ab,ti OR 'physical exercise':ab,ti OR 'physical exertion':ab,ti OR 'physical work-out':ab,ti OR 'physical workout':ab,ti)) OR ('physical activity'/exp OR 'activity, physical':ab,ti)) AND (('cognitive defect'/exp OR ('cognition disorders':ab,ti OR 'cognitive defects':ab,ti OR 'cognitive deficit':ab,ti OR 'cognitive disability':ab,ti OR 'cognitive disorder':ab,ti OR 'cognitive disorders':ab,ti OR 'cognitive	76351

	dysfunction':ab,ti OR 'cognitive impairment':ab,ti OR 'delirium, dementia, amnestic, cognitive disorders':ab,ti OR overinclusion:ab,ti OR 'response interference':ab,ti)) OR ('mild cognitive impairment'/exp OR 'amnestic mild cognitive impairment':ab,ti))	
19#	'aged'/exp	3469661
20#	'aged patient':ab,ti OR 'aged people':ab,ti OR 'aged person':ab,ti OR 'aged subject':ab,ti OR elderly:ab,ti OR 'elderly patient':ab,ti OR 'elderly people':ab,ti OR 'elderly person':ab,ti OR 'elderly subject':ab,ti OR 'senior citizen':ab,ti OR senium:ab,ti	385314
21#	'aged'/exp OR ('aged patient':ab,ti OR 'aged people':ab,ti OR 'aged person':ab,ti OR 'aged subject':ab,ti OR elderly:ab,ti OR 'elderly patient':ab,ti OR 'elderly people':ab,ti OR 'elderly person':ab,ti OR 'elderly subject':ab,ti OR 'senior citizen':ab,ti OR senium:ab,ti)	3555832
22#	((('aerobic exercise'/exp OR ('aerobic dance':ab,ti OR 'aerobic dancing':ab,ti OR aerobics:ab,ti OR 'aerobics exercise':ab,ti OR 'dancing, aerobic':ab,ti OR 'exercise, aerobic':ab,ti OR 'low impact aerobic exercise':ab,ti OR 'low impact aerobics':ab,ti OR 'step aerobics':ab,ti)) OR ('exercise'/exp OR ('biometric exercise':ab,ti OR effort:ab,ti OR 'exercise capacity':ab,ti OR 'exercise performance':ab,ti OR 'exercise training':ab,ti OR exertion:ab,ti OR 'fitness training':ab,ti OR 'fitness workout':ab,ti OR 'physical conditioning':ab,ti OR human:ab,ti OR 'physical effort':ab,ti OR 'physical exercise':ab,ti OR 'physical exertion':ab,ti OR 'physical work-out':ab,ti OR 'physical workout':ab,ti)) OR ('physical activity'/exp OR 'activity, physical':ab,ti)) AND (('cognitive defect'/exp OR ('cognition disorders':ab,ti OR 'cognitive defects':ab,ti OR 'cognitive deficit':ab,ti OR 'cognitive disability':ab,ti OR 'cognitive disorder':ab,ti OR 'cognitive disorders':ab,ti OR 'cognitive dysfunction':ab,ti OR 'cognitive impairment':ab,ti OR 'delirium, dementia, amnestic, cognitive disorders':ab,ti OR overinclusion:ab,ti OR 'response interference':ab,ti)) OR ('mild cognitive impairment'/exp OR 'amnestic mild cognitive impairment':ab,ti))) AND ('aged'/exp OR ('aged patient':ab,ti OR 'aged people':ab,ti OR 'aged person':ab,ti OR 'aged subject':ab,ti OR elderly:ab,ti OR 'elderly patient':ab,ti OR 'elderly people':ab,ti OR 'elderly person':ab,ti OR 'elderly subject':ab,ti OR 'senior citizen':ab,ti OR senium:ab,ti))	16100

Pubmed Search strategy

Search number	Search Details	Results
1#	"Cognitive Dysfunction"[Mesh]	228,279
2#	(((((((((((((Exercises[Title/Abstract]) OR (Physical Activity[Title/Abstract])) OR (Physical Activities[Title/Abstract])) OR (Activities, Physical[Title/Abstract])) OR (Activity, Physical[Title/Abstract])) OR (Exercise*, Physical[Title/Abstract])) OR (Physical Exercise*[Title/Abstract])) OR (Acute Exercise*[Title/Abstract])) OR (Exercise*, Acute[Title/Abstract])) OR (Exercise*, Isometric[Title/Abstract])) OR (Isometric Exercise*[Title/Abstract])) OR (Exercise*, Aerobic[Title/Abstract])) OR (Aerobic Exercise*[Title/Abstract])) OR (Exercise Training*[Title/Abstract])) OR (Training*, Exercise[Title/Abstract])	293,650
3#	("Exercise"[Mesh]) OR ((((((((((((((Exercises[Title/Abstract]) OR (Physical Activity[Title/Abstract])) OR (Physical Activities[Title/Abstract])) OR (Activities, Physical[Title/Abstract])) OR (Activity, Physical[Title/Abstract])) OR (Exercise*, Physical[Title/Abstract])) OR (Physical Exercise*[Title/Abstract])) OR (Acute Exercise*[Title/Abstract])) OR (Exercise*, Acute[Title/Abstract])) OR (Exercise*, Isometric[Title/Abstract])) OR (Isometric Exercise*[Title/Abstract])) OR (Exercise*, Aerobic[Title/Abstract])) OR (Aerobic Exercise*[Title/Abstract])) OR (Exercise Training*[Title/Abstract])) OR (Training*, Exercise[Title/Abstract]))	409,704
4#	"Cognitive Dysfunction"[Mesh]	29,147
5#	((((((((((((Cognitive Dysfunctions[Title/Abstract]) OR (Dysfunction*, Cognitive[Title/Abstract])) OR (Cognitive Impairment*[Title/Abstract])) OR (Impairment*, Cognitive[Title/Abstract])) OR (Mild Cognitive Impairment*[Title/Abstract])) OR (Cognitive Impairment*, Mild[Title/Abstract])) OR (Impairment*, Mild Cognitive[Title/Abstract])) OR (Mild Neurocognitive Disorder*[Title/Abstract])) OR (Disorder*, Mild Neurocognitive[Title/Abstract])) OR (Neurocognitive Disorder*, Mild[Title/Abstract])) OR (Cognitive Decline*[Title/Abstract])) OR (Decline*, Cognitive[Title/Abstract])) OR (Mental Deterioration*[Title/Abstract])) OR (Deterioration*, Mental[Title/Abstract])	169,309
6#	("Cognitive Dysfunction"[Mesh]) OR (((((((((((((Cognitive Dysfunctions[Title/Abstract]) OR (Dysfunction*, Cognitive[Title/Abstract])) OR (Cognitive Impairment*[Title/Abstract])) OR (Impairment*, Cognitive[Title/Abstract])) OR (Mild Cognitive Impairment*[Title/Abstract])) OR (Cognitive Impairment*, Mild[Title/Abstract])) OR (Impairment*, Mild Cognitive[Title/Abstract])) OR (Mild Neurocognitive Disorder*[Title/Abstract])) OR (Disorder*, Mild Neurocognitive[Title/Abstract])) OR (Neurocognitive Disorder*, Mild[Title/Abstract])) OR (Cognitive Decline*[Title/Abstract])) OR (Decline*, Cognitive[Title/Abstract])) OR (Mental Deterioration*[Title/Abstract])) OR (Deterioration*, Mental[Title/Abstract]))	171,419
7#	(("Exercise"[Mesh]) OR ((((((((((((((Exercises[Title/Abstract]) OR (Physical Activity[Title/Abstract])) OR (Physical Activities[Title/Abstract])) OR (Activities, Physical[Title/Abstract])) OR (Activity, Physical[Title/Abstract])) OR (Exercise*, Physical[Title/Abstract])) OR (Physical	6,686

Exercise*[Title/Abstract])) OR (Acute Exercise*[Title/Abstract])) OR (Exercise*, Acute[Title/Abstract])) OR (Exercise*, Isometric[Title/Abstract])) OR (Isometric Exercise*[Title/Abstract])) OR (Exercise*, Aerobic[Title/Abstract])) OR (Aerobic Exercise*[Title/Abstract])) OR (Exercise Training*[Title/Abstract])) OR (Training*, Exercise[Title/Abstract]))) AND (("Cognitive Dysfunction"[Mesh]) OR (((((((((((((Cognitive Dysfunctions[Title/Abstract]) OR (Dysfunction*, Cognitive[Title/Abstract])) OR (Cognitive Impairment*[Title/Abstract])) OR (Impairment*, Cognitive[Title/Abstract])) OR (Mild Cognitive Impairment*[Title/Abstract])) OR (Cognitive Impairment*, Mild[Title/Abstract])) OR (Impairment*, Mild Cognitive[Title/Abstract])) OR (Mild Neurocognitive Disorder*[Title/Abstract])) OR (Disorder*, Mild Neurocognitive[Title/Abstract])) OR (Neurocognitive Disorder*, Mild[Title/Abstract])) OR (Cognitive Decline*[Title/Abstract])) OR (Decline*, Cognitive[Title/Abstract])) OR (Mental Deterioration*[Title/Abstract])) OR (Deterioration*, Mental[Title/Abstract])))

Cochrane Library Search strategy

Search number	Search Details	Results
1#	MeSH descriptor: [Exercise] explode all trees	32772
2#	(Exercise*, Acute):ti,ab,kw OR (Acute Exercise*):ti,ab,kw OR (Training*, Exercise):ti,ab,kw OR (Exercise Training*):ti,ab,kw OR (Exercise*, Isometric):ti,ab,kw (Word variations have been searched)	52015
3#	(Exercise*, Aerobic):ti,ab,kw OR (Aerobic Exercise*):ti,ab,kw OR (Physical Exercise*):ti,ab,kw OR (Physical Activity):ti,ab,kw OR (Activity, Physical):ti,ab,kw (Word variations have been searched)	97132
4#	(Activities, Physical):ti,ab,kw OR (Exercise*, Physical):ti,ab,kw OR (Physical Activitie):ti,ab,kw (Word variations have been searched)	68139
5#	#2 OR #3 OR #4	12098
6#	#1 OR #5	130117
7#	MeSH descriptor: [Cognitive Dysfunction] explode all trees	2809
8#	(Impairment*, Mild Cognitive):ti,ab,kw OR (Disorder*, Mild Neurocognitive):ti,ab,kw OR (Mild Neurocognitive Disorder*):ti,ab,kw OR (Mild Cognitive Impairment*):ti,ab,kw OR (Neurocognitive Disorder*, Mild):ti,ab,kw (Word variations have been searched)	5434
9#	(Cognitive Impairment*, Mild):ti,ab,kw OR (Dysfunction*, Cognitive):ti,ab,kw OR (Impairment*, Cognitive):ti,ab,kw OR (Cognitive Dysfunctions):ti,ab,kw OR (Cognitive Impairment*):ti,ab,kw (Word variations have been searched)	23085
10#	(Mental Deterioration*):ti,ab,kw OR (Decline*, Cognitive):ti,ab,kw OR (Deterioration*, Mental;):ti,ab,kw OR (Cognitive Decline*):ti,ab,kw (Word variations have been searched)	8024
11#	#7 OR #8 OR #9 OR #10	28106
12#	#6 AND #11	4714
13#	MeSH descriptor: [Aged] explode all trees	242328
14#	(Elderly):ti,ab,kw (Word variations have been searched)	57985
15#	#13 OR #14	283159
16#	#12 AND #15	1492
