# Supplementary material for: Effects of different aerobic exercises on the global cognitive function of the elderly with mild cognitive impairment: a meta-analysis
Source: BMJ Open. 2023 Jun 30;13(6):e067293. doi: 10.1136/bmjopen-2022-067293 (PMC10314475; doi:10.1136/bmjopen-2022-067293)
Supplement: Supplementary data [file bmjopen-2022-067293supp002.pdf]

Supplementary Table 1： Baseline characteristics of included trials

Aerobic exercise type	study	region		Participants			intervene				
				Sample size	age	female	manner	frequency	strength	MMSE	MoCA
multi-component aerobic exercise	Li, L (2021)[12]	Asia/ China	Experimental group	42	60–69（47.6%）、70–79（47.6%）、80 and above（4.8%）	64%	5 minutes warm-up, 5 minutes aerobic training, 5 minutes strength training, 5 minutes balance training, 5 minutes coordination training, 5 minutes sensitivity training	30 minutes × 5 times× 24 weeks (6 months).	The warm-up is a 3-4/10 Perceptual Nu (RPE) level, and the rest of the workout is 4–5/10 RPE	26.50 ± 1.33	21.52 ± 2.05
			Control group	42	60–69（42.9%）、70–79（50.0%）、80 and above（7.1%）	57%	General community health guidance			26.62 ± 1.46	21.14 ± 1.97
	Bademli (2019)[15]	Asian/ Turkey	Experimental group	30	72.24± 7.16	60%	10 minutes of warm-up, 20 minutes of rhythmic exercise (including 22 simple exercises), 10 minutes of cooling exercise (low-intensity exercise), 40 minutes of free walking.	80 minutes ×20 weeks, 4/3/7 days per week	3-6 metabolic equivalents (ME).	23.27 ± 2.17	
			Control group	30	70.67 ± 8.34	57%	Continue with your daily activities			23.42 ± 1.07	
	De Oliveira (2019)[14]	Euro-American/ Brazil	Experimental group	7	71.85 ± 5.69	85.71%	5 minutes of balance training,30 minutes of aerobic training, 20 minutes of strength training, 5 minutes of stretching exercises	60 minutes × 2 times × 12 weeks	The intensity of the main training is 70% of the maximum oxygen consumption (VO2max) or 80% of the predicted maximum heart rate (HRmax).	29 ± 0.61	
			Control group	12	78.20± 5.26	41.66%	Clinical follow-up without any physical training			27.8 ± 2.57	
	Langoni (2019)[13]	Euro-American/ Brazil	Experimental group	26	72.6 ±7.8	76.9%	Strength training takes about30 minutes (with equipment including ankle weight, resistance latex tape and dumbbells) and aerobic training (20-30 minutes of walking).	60 minutes× 2 times × 24 weeks	Aerobic training to walk at a maximum heart rate of 60% to 75%.	21.9 ± 4.8	
			Control group	26	71.9 ±7.9	76.9%	Do not initiate any form of physical or cognitive activity and keep in touch by phone			25.0 ± 4.7	
	Lam (2015)[16]	Asia/ China	Experimental group	147	75.5±6.7	76.9%	Stretching and conditioning exercises, mind-body exercises (such as tai chi) and aerobic exercises (such as static cycling).	120 minutes× 3×48 weeks (12 months).	Unknown	25.8 ± 2.3	

			Control group	131	75.4±6.1	77.9%	Social activities (e.g., tea parties, movie watching).			25.6 ± 2.4	
mind-body exercise	Li,F (2022)	Euro-America/ United States	Experimental group	22	74.5 ±5.6	36.4%	Traditional Tai Chi training	60 minutes× 2 times × 16 weeks	The metabolic equivalent of Tai Chi exercise is 3.5-4.3 MET		25.09 ± 2.43
			Control group	24	74.9±6.3	62.5%	Stretching exercises				25.13 ± 2.19
	Tao,J (2019)[10] ①	Asia/ China	Experimental group	20	66.17±4.17	75%	15 minutes to warm up, 40 minutes to eight danjin training and 5 minutes to cool down	60 minutes × 3 times × 24 weeks	The Batdan Nishiki Movement MET is 2.68		22.45 ± 2.16
			Control group	20	65.97±5.66	70%	Maintain their original level of physical activity, do not receive any specific exercise interventions, health education				21.00 ± 2.36
	Qi,M (2019)[18]	Asia/ China	Experimental group	16	70.6±6.2	69%	35 minutes of moderate intensity aerobic dance (5 minutes of warm-up, 25 minutes of target heart rate dance and 5 minutes of music cooling).	35 minutes × 3 times× 12 weeks (three months).	60-80% maximum heart rate as the target heart rate for aerobic dance	27.3 ± 1.3	22.6 ± 2.1
			Control group	16	69.1±8.1	75%	Routine care			27.1 ± 1.2	22.9 ± 1.7
	Zhu,Y (2018)[19]	Asia/ China	Experimental group	29	70.3±6.7	51.7%	5 minutes of warm-up, 25 minutes of target heart rate dance and 5 minutes of cooling	35 minutes × 3 times× 12 weeks (three months	The target heart rate is set at 60%-80% of your maximum heart rate		23.2 ± 1.9
			Control group	31	69.0±7.3	67.7%	Routine care				22.9 ± 2.1
	Lazarou, (2017)[20]	Euro-American/ Greece	Experimental group	66	65.89± 10.76	80%	International Ballroom Dance (5 minutes of warm-up, review of previous dance lessons, 45 minutes of new material (tricks/dances), and 10 minutes of cooling time).	60 minutes× 2 times× 40 weeks (10 months).	Dancing About 6 METs	27.60 ± 2.19	24.70 ± 2.25
			Control group	33	67.92±9.47	76%	No intervention was accepted, the educational meeting was received			26.88 ± 2.1	23.81 ± 3.1
	Doi,T (2017)[21]	Asia/ Japan	Experimental group	67	75.7±4.1	50.7%	The dance program is based on ballroom dances, including salsa, rumba, waltz, cha-cha, bruce, kitba and tango	60 minutes × once × 40 weeks	Dancing About 6 METs	26.0 ± 2.6	
			Control group	67	76.0±4.9	46.3%	Health education class			25.8 ± 2.4	
	Lam (2014)[22]	Asia/ China	Experimental group	171	77.2±6.3	73%	24 Styles of Tai Chi " Training.	During the introductory phase, the coaching process is 4 to 6 weeks.	The metabolic equivalent of Tai Chi exercise is 3.5-4.3 MET	25.1 ± 3.0	

								During the maintenance phase, the frequency of interventions was ≥ 30 minutes per day and ≥ 3 days a week. Until the 1stmonth of the 2nd month			
			Control group	218	78.3±6.6	79%	Stretching and relaxation exercises			24.4 ± 2.9	
Conventional aerobic exercise	Tomoto, (2021)[23]	Euro-American /USA	Experimental group	22	64.8 ± 6.4	55%	Treadmill running	1-10 weeks: 25-30 minutes × 3 times.<br>11-25 weeks: 30-35minutes× 3 or 4 times.26 weeks - 1 year: 30-40 minutes× 4-5 times	The intensity is 75%-85% of the maximum heart rate (HR).	29.0 ± 1.5	
			Control group	30	66. 1 ± 6.8	53%	Stretching and conditioning interventions			28.8 ± 1.4	
	Song,D (2019)[24]	Asia/ China	Experimental group	60	76.22 ±5.76	80%	Aerobic tread exercises	60 minutes× 3 times × 16 weeks	For moderate-intensity workouts using the Borg Scale, the Borg score is 12-14		22.03 ± 1.81
			Control group	60	75.33 ±6.78	70%	Health education class				22.10 ± 1.92
	Tao,J (2019)[10] ②	Asia/ China	Experimental group	17	64.32±2.60	59%	Warm up for 15 minutes, walk fast for 40 minutes, cool down for 5 minutes.	60 minutes × 3 times × 24 weeks	The participants' heart rate was controlled at 55%-75% of the reserve heart rate		21.47 ± 2.27
			Control group	20	65.97±5.6	70%	Maintain the original level of physical activity and do not receive any specific exercise interventions, health education				21.00 ± 2.36
	Choi,W (2018)[25]	Asia/ South Korea	Experimental group	30	74.90±5.10	80%	10min warm-up activity, 40min GKP exercise and 10min cooling activity.	60 minutes× 2 times × 6 weeks	Unknown		21.66 ± 3.24
			Control group	30	74.23±4.38	83%	Stretching, warm-up and cooling exercises are the same as in the exercise group				20.76 ± 3.02
	Kohanpour (2017)[26]	Asia/ Iran	Experimental group	10	60-70	0%	8min warm-up, (8-26)min running and cooling down for 5min	21-39 minutes × 3 times × 12 weeks	The intensity is 75% to 85% of the maximum heart rate	22.70 ± 1.63	
			Control group	10	60-70	0%	Habitual lifestyle			24.30 ± 0.94	
	Wei, X H (2014)[27]	Asia/ China	Experimental group	30	66.73±5.48	30%	Handball training	30 minutes × 5 times × 6 months (120 times).	The average intensity of aerobic exercise is about 60% of your maximum heart rate	24.33 ± 1.65	
			Control group	30	65.27±4.63	36.7%	Maintain the original way of life and entertainment			25.00 ± 1.29	

Varela, S (2012)[11] ①	Euro-American/ Spain	Experimental group	17	79.24± 10.07	Unknown	5 minutes of warm-up, 20 minutes of recumbent bike ride, and 5 minutes of stretching to cool down	30 minutes × 3 times × 12 weeks	Exercise intensity is set at 40% of the participant's reserve heart rate (maximum heart rate - resting heart rate).	19.86 ± 5.12
		Control group	15	79.40 ±6.72		Social entertainment			21.80 ± 3.23
Varela, S (2012)[11] ②	Euro-American/ Spain	Experimental group	16	76.44 ±11.38	Unknown	5 minutes of warm-up, 20 minutes of recumbent bike ride, and 5 minutes of stretching to cool down	30 minutes × 3 times × 12 weeks	The exercise intensity is set at 60% of the participant's reserve heart rate (maximum heart rate - resting heart rate).	20.81 ± 4.69
		Control group	15	79.40 ±6.72		Social entertainment			21.80 ± 3.23
