# Supplementary material for: Effects of different aerobic exercises on the global cognitive function of the elderly with mild cognitive impairment: a meta-analysis
Source: BMJ Open. 2023 Jun 30;13(6):e067293. doi: 10.1136/bmjopen-2022-067293 (PMC10314475; doi:10.1136/bmjopen-2022-067293)
Supplement: Supplementary data [file bmjopen-2022-067293supp003.pdf]

	Deviation risk rating	Number of studies	Numberof participants	MD	95% CI	I²	Q-test	P
Multi-component	Low	0	−	−	−	−	−	−
exercise (MMSE)	Moderate	1	52	4.60	2.20,7.00	−	−	−
	High	4	441	1.72	1.33,2.11	96%	<0. 00001	<0. 00001
mind-body	Low	0	−	−	−	−	−	−
exercise (MMSE)	Moderate	2	383	1.03	0.43,1.64	0%	0.57	0.0008
	High	2	161	1.61	0.91,2.30	76%	0.04	<0. 00001
conventional	Low	0	−	−	−	−	−	−
aerobic exercise	Moderate	5	195	0.51	0.09,0.93	0%	0.46	0.02
(MMSE)	High	0	−	−	−	−	−	−
Multi-component	Low	0	−	−	−	−	−	−
exercise(MoCA)	Moderate	0	−	−	−	−	−	−
	High	1	84	5.74	5.02,6.46	−	−	<0. 00001
mind-body	Low	1	58	1.10	0.06,2.14	−	−	0.04
exercise (MoCA)	Moderate	1	40	2.45	0.92,3.98	−	−	0.002
	High	2	78	1.04	0.17,1.91	6%	0.46	0.02
conventional	Low	1	120	2.26	1.51,3.01	−	−	<0. 00001
aerobic	Moderate	1	37	0.25	-1.04,1.54	−	−	0.70
exercise(MoCA)	High	1	60	3.67	2.18,5.16	−	−	<0. 00001
