# Supplementary material for: Effects of different aerobic exercises on the global cognitive function of the elderly with mild cognitive impairment: a meta-analysis
Source: BMJ Open. 2023 Jun 30;13(6):e067293. doi: 10.1136/bmjopen-2022-067293 (PMC10314475; doi:10.1136/bmjopen-2022-067293)
Supplement: Supplementary data [file bmjopen-2022-067293supp004.pdf]

The subgroup analysis of Multi-component exercise (MMSE)

Subgroups		Number of studies	Numberof participants	MD	95% CI	I^2	Q-test	P^g
Sample size [a]	Small	2	71	1.69*	0. 30，3. 08	88%	＜0.01	=0. 88
	Large	3	422	1.80**	1. 40，2. 20	97%	＜0. 01	
region	Asia	3	422	1.80**	1. 40，2. 20	97%	＜0. 01	=0. 88
	Europe	2	71	1.69*	0. 30，3. 08	88%	＜0. 01	
Exercise intensity [b]	low	1	84	2.37**	1. 59，3. 15	—	—	=0.01
	Medium to high	3	131	3.68**	3. 00，4. 36	88%	＜0. 01	
Duration of intervention [c]	short	1	84	2.37**	1. 59，3. 15	—	—	＜0. 01
	middle	3	349	0.33	-0. 20，0. 87	84%	＜0. 01	
	long	1	60	4.30**	3. 53，5. 08	—	—	
Frequency of intervention [d]	middle	3	349	0.33	-0. 20，0. 87	84%	＜0. 01	＜0. 01
	high	2	144	3.34**	2. 79，3. 88	92%	＜0. 01	
Total intervention time [e]	middle	4	409	1.61**	1. 17，2. 05	96%	＜0. 01	=0.09
	long	1	84	2.37**	1. 59，3. 15	—	—	
Deviation risk rating [f]	middle	1	52	4.60**	2.20，7.00	—	—	＜0. 01
	high	4	441	1.72**	1.33， 2.11	96%	＜0. 01	

[a]＜60=Small sample size；≥60=Large sample size，[b]Maximum heart rate <60% or Borg scale (10th grade) <5 or Borg scale (20th grade) < 12 or metabolic equivalent (MET) <3 or reserve heart rate <59% =Low strength；Maximum heart rate ≥ 60% or Borg scale (10th grade) ≥5 or Borg scale (20th grade) ≥ 12 or metabolic equivalent (MET) ≥3 or reserve heart rate ≥59% = medium to high intensity，[c]≤30 minutes = short；＞30 且≤60 minutes = middle； >60

minutes =long [d] 1 time/week = low; 2-3 times / week = middle； 4 times and more /week =high [e] ＜3 months (12 weeks) = short； ≥ 3 months and < 6 months (12-23 weeks) = middle； ≥6 months (24 weeks) =long [f] All criteria are rated as low risk= Low risk of bias；Some standards are rated as unclear and not rated as high risk = medium deviation risk; As long as a standard is rated as high risk = high risk of deviation, [g] Differences between groups

[*] P＜0.05

[**] P＜0.01
