# Supplementary material for: Effects of different aerobic exercises on the global cognitive function of the elderly with mild cognitive impairment: a meta-analysis
Source: BMJ Open. 2023 Jun 30;13(6):e067293. doi: 10.1136/bmjopen-2022-067293 (PMC10314475; doi:10.1136/bmjopen-2022-067293)
Supplement: Supplementary data [file bmjopen-2022-067293supp005.pdf]

Quality of the evidence(GRADE)

Quality assessment							No of patients		Effect		Quality	Importance
No of studies	Design	Risk of bias	Inconsistency	Indirectness	Imprecision	Other considerations	Aerobic exercise group	Control group	Relative (95% CI)	Absolute		
**Subgroup analysis of different Exercise Types (MMSE) - multi-component aerobic exercise VS controls (Better indicated by lower values)**												
1	randomised trials	very serious[1]	very serious[2]	no serious indirectness	no serious imprecision	none	257	236	-	MD 1.79 higher (1.41 to 2.17 higher)	⊕OOO VERY LOW	
**Subgroup analysis of different Exercise Types (MMSE) - mind-body exercise VS controls (Better indicated by lower values)**												
2	randomised trials	serious[3]	no serious inconsistency	no serious indirectness	no serious imprecision	none	233	311	-	MD 1.28 higher (0.83 to 1.74 higher)	⊕⊕⊕O MODERATE	
**Subgroup analysis of different Exercise Types (MMSE) - Conventional aerobic exercise VS controls (Better indicated by lower values)**												
3	randomised trials	serious[3]	no serious inconsistency	no serious indirectness	serious[4]	none	95	100	-	MD 0.51 higher (0.09 to 0.93 higher)	⊕⊕OO LOW	

[1] Downgraded two levels due to most studies are high risk.

[2] Downgraded two levels due to $I^2 > 75\%$.

[3] Downgraded one level due to most studies are of low and moderate risk.

[4] Downgraded one level due to the samle size is small.

Quality assessment							No of patients		Effect		Quality	Importance
No of studies	Design	Risk of bias	Inconsistency	Indirectness	Imprecision	Other considerations	Aerobic exercise group	Control group	Relative (95% CI)	Absolute		
**Subgroup analysis of different Exercise Types (MoCA)- multi-component aerobic exercise VS controls (Better indicated by lower values)**												
1	randomised trials	very serious[1]	no serious inconsistency	no serious indirectness	very serious[2]	none	42	42	-	MD 5.74 higher (5.02 to 6.46 higher)	⊕OOO VERY LOW	
**Subgroup analysis of different Exercise Types (MoCA) - mind-body exercise VS controls (Better indicated by lower values)**												
2	randomised trials	serious[3]	no serious inconsistency	no serious indirectness	serious[4]	none	85	91	-	MD 1.29 higher (0.67 to 1.9 higher)	⊕⊕OO LOW	
**Subgroup analysis of different Exercise Types (MoCA) - Conventional aerobic exercise VS controls (Better indicated by lower values)**												
3	randomised trials	serious[3]	very serious[5]	no serious indirectness	serious[4]	none	107	110	-	MD 2.06 higher (1.46 to 2.65 higher)	⊕OOO VERY LOW	

[1] Downgraded two levels due to most studies are high risk.

[2] Downgraded two levels due to the sample size is too small.

[3] Downgraded one level due to most studies are of low and moderate risk.

[4] Downgraded one level due to the sample size is small.

[5] Downgraded two levels due to $I^2 > 75\%$.
