# Supplementary material for: Effects of different aerobic exercises on the global cognitive function of the elderly with mild cognitive impairment: a meta-analysis
Source: BMJ Open. 2023 Jun 30;13(6):e067293. doi: 10.1136/bmjopen-2022-067293 (PMC10314475; doi:10.1136/bmjopen-2022-067293)
Supplement: Supplementary data [file bmjopen-2022-067293supp006.pdf]

Total sensitivity analysis（MMSE）

Study omitted	Mean difference	p-value
Bademli K 2019	0.91 [0.65,1.16]	<0.00001
de Oliveira2019	1.25 [1.01,1.50]	<0.00001
Doi T 2017	1.26 [1.01,1.51]	<0.00001
Kohanpour 2017	1.30 [1.05,1.55]	<0.00001
Lam LC 2014	1.24[0.98,1.49]	<0.00001
Lam LCW 2015	1.47[1.21,1.74]	<0.00001
Langoni 2019	1.20[0.96,1.44]	<0.00001
Lazarou 2017	1.16[0.92,1.41]	<0.00001
Li L 2021	1.11[0.86,1.37]	<0.00001
Qi M 2019	1.25[1.01,1.50]	<0.00001
Tomoto T 2021	1.35[1.10,1.60]	<0.00001
Varela S 2012①	1.23[0.99,1.47]	<0.00001
Varela S 2012②	1.23[0.99,1.47]	<0.00001
Wei XH 2014	1.31[1.04,1.57]	<0.00001

Total sensitivity analysis(MoCA)

Study omitted	Mean difference	p-value
Choi W 2018	2.90[2.53,3.26]	<0.00001
Lazarou 2017	2.74[2.37,3.10]	<0.00001
Li F 2022	3.14[2.77,3.52]	<0.00001
Li L 2021	2.03[1.62,2.44]	<0.00001
Qi M 2019	3.09[2.72,3.46]	<0.00001
Song D 2019	3.14[2.73,3.54]	<0.00001
Tao J 2019①	2.97[2.60,3.33]	<0.00001
Tao J 2019②	3.16[2.79,3.53]	<0.00001
Zhu Y 2018	3.18[2.80,3.56]	<0.00001
Choi W 2018	2.90[2.53,3.26]	<0.00001

The sensitivity analysis of Multi-component motion (MMSE)

Study omitted	Mean difference	p-value
Bademli K 2019	0.99[0.55,1.43]	<0.0001
by Oliveira2019	1.88[1.48,2.27]	<0.00001
Lam LCW 2015	3.11[2.60,3.63]	<0.00001
2019 Langoni	1.72[1.33,2.11]	<0.00001
Li L 2021	1.61[1.17,2.05]	<0.00001

The sensitivity analysis of mind-body exercise（MMSE）

Study omitted	Mean difference	p-value
Doi T 2017	1.44 [0.91,1.97]	<0.00001
Lam LC 2014	1.31 [0.77,1.86]	<0.00001
Lazarou 2017	1.00 [0.48,1.51]	<0.00001
Qi M 2019	1.39[0.87,1.90]	<0.00001

The sensitivity analysis of mind-body exercise (MoCA)

Study omitted	Mean difference	p-value
Li F 2022	1.29(0.55,2.03)	0. 0007
Qi M 2019	1.43(0.76,2.11)	<0.0001
Tao J 2019①	1.06(0.40,1.73)	0. 002
Zhu Y 2018	1.38(0.63,2.14)	0. 0003

The sensitivity analysis of conventional aerobic exercise (MMSE）

Study omitted	Mean difference	p-value
Kohanpour 2017	0.59(0.12,1.06)	0. 01
Tomoto T 2021	0.70(0.21,1.19)	0.005
Varela S 2012①	0.51(0.08,0.93)	0. 02
Varela S 2012②	0.50(0.07,0.92)	0. 02
Wei XH 2014	0.14(-0.47,0.75)	0. 65

The sensitivity analysis of conventional aerobic exercise (MoCA).

Study omitted	Mean difference	p-value
Choi W 2018	1.75(1.10,2.40)	<0.0001
Song D 2019	1.71(0.74,2.69)	<0.00001
Tao J 2019②	2.55(1.87,3.22)	<0.00001
