# Supplementary material for: Effects of different aerobic exercises on the global cognitive function of the elderly with mild cognitive impairment: a meta-analysis
Source: BMJ Open. 2023 Jun 30;13(6):e067293. doi: 10.1136/bmjopen-2022-067293 (PMC10314475; doi:10.1136/bmjopen-2022-067293)
Supplement: Author's manuscript [file bmjopen-2022-067293.draft_revisions.pdf]

BMJ Open

BMJ Open is committed to open peer review. As part of this commitment we make the peer review history of every article we publish publicly available.

When an article is published we post the peer reviewers' comments and the authors' responses online. We also post the versions of the paper that were used during peer review. These are the versions that the peer review comments apply to.

The versions of the paper that follow are the versions that were submitted during the peer review process. They are not the versions of record or the final published versions. They should not be cited or distributed as the published version of this manuscript.

BMJ Open is an open access journal and the full, final, typeset and author-corrected version of record of the manuscript is available on our site with no access controls, subscription charges or pay-per-view fees (http://bmjopen.bmj.com).

If you have any questions on BMJ Open's open peer review process please email info.bmjopen@bmj.com

BMJ Open

# Effects of different aerobic exercises on the global cognitive function of the elderly with mild cognitive impairment: a meta-analysis



| | |
|---|---|
| Journal: | *BMJ Open* |
| Manuscript ID | bmjopen-2022-067293 |
| Article Type: | Original research |
| Date Submitted by the Author: | 11-Aug-2022 |





| | |
|---|---|
| Complete List of Authors: | Han, Conglin; Beijing Rehabilitation Hospital, ; Weifang Medical University,<br>Sun, Weishuang; Weifang Medical University<br>Zhang, Dan; Weifang Medical University<br>Xi, Xiaoshuang; Capital Medical University<br>Zhang, Rong; Yunnan University of Chinese Medicine<br>Gong, Weijun; Capital Medical University |





| | |
|---|---|
| Keywords: | NEUROLOGY, Old age psychiatry < PSYCHIATRY, SPORTS MEDICINE |

SCHOLARONE™ Manuscripts

*I, the Submitting Author has the right to grant and does grant on behalf of all authors of the Work (as defined in the below author licence), an exclusive licence and/or a non-exclusive licence for contributions from authors who are: i) UK Crown employees; ii) where BMJ has agreed a CC-BY licence shall apply, and/or iii) in accordance with the terms applicable for US Federal Government officers or employees acting as part of their official duties; on a worldwide, perpetual, irrevocable, royalty-free basis to BMJ Publishing Group Ltd ("BMJ") its licensees and where the relevant Journal is co-owned by BMJ to the co-owners of the Journal, to publish the Work in this journal and any other BMJ products and to exploit all rights, as set out in our licence.*

*The Submitting Author accepts and understands that any supply made under these terms is made by BMJ to the Submitting Author unless you are acting as an employee on behalf of your employer or a postgraduate student of an affiliated institution which is paying any applicable article publishing charge ("APC") for Open Access articles. Where the Submitting Author wishes to make the Work available on an Open Access basis (and intends to pay the relevant APC), the terms of reuse of such Open Access shall be governed by a Creative Commons licence – details of these licences and which Creative Commons licence will apply to this Work are set out in our licence referred to above.*

*Other than as permitted in any relevant BMJ Author's Self Archiving Policies, I confirm this Work has not been accepted for publication elsewhere, is not being considered for publication elsewhere and does not duplicate material already published. I confirm all authors consent to publication of this Work and authorise the granting of this licence.*

For peer review only

# Effects of different aerobic exercises on the global cognitive function of the elderly with mild cognitive impairment: a meta-analysis

Conglin Han[1], Weishuang Sun[1], Dan Zhang[1], Xiaoshuang Xi[2], Rong Zhang[3], Weijun Gong[4*]

1 Beijing Rehabilitation Hospital, Capital Medical University, Beijing 100144, China
Rehabilitation Medicine Academy, Weifang Medical University, Shandong 261053, China
2 Beijing Rehabilitation Medicine Academy, Capital Medical University, Beijing 100144, China
3 Second Clinical Medical Academy, Yunnan University of Chinese Medicine, Yunnan 650500, China
4 Department of Neurological Rehabilitation, Beijing Rehabilitation Hospital, Capital Medical University, Beijing 100144, China

*Corresponding author：Weijun Gong, Correspondence: E-mail: gwj197104@ccmu.edu.cn.

ORCID：0000-0002-9134-8218 (Weijun Gong)

**Abstract**

OBJECTIVE: To summarize the effects of various types of aerobic exercise on the global cognitive function of the elderly with mild cognitive impairment(MCI).

METHODS: Electronic databases of PubMed, EMBASE, and the Cochrane Library, clinical trial registry websites, and clinical randomized controlled trials published in peer-reviewed journals were searched through March 2022. For included studies, differences in endpoint values for global cognitive function were analyzed across studies based on the outcome of the Simple Mental State Examination Scale (MMSE) or the Montreal Cognitive Assessment Scale (MoCA). A paired meta-analysis using a random effects model was performed to directly test the efficacy of different types of exercise.

RESULTS: A total of 1680 patients who participated in 20 randomized controlled trials were included in this study. Based on outcomes of MMSE analysis, the aerobic exercise, which were beneficial for global cognitive function in MCI patients, included multi-component aerobic exercise (MD=1.79, 95% CI [1.41, 2.17], $p<0.01$), mind-body exercise (MD=1.29, 95% CI [0.67, 1.90], $p<0.01$) and conventional aerobic exercise MD=0.51, 95% CI [0.09, 0.93 ], $p<0.05$). With evaluation of MoCA, multi-component aerobic exercise (MD=5.54,95% CI [5.02, 6.46], $p<0.01$), mind-body exercise (MD=1.29,95% CI [0.67, 1.90], $p<0.01$) and conventional aerobic exercise (MD=2.06,95% CI [1.46, 2.65], $p< 0.01$) were showed significant beneficial effects for the patient. However, there was a high degree of heterogeneity between the results of multi-component aerobic exercise (MMSE) and conventional aerobic exercise (MoCA), which was analyzed and explored.

CONCLUSIONS: In general, multi-component aerobic exercise, mind-body exercise, and conventional aerobic exercise were beneficial in improving global cognitive function in the elderly with mild cognitive impairment. However, there was high heterogeneity in the results of multi-component aerobic and conventional aerobic exercise, whereas the improvement effect of mind-body exercise was more reliable.

Keywords: Mild cognitive impairment, aerobic exercise, elderly

For peer review only

**Strengths and limitations of this study**

This is a comprehensive meta-analysis of aerobic exercise interventions for mild cognitive impairment (MCI), with a particular focus on the different types of aerobic exercise types individually.

By analyzing the results of overall cognitive function, we concluded that all types of aerobic exercise were beneficial for the cognitive function of MCI patients.

This meta-analysis explores and highlights the heterogeneity of some of the results and important limitations in the literature, such as older and female MCI patients, who may have poorer outcomes for aerobic exercise interventiofns.

## 1.Introduction

Along the development and improvement in modern science, medical technology and treatment, the average life expectancy of people around the world has been continuously extended. Age-related health problems are becoming more and more prominent. Dementia is one of the common diseases of aging and a major cause of disability in the elderly[1]. The World Health Organization estimates that about 50 million people worldwide suffer from dementia, with a new case every three seconds, and the number of people with dementia will triple by 2050[2]. Severe dementia can place a huge emotional and financial burden on families and society, so it is important to take measures for early detection, early intervention, and early treatment of dementia. Mild cognitive impairment (MCI) is an intermediate state between normal cognition and dementia. MCI usually refers to cognitive decline beyond that caused by normal aging, but without significant impairment in Activity of Daily Living (ADL)[3]. Currently, medications have a limited role in preventing and treating MCI[4,5], so there is a large body of research focusing on non-pharmacological interventions.

It has suggested that some non-pharmacological interventions, such as food, exercise, and cognitive stimulation, may be beneficial for the improvement of cognitive function in MCI patients[3,4,6]. It has also shown that physical activity has a greater influence on the cognitive performance of MCI patients than drugs. For the elderly population with decline in cognitive function , a number of studies have also confirmed that aerobic exercise appears to be an effective for improving cognitive function[7-9]. However, there are various types of aerobic exercise, and the aerobic exercise that can be performed may vary in different populations (For example, it is difficult for people with lower limb mobility restrictions to run). As a result, an analysis is needed to compare the effects of different types of aerobic exercise on the improvement of global cognitive function in the elderly with MCI.

## 2. Methods

This review has been registered in the International Prospective Systematic Evaluation Registry database (PROSPERO, http://www.cdr.york.ac.uk/PROSPERO/) under the registration number: CRD42022327386. We performed a meta-analysis according to the PRISMA guidelines.

### 2.1. Inclusion criteria

We included randomized controlled trials assessing the health improvement effects of aerobic exercise on adults with MCI. Participants had to be 60 years of age and older, without dementia, other mental health problems or neurological disorders (such as stroke, depression, Parkinson's). The intervention were various forms of exercise containing aerobic exercise, including conventional aerobic exercise (e.g., brisk walking, running, cycling), mind-body exercise (e.g., Tai Chi, dancing), and multi-component aerobic exercise (aerobic exercise combined with other forms of exercise, such as resistance training, balance training). Aerobic exercise was defined as the involvement of major muscle groups throughout the body in addition to the primary involvement of oxygen for energy supply. Controls could be exercises of stretching, activities of health education, routine care, daily lifestyle and social recreation. The global cognitive function indicator was provided as a mean (M) and standard deviation (SD).

### 2.2. Exclusion criteria

Intervention methods could only be a variety of exercise training modalities; those with specific instructions to have other training components, such as cognitive training, were excluded. Non-intervention studies, articles with only research protocols, review articles, unpublished studies, abstracts or papers, and articles for which full text or data were not available were excluded. Non-English articles were excluded.

### 2.3. Search strategy

We systematically searched the PubMed, EMBASE, and Cochrane Library electronic databases, clinical trial registration websites, and clinical randomized controlled trials published in peer-reviewed journals from the earliest available record until March 2022. We searched using subject terms and free words. The keywords used included search terms related to sports exercise (e.g., exercise or physical activity or aerobic exercise) and cognitive impairment (e.g., mild cognitive impairment or cognitive dysfunction or amnestic mild cognitive impairment), and search terms related to older adults ( e.g., older adults or older patients). The search strategy for Embase is presented in Supplementary note. Endnote X9.1 was used to store and sort the retrieved randomized controlled trials and to remove duplicates. Initial screening was performed by the first author based on the title and abstract. This was followed by an independent screening of the full text by two reviewers according to the eligibility criteria specified in the study protocol. Disagreements in included studies were resolved by negotiation.

### 2.4. Data extraction and analysis

A standardized data extraction form was applied to extract data based on the following parameters: (1) basic information about the included studies (author, year, country) (2) basic characteristics of the study population (sample size, age, and gender of participants) (3) intervention characteristics (type of intervention, the intervention specifics, frequency and intensity of the intervention) (4) Outcome measures: we chose the two most commonly used scales for assessing global cognitive functioning, the Mini-Mental Status Examination (MMSE) and the Montreal Cognitive Assessment (MoCA). We performed meta-analysis using the endpoint measures.



For peer review only - http://bmjopen.bmj.com/site/about/guidelines.xhtml

For peer review only

Meta-analysis was performed using RevMan 5.3 software, and measures were analyzed using mean difference (MD) as the effect analysis statistic and providing its 95% CI. The heterogeneity between the results of the included studies was analyzed using the $\chi^2$ test (test level α=0.1), while the magnitude of heterogeneity was determined quantitatively in combination with $I^2$. Heterogeneity between trial design options was unclear; therefore, a random-effects model was chosen. Significant clinical heterogeneity was addressed using subgroup analysis or sensitivity analysis, or only descriptive analysis was performed. Sensitivity analyses were performed by sequentially excluding individual studies and then re-running the Meta-analysis and assessing the differences between the excluded results and the original combined results.

**2.5. Literature quality evaluation**

The Cochrane risk of bias assessment tool was used to independently assess the risk of bias in included trials. The assessment covered the following parameters: random sequence generation, allocation concealment, blinding, incomplete data, selective reporting, and others. Each component was divided into three categories: low risk, unclear, and high risk. Each study was categorized using the following criteria: low risk of bias (all criteria were rated as low risk); moderate risk of bias (there were criteria rated as unclear and not rated as high risk); and (whenever there were criteria rated as high risk). Two reviewers assessed each trial independently.

**2.6. Patient and public involvement**

There was no direct patient or public involvement in this review.

**3. Results**

**3.1. Search results**

By searching electronic databases and literature, we obtained a total of 24,255 relevant references. After duplicates were removed, and titles and abstracts were checked against predefined inclusion criteria, 102 complete references were retained, and we reviewed each paper in detail. A total of 84 trials did not meet the predefined inclusion criteria: non-randomized controlled trials (n=4), no control group meeting the criteria (n=5), full text not available (n=6), assessment results not meeting the criteria (n=23), the study without results (n=9), data not available (n=13), intervention modality not meeting the pre-defined criteria (n=7), and study population not meeting the inclusion criteria (n=17). Therefore, a total of 18 articles were included in this study. The search process is shown in Figure 1.

**3.2 Research characteristics**

A total of 18 randomized controlled trials were included in this study, published from 2011 to 2022. The characteristics of all included studies are summarized in Supplementary table 1. The studies were conducted in different countries: China (n=8), the United States (n=2), Brazil (n=2), Japan (n=1), Korea (n=1), Turkey (n=1), Iran (n=1), Greece (n=1), and Spain (n=1). All included participants were 1700 and they were assigned to either the experimental or control group, and the sample size of the study ranged from 19-389. All participants were diagnosed with MCI. One study[10] was composed of the mind-body exercise intervention group and the conventional aerobic intervention group with their corresponding the control group. Another study[11] was designed as two groups with different exercise intensities (40% and 60% reserve heart rate) for the conventional aerobic intervention. For all included studies, 5 experimental groups underwent multi-component exercise training with aerobic exercise[12-16]; 7 experimental groups underwent mind-body exercise training[10,17-22]; 8 experimental groups underwent conventional aerobic exercise training[10,11,23-27]. The total intervention duration for all studies ranged from 6 weeks to 12 months. The frequency was 21-80 min per session 1-5 times per week. Three experimental groups had an exercise intensity of roughly low intensity[10-12], i.e., maximum heart rate <60% or Borg scale (level 10) <5 or Borg scale (level 20) <12 or metabolic equivalent during exercise (MET) <3 or reserve heart rate <59%; 15 experimental groups had an exercise intensity of roughly moderate to high intensity[10,11,13-15,17-24,26,27], i.e., maximum heart rate ≥60% or Borg scale (10th grade) ≥5 or Borg scale (20th grade)) ≥ 12 or metabolic equivalent during exercise (MET) ≥ 3 or reserve heart rate ≥ 59%; the intensity of exercise in two other experimental groups was unclear[16,25].Ten studies assessed global cognitive function with the MMSE[11,13-16,21-23,26,27];5 studies assessed global cognitive function with the MoCA[10,17,19,24,25]; and 3 additional studies used both the MMSE and the MoCA[12,18,20].

**3.3. Literature quality evaluation**

As shown in Figure 2, the quality of studies included in the trials varied. Two trials had a low overall risk of bias[19,24], five trials had a moderate overall risk of bias[10,13,17,22,23], and eleven trials had a high overall risk of bias[11,12,14-16,18,20,21,25-27].

**3.4. Effects of aerobic exercises on global cognitive function**

We analyzed the trials in which the assessment results were MMSE and MoCA separately. The detailed analysis was then divided into multi-component aerobic exercise, mind-body exercise, and conventional aerobic exercise according to the exercise modality.

**3.4.1 The assessment with MMSE**

**3.4.1.1 The improvement of global cognitive function**

A total of 13 trials were included, and as shown in Figure 3, random-effects model meta-analysis revealed that aerobic exercise with MMSE outcomes was effective in improving global cognitive function compared to controls (MD=1.39, 95% CI [0.62, 2.17], p<0.01). Significant heterogeneity was found in the study ($I^2$=88%, p<0.01).

For peer review only



For peer review only - http://bmjopen.bmj.com/site/about/guidelines.xhtml

### 3.4.1.2 The effect of various forms of exercise on global cognitive function

All trials were divided into multi-component aerobic exercise, mind-body exercise, and conventional aerobic exercise by exercise modality, and the results of the meta-analysis was shown in Figure 4. As can be seen from the figure, after grouping by exercise modality, the difference between groups was significant ($I^2$=89.7%, p<0.01) indicating that the grouping was meaningful. Based on the results after grouping, it was clear that the conventional aerobic exercise intervention group had a significant improvement in global cognitive function (MD=0.51, 95% CI [0.09, 0.93], P<0.05) and a significant decrease in heterogeneity ($I^2$=0%, P=0.48); and the mind-body exercise intervention was also resulted in a significant improvement effect (MD=1.28, 95% CI [0.83 1.74], P<0.01) and a significant decrease in heterogeneity ($I^2$=50%, P=0.11); while multi-component aerobic exercise, despite also having a significant beneficial effect (MD=1.79, 95% CI [1.41, 2.17], P<0.01) had a very high heterogeneity ($I^2$=95%, P<0.01) and therefore needs further analysis. We performed sensitivity analyses and subgroup analyses on the multi-component aerobic exercise intervention group, and the results were shown in Tables 1 and 2. After sensitivity analyses, the removal of either study did not show any influence on the results obtained and heterogeneity remained almost unchanged. We performed subgroup analyses based on the sample size of the trial, exercise intensity, geographic region, risk assessment grading, duration, frequency, and total duration of the intervention. It was clear from the results that no meaningful results were obtained after subgroup analysis.

Table 1：The sensitivity analysis of Multi-component exercise (MMSE)

| Exclude studies | MD | 95% CI | $I^2$,% | Homogeneity Test | |
|---|---|---|---|---|---|
| | | | | Q | P |
| Bademli K 2019 | 0.99** | 0.55,1.43 | 90% | 30.76 | ＜0. 01 |
| de Oliveira2019 | 1.88)** | 1.48,2.27 | 96% | 80.32 | ＜0. 01 |
| Lam LCW 2015 | 3.11** | 2.60,3.63 | 88% | 25.06 | ＜0. 01 |
| 2019 Langoni | 1.72** | 1.33,2.11 | 96% | 78.37 | ＜0. 01 |
| Li L 2021 | 1.61** | 1.17,2.05 | 96% | 80.96 | ＜0. 01 |

** P < 0.01

Table 2：The subgroup analysis of Multi-component exercise (MMSE)

| Subgroups | | Number of studies | Numberof participants | MD | 95% CI | $I^2$ | Q-test | $P^g$ |
|---|---|---|---|---|---|---|---|---|
| Sample size [a] | Small | 2 | 71 | 1.69* | 0. 30，3. 08 | 88% | ＜0.01 | =0. 88 |
| | Large | 3 | 422 | 1.80** | 1. 40，2. 20 | 97% | ＜0. 01 | |
| region | Asia | 3 | 422 | 1.80** | 1. 40，2. 20 | 97% | ＜0. 01 | =0. 88 |
| | Europe | 2 | 71 | 1.69* | 0. 30，3. 08 | 88% | ＜0. 01 | |
| Exercise intensity [b] | low | 1 | 84 | 2.37** | 1. 59，3. 15 | — | — | =0.01 |
| | Medium to high | 3 | 131 | 3.68** | 3. 00，4. 36 | 88% | ＜0. 01 | |
| Duration of intervention [c] | short | 1 | 84 | 2.37** | 1. 59，3. 15 | — | — | ＜0. 01 |
| | middle | 3 | 349 | 0.33 | -0. 20，0. 87 | 84% | ＜0. 01 | |
| | long | 1 | 60 | 4.30** | 3. 53，5. 08 | — | — | |
| Frequency of intervention [d] | middle | 3 | 349 | 0.33 | -0. 20，0. 87 | 84% | ＜0. 01 | ＜0. 01 |
| | high | 2 | 144 | 3.34** | 2. 79，3. 88 | 92% | ＜0. 01 | |
| Total intervention time [e] | middle | 4 | 409 | 1.61** | 1. 17，2. 05 | 96% | ＜0. 01 | =0.09 |
| | long | 1 | 84 | 2.37** | 1. 59，3. 15 | — | — | |
| Deviation risk rating [f] | middle | 1 | 52 | 4.60** | 2.20，7.00 | — | — | ＜0. 01 |
| | high | 4 | 441 | 1.72** | 1.33， 2.11 | 96% | ＜0. 01 | |

[a]＜60=Small sample size；≥60=Large sample size，[b]Maximum heart rate <60% or Borg scale (10th grade) <5 or Borg scale (20th grade) < 12 or metabolic equivalent (MET) <3 or reserve heart rate <59% =Low strength； Maximum heart rate ≥ 60% or Borg scale (10th grade) ≥5 or Borg scale (20th grade) ≥ 12 or metabolic equivalent (MET) ≥3 or reserve heart rate ≥59% = medium to high intensity，[c]≤30 minutes = short； ＞30 且≤60 minutes = middle； >60 minutes =long [d] 1 time/week = low; 2-3 times / week = middle； 4 times and more /week =high [e]＜3 months (12 weeks) = short； ≥ 3 months and < 6 months (12-23 weeks) = middle； ≥6 months (24 weeks) =long [f]All criteria are rated as low risk= Low risk of bias；



For peer review only - http://bmjopen.bmj.com/site/about/guidelines.xhtml

For peer review only

Some standards are rated as unclear and not rated as high risk = medium deviation risk; As long as a standard is rated as high risk = high risk of deviation, [g]Differences between groups
* P＜0.05
** P＜0.01

### 3.4.2 The assessment with MoCA

#### 3.4.2.1 The improvement of global cognitive function

A total of 8 trials were included, and as shown in Figure 5, the random-effects model meta-analysis revealed that aerobic exercise with MoCA outcomes was effective in improving global cognitive function compared to controls (MD=2.62,95% CI [1.14, 4.10], $p<0.01$). Significant heterogeneity was found in the study ($I^2=94\%$, $p<0.01$).

#### 3.4.2.2 The effect of various forms of exercise on global cognitive function

Similarly, all trials with MoCA results were categorized by exercise modality into multi-component aerobic exercise, mind-body exercise, and conventional aerobic exercise. Meta-analysis results were shown in Figure 6. The significant difference between the groups with different exercise modality interventions ($I^2=97.6\%$, $p<0.01$) indicated that the effect of different exercise intervention groups was distinct. The cognitive function scores were significantly higher in the mind-body exercise intervention group than in the control group (MD=1.29,95% CI [0.67, 1.90], $p<0.01$) and less heterogeneous ($I^2=6\%$, $p=0.36$), which seemed to show that the improvement of mind-body exercise was better. Although the results were also beneficial in the conventional aerobic exercise intervention group (MD=2.06,95% CI [1.46, 2.65], $p<0.01$), the greater heterogeneity ($I^2=84\%$, $p<0.01$) still needed further explanation. There was only one multi-component aerobic exercise trial with a positive MoCA outcome (MD=5.54, 95% CI [5.02, 6.46], $p<0.01$). Since there were only three trials in the conventional aerobic exercise intervention group, it was not convenient to subdivide them into subgroups. After sensitivity analysis on a case-by-case basis, as shown in Table 3, we found a small decrease in heterogeneity after excluding the article Tao J 2019[10] ($I^2=63\%$, $p=0.10$) .

Table 3：The sensitivity analysis of routine aerobic exercise (MoCA).

| Exclude studies | MD | 95% CI | $I^2$,% | Homogeneity Test | |
|---|---|---|---|---|---|
| | | | | Q | P |
| Choi In 2018 | 1.75** | 1.10,2.40 | 86% | 6.96 | ＜0. 01 |
| Song D 2019 | 1.71** | 0.74,2.69 | 91% | 11.54 | ＜0. 01 |
| Tao J 2019② | 2.55** | 1.87,3.22 | 63% | 2.73 | =0. 10 |

** P < 0.01

## 4. Discussion

This meta-analysis explored the effects of different aerobic exercise types on global cognitive function in the elderly with MCI. The studies in this review were from different countries, including China. The overall quality of trials was variable, with most trials having a moderate or high risk of bias. Our study analyzed improvements in cognitive function through endpoint indicators of global cognitive function and found that all three types of aerobic exercise above produced positive outcomes. This review may provide valuable evidence for future studies.

### 4.1.Summary of the literature

There were few previous meta-analyses[28,29] that explored the effects of multi-component exercise on cognitive function in MCI. Although some of their results suggested a beneficial effect of multi-component aerobic exercise on cognitive function in the elderly with MCI, the intervention criteria for the studies included in these meta-analyses were not only exercise but also cognitive training and mental stimulation, which were not the same as in our study. The Meta-analysis by Borges -Machado et al. [30] discussed multi-component exercise, which was defined as exercise that combined aerobic, strength, posture, and balance training. Although the study found improvements in ADL in the subjects, the study was conducted in patients with dementia and the results did not determine the effects on cognitive function.

Previous studies[31,32] confirmed the beneficial effects of mind-body exercise on global cognitive function on people with MCI. Unlike the present study, they had slight differences in the type of trial studies, age, and outcome evaluation indicators in the inclusion criteria. Our inclusion criteria were relatively more stringent. These previous studies combined different cognitive evaluation metrics, expressed as standardized mean differences (SMD). We selected the two most common indicators of global cognitive function evaluation (MMSE and MoCA) to be analyzed separately and expressed the results as mean difference (MD).

We did not find studies on conventional aerobic exercise. Although there are a few reviews[9,33,34] reporting aerobic exercise in MCI patients, they did not analyze the types of aerobic exercise separately. Therefore, we believed that the analysis of the present study was still of practical relevance.

### 4.2. Findings of this study

The results of our study demonstrated a significant effect of multi-component aerobic exercise, mind-body exercise, and conventional aerobic exercise on global cognitive function in older adults with MCI analyzed by both the MMSE and MoCA. However, there was high heterogeneity in the results of both the MMSE meta-analysis of multi-component aerobic exercise and the MoCA meta-analysis of conventional aerobic exercise. We focused on these points.



For peer review only

For peer review only - http://bmjopen.bmj.com/site/about/guidelines.xhtml

**4.3. Analysis of the results of mind-body exercise**

All studies of mind-body exercise reported positive results. The results of both the MMSE and MoCA meta-analyses were significant effects and low heterogeneity, which would seem to indicate the effectiveness of mind-body exercise in improving cognitive function in older adults with MCI. One possible explanation for the beneficial results of mind-body exercise was because that mind-body exercise was a form of physical exercise supplemented with different degrees of cognitive function training. For example, Tai Chi, a Chinese system of slow meditative physical exercise designed for mind relaxation and balance, required attention, memory, and other aspects in addition to physical activity. Adding cognitive training to physical activity seems to had better effects for cognitive function improvement than training alone[35]. Another possible explanation was that depression was an important risk factor for cognitive decline in people with MCI and that mind-body exercise improved mood in older adults better than other physical exercises[31].

**4.4. Heterogeneity analysis**

From a total of five studies analyzed by MMSE for multi-component aerobic exercise, two studies[14,16] were no meaningful change in outcomes. We analyzed these studies and speculated that the possible reasons for such high heterogeneity. In the trial studied by de Oliveira et al.[14], the authors indicated that participants in both the trial and control groups were receiving medication, but no further medication-related descriptions. We are not sure if the medication affected the cognitive function of the subjects and thus the effect of the intervention. In another trial[16] that reported negative results, each exercise type of its multi-component aerobic exercise was performed separately, whereas common multi-component exercises combine different components in a single exercise session[30]. This was therefore the most different aspect of this study from other multi-component exercise training, and one possible explanation was that different components were more effective when combined in a single exercise session. One study showed[36] that short-term tai chi training did not significantly improve cognitive function in adults when comparing long-term and short-term tai chi training. In another trial[12], the duration of aerobic exercise was too short at only 5 minutes. Thus we speculated that there may be some difference in the results produced by different time of aerobic exercise.

Conventional aerobic exercise is more common than multi-component aerobic exercise and mind-body exercise. The meta-analysis of conventional aerobic exercise in MoCA had a high heterogeneity. It was well known that various physical conditions deteriorate with age in the elderly population, and the risk of progression to dementia in MCI patients increases accordingly[37]. Therefore, we speculated that the older the MCI patients were, the less beneficial the intervention would be. Some of the study[25] subjects were residents of the same community and were more female (80%), which may have made the study less representative. A review[38] had shown that older women have a higher degree of cognitive deterioration than men, which seemed to be possibly related to estrogen. In addition to these possible reasons, problems such as small sample sizes, poor subject compliance, and investigator implementation bias that occurred in some studies[10,14-16] may also have contributed to the high heterogeneity.

**4.5. Limitations**

There were several limitations of this review. One was that there was considerable heterogeneity between some of the included studies, representing both clinical and methodological heterogeneity, due to differences between participants, interventions, and study designs. The second was that it could not rule out that certain control groups would also be of varying degrees of benefit to cognitive function, such as social activities[39]. Third, only some of the trials that assessed global cognitive outcomes were included, and not those that assessed separately cognitive domains. Fourth, only studies of trials with MMSE and MoCA outcomes were selected. Although these two cognitive evaluation metrics are the most commonly used, they also undoubtedly excluded other studies that might be of value.

**5. Conclusion**

The results of this meta-analysis suggest that all types of aerobic exercise (including multi-component aerobic exercise, mind-body exercise, and conventional aerobic exercise) were positive for improving global cognitive function in older adults with MCI. However, the conclusion should be treated cautiously due to the heterogeneity and limited research. Additional well-designed RCTs are needed to provide more evidence for this conclusion.

**Ethics statements**

Patient consent for publication

Not applicable.

**Ethics approval**

Not applicable.

Figure 1: Study flow diagram.
Figure 2: Risk of bias assessment of included trials.
Figure 3: Effect sizes of forest plots for aerobic exercise versus controls-MMSE outcomes. MMSE, mini-mental state examination.
Figure 4: Effect sizes of forest plots for the different aerobic exercise-MMSE outcomes. MMSE, mini-mental state examination.
Figure 5: Effect sizes of forest plots for aerobic exercise versus controls-MoCA outcome. MoCA, Montreal Cognitive Assessment.
Figure 6: Effect sizes of forest plots for the different aerobic exercise-MoCA outcomes. MoCA, Montreal Cognitive Assessment



For peer review only - http://bmjopen.bmj.com/site/about/guidelines.xhtml

For peer review only

## References


[1] The Lancet Public H. Reinvigorating the public health response to dementia[J]. Lancet Public Health, 2021, 6(10): e696.

[2] WHO Guidelines Approved by the Guidelines Review Committee, Risk Reduction of Cognitive Decline and Dementia: WHO Guidelines, Geneva: World Health Organization © World Health Organization 2019., 2019.

[3] Sanford A M. Mild Cognitive Impairment[J]. Clin Geriatr Med, 2017, 33(3): 325-337.

[4] Jongsiriyanyong S, Limpawattana P. Mild Cognitive Impairment in Clinical Practice: A Review Article[J]. Am J Alzheimers Dis Other Demen, 2018, 33(8): 500-507.

[5] Schneider L S, Mangialasche F, Andreasen N, et al. Clinical trials and late-stage drug development for Alzheimer's disease: an appraisal from 1984 to 2014[J]. J Intern Med, 2014, 275(3): 251-83.

[6] Eshkoor S A, Hamid T A, Mun C Y, et al. Mild cognitive impairment and its management in older people[J]. Clin Interv Aging, 2015, 10: 687-93.

[7] Law C K, Lam F M, Chung R C, et al. Physical exercise attenuates cognitive decline and reduces behavioural problems in people with mild cognitive impairment and dementia: a systematic review[J]. J Physiother, 2020, 66(1): 9-18.

[8] Ahlskog J E, Geda Y E, Graff-Radford N R, et al. Physical exercise as a preventive or disease-modifying treatment of dementia and brain aging[J]. Mayo Clin Proc, 2011, 86(9): 876-84.

[9] Yong L, Liu L, Ding T, et al. Evidence of Effect of Aerobic Exercise on Cognitive Intervention in Older Adults With Mild Cognitive Impairment[J]. Front Psychiatry, 2021, 12: 713671.




For peer review only

For peer review only - http://bmjopen.bmj.com/site/about/guidelines.xhtml

[10] Tao J, Liu J, Chen X, et al. Mind-body exercise improves cognitive function and modulates the function and structure of the hippocampus and anterior cingulate cortex in patients with mild cognitive impairment[J]. Neuroimage. Clinical, 2019, 23: 101834.

[11] Varela S, Ayán C, Cancela J M, et al. Effects of two different intensities of aerobic exercise on elderly people with mild cognitive impairment: a randomized pilot study[J]. Clin Rehabil, 2012, 26(5): 442-50.

[12] Li L, Liu M, Zeng H, et al. Multi-component exercise training improves the physical and cognitive function of the elderly with mild cognitive impairment: a six-month randomized controlled trial[J]. Annals of palliative medicine, 2021, 10(8): 8919-8929.

[13] Langoni C D S, Resende T L, Barcellos A B, et al. Effect of Exercise on Cognition, Conditioning, Muscle Endurance, and Balance in Older Adults With Mild Cognitive Impairment: a Randomized Controlled Trial[J]. Journal of geriatric physical therapy (2001), 2019, 42(2): E15-E22.

[14] De Oliveira Silva F, Ferreira J V, Plácido J, et al. Three months of multimodal training contributes to mobility and executive function in elderly individuals with mild cognitive impairment, but not in those with Alzheimer's disease: a randomized controlled trial[J]. Maturitas, 2019, 126: 28-33.

[15] Bademli K, Lok N, Canbaz M, et al. Effects of Physical Activity Program on cognitive function and sleep quality in elderly with mild cognitive impairment: a randomized controlled trial[J]. Perspectives in psychiatric care, 2019, 55(3): 401-408.

[16] Lam L C, Chan W C, Leung T, et al. Would older adults with mild cognitive impairment adhere to and benefit from a structured lifestyle activity intervention to enhance cognition?: a

For peer review only

cluster randomized controlled trial[J]. PloS one, 2015, 10(3): e0118173.

[17] Li F, Harmer P, Fitzgerald K, et al. A cognitively enhanced online Tai Ji Quan training intervention for community-dwelling older adults with mild cognitive impairment: a feasibility trial[J]. BMC geriatrics, 2022, 22(1): 76.

[18] Qi M, Zhu Y I, Zhang L, et al. The effect of aerobic dance intervention on brain spontaneous activity in older adults with mild cognitive impairment: A resting-state functional MRI study[J]. Experimental and Therapeutic Medicine, 2019, 17(1): 715-722.

[19] Zhu Y, Wu H, Qi M, et al. Effects of a specially designed aerobic dance routine on mild cognitive impairment[J]. Clinical interventions in aging, 2018, 13: 1691-1700.

[20] Lazarou I, Parastatidis T, Tsolaki A, et al. International Ballroom Dancing Against Neurodegeneration: a Randomized Controlled Trial in Greek Community-Dwelling Elders With Mild Cognitive impairment[J]. American journal of Alzheimer's disease and other dementias, 2017, 32(8): 489-499.

[21] Doi T, Verghese J, Makizako H, et al. Effects of Cognitive Leisure Activity on Cognition in Mild Cognitive Impairment: Results of a Randomized Controlled Trial[J]. Journal of the American Medical Directors Association, 2017, 18(8): 686-691.

[22] Lam L C, Chan W M, Kwok T C, et al. Effectiveness of Tai Chi in maintenance of cognitive and functional abilities in mild cognitive impairment: a randomised controlled trial[J]. Hong Kong Med J, 2014, 20(3 Suppl 3): 20-3.

[23] Tomoto T, Liu J, Tseng B Y, et al. One-Year Aerobic Exercise Reduced Carotid Arterial Stiffness and Increased Cerebral Blood Flow in Amnestic Mild Cognitive Impairment[J]. Journal of Alzheimer's disease, 2021, 80(2): 841-853.



For peer review only - http://bmjopen.bmj.com/site/about/guidelines.xhtml

[24] Song D, Yu D S F. Effects of a moderate-intensity aerobic exercise programme on the cognitive function and quality of life of community-dwelling elderly people with mild cognitive impairment: a randomised controlled trial[J]. International journal of nursing studies, 2019, 93: 97-105.

[25] Choi W, Lee S. Ground Kayak Paddling Exercise Improves Postural Balance, Muscle Performance, and Cognitive Function in Older Adults with Mild Cognitive Impairment: a Randomized Controlled Trial[J]. Medical science monitor, 2018, 24: 3909-3915.

[26] Kohanpour M-A, Mohammad-Ali K, Maghsoud P, et al. The effects of aerobic exercise with lavender essence use on cognitive state and serum brain-derived neurotrophic factor levels in elderly with mild cognitive impairment[J]. Journal of herbmed pharmacology, 2017, 6(2): 80-84.

[27] Wei X H, Ji L L. Effect of handball training on cognitive ability in elderly with mild cognitive impairment[J]. Neuroscience letters, 2014, 566: 98-101.

[28] Özbe D, Graessel E, Donath C, et al. Immediate Intervention Effects of Standardized Multicomponent Group Interventions on People with Cognitive Impairment: A Systematic Review[J]. J Alzheimers Dis, 2019, 67(2): 653-670.

[29] Bruderer-Hofstetter M, Rausch-Osthoff A K, Meichtry A, et al. Effective multicomponent interventions in comparison to active control and no interventions on physical capacity, cognitive function and instrumental activities of daily living in elderly people with and without mild impaired cognition - A systematic review and network meta-analysis[J]. Ageing Res Rev, 2018, 45: 1-14.

[30] Borges-Machado F, Silva N, Farinatti P, et al. Effectiveness of Multicomponent Exercise Interventions in Older Adults With Dementia: A Meta-Analysis[J]. Gerontologist, 2021, 61(8):



For peer review only - http://bmjopen.bmj.com/site/about/guidelines.xhtml

e449-e462.

[31] Wang S, Yin H, Jia Y, et al. Effects of Mind-Body Exercise on Cognitive Function in Older Adults With Cognitive Impairment: A Systematic Review and Meta-analysis[J]. J Nerv Ment Dis, 2018, 206(12): 913-924.

[32] Zou L, Loprinzi P D, Yeung A S, et al. The Beneficial Effects of Mind-Body Exercises for People With Mild Cognitive Impairment: a Systematic Review With Meta-analysis[J]. Arch Phys Med Rehabil, 2019, 100(8): 1556-1573.

[33] Cammisuli D M, Innocenti A, Franzoni F, et al. Aerobic exercise effects upon cognition in Mild Cognitive Impairment: A systematic review of randomized controlled trials[J]. Arch Ital Biol, 2017, 155(1-2): 54-62.

[34] Zheng G, Xia R, Zhou W, et al. Aerobic exercise ameliorates cognitive function in older adults with mild cognitive impairment: a systematic review and meta-analysis of randomised controlled trials[J]. Br J Sports Med, 2016, 50(23): 1443-1450.

[35] Gheysen F, Poppe L, Desmet A, et al. Physical activity to improve cognition in older adults: can physical activity programs enriched with cognitive challenges enhance the effects? A systematic review and meta-analysis[J]. Int J Behav Nutr Phys Act, 2018, 15(1): 63.

[36] Walsh J N, Manor B, Hausdorff J, et al. Impact of Short- and Long-term Tai Chi Mind-Body Exercise Training on Cognitive Function in Healthy Adults: Results From a Hybrid Observational Study and Randomized Trial[J]. Glob Adv Health Med, 2015, 4(4): 38-48.

[37] Petersen R C, Lopez O, Armstrong M J, et al. Practice guideline update summary: Mild cognitive impairment: Report of the Guideline Development, Dissemination, and Implementation Subcommittee of the American Academy of Neurology[J]. Neurology, 2018,

For peer review only

90(3): 126-135.

[38] Alchalabi T, Prather C. Brain Health[J]. Clin Geriatr Med, 2021, 37(4): 593-604.

[39] James B D, Wilson R S, Barnes L L, et al. Late-life social activity and cognitive decline in old age[J]. J Int Neuropsychol Soc, 2011, 17(6): 998-1005.

For peer review only



For peer review only - http://bmjopen.bmj.com/site/about/guidelines.xhtml

**Contributors:** All authors made a substantial contribution to this work. Conglin Han, Weishuang Sun, Dan Zhang, Xiaoshuang Xi, Rong Zhang and Weijun Gong all contributed to the conception and design of the review. Conglin Han drafted the paper with all the authors critically reviewing it and suggesting amendments prior to submission. All authors had full access to all of the data (including statistical reports and tables) in the study and can take responsibility for the integrity of the data and the accuracy of the data analysis.
**Funding:** This work was supported by Capital's Funds for Health Improvement and Research(2022-1-2251), Natural Science Foundation of China (81972148) and Beijing Natural Science Foundation of China (7222101).
**Competing interests:** None declared.
**Provenance and peer review:** Not commissioned; externally peer reviewed.
**Data sharing statement:** Details of excluded papers are available from the first author on request.

I [Conglin Han], the Submitting Author has the right to grant and does grant on behalf of all authors of the Work (as defined in the below author licence), an exclusive licence and/or a non-exclusive licence for contributions from authors who are: i) UK Crown employees; ii) where BMJ has agreed a CC BY licence shall apply, and/or iii) in accordance with the terms applicable for US Federal Government officers or employees acting as part of their official duties; on a worldwide, perpetual, irrevocable, royalty-free basis to BMJ Publishing Group Ltd ("BMJ") its licensees and where the relevant Journal is co-owned by BMJ to the co-owners of the Journal, to publish the Work in [JOURNAL NAME] and any other BMJ products and to exploit all rights, as set out in our licence [link]



For peer review only - http://bmjopen.bmj.com/site/about/guidelines.xhtml

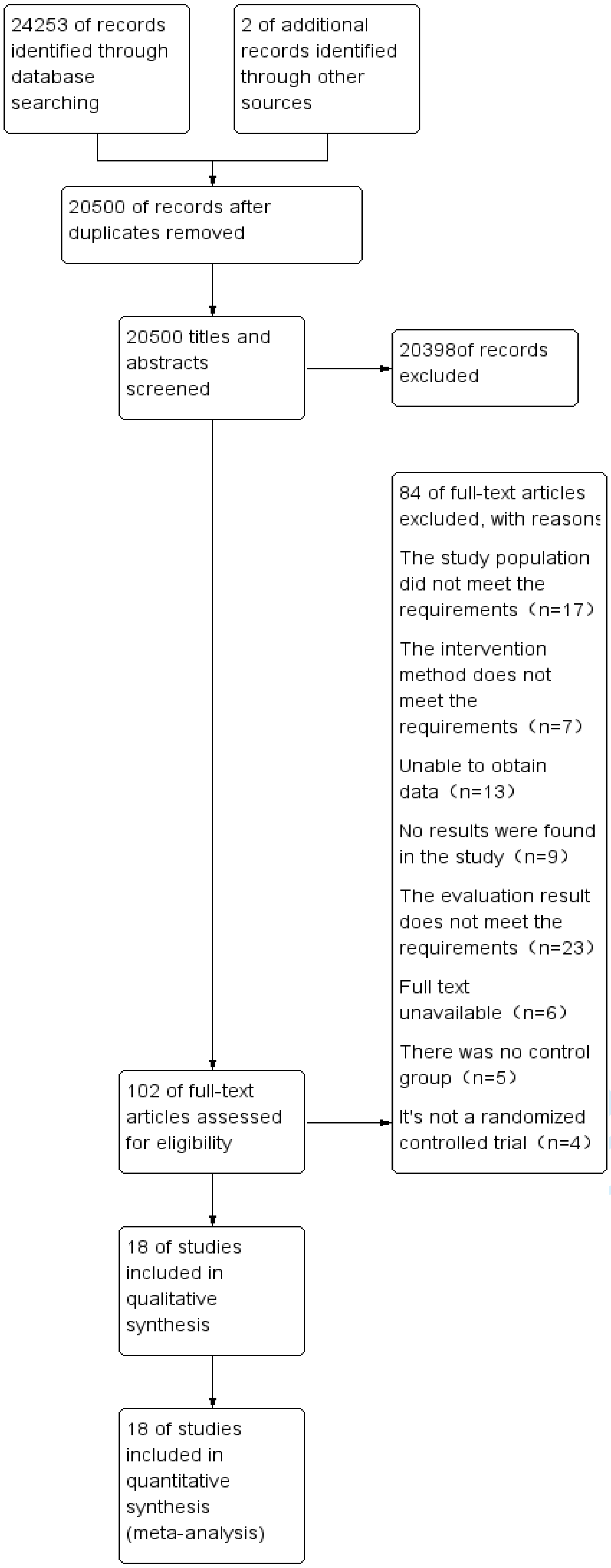


Figure 1： Study flow diagram.

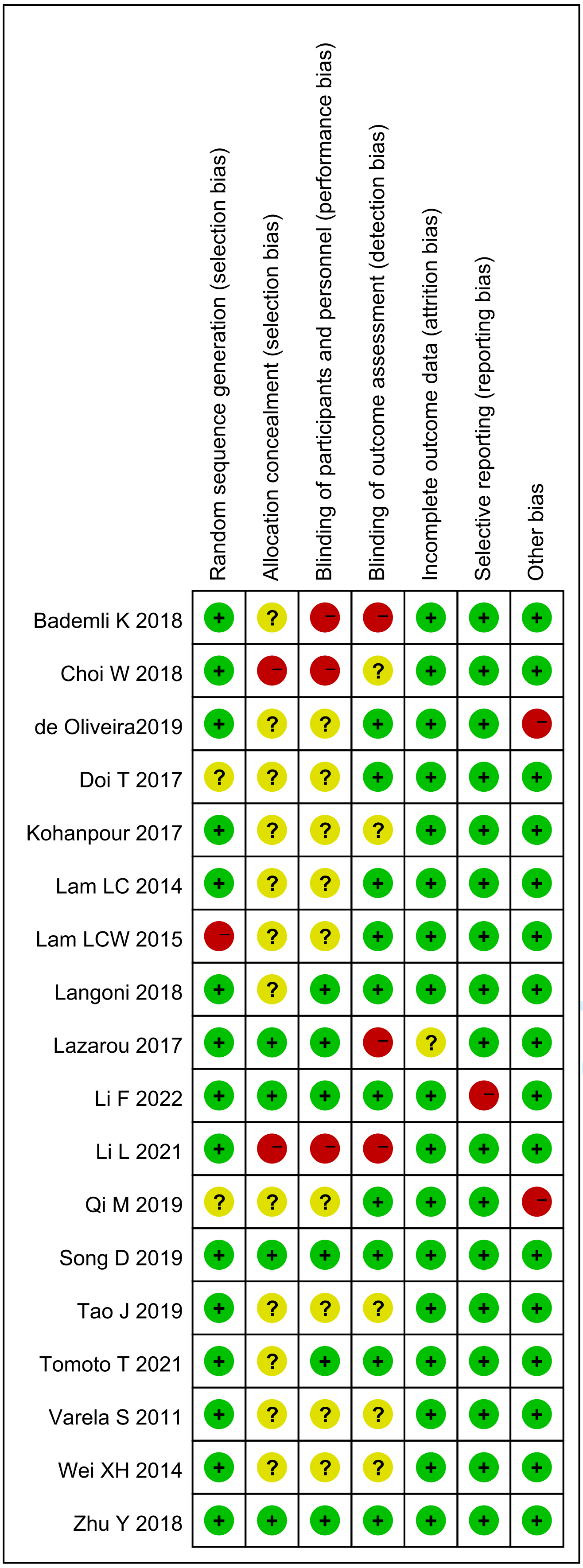


Figure 2: Risk of bias assessment of included trials.

| Study or Subgroup | Experimental Mean | Experimental SD | Experimental Total | Control Mean | Control SD | Control Total | Weight | Mean Difference IV, Fixed, 95% CI |
|---|---|---|---|---|---|---|---|---|
| Bademli K 2019 | 26.542 | 1.84 | 30 | 22.24 | 1.15 | 30 | 9.6% | 4.30 [3.53, 5.08] |
| de Oliveira2019 | 28.25 | 0.92 | 12 | 28.04 | 2.2 | 7 | 2.0% | 0.21 [-1.50, 1.92] |
| Doi T 2017 | 26.29 | 2.6 | 55 | 25.44 | 2.18 | 63 | 7.6% | 0.85 [-0.02, 1.72] |
| Kohanpour 2017 | 24.4 | 1.42 | 10 | 24.2 | 0.63 | 10 | 6.2% | 0.20 [-0.76, 1.16] |
| Lam LC 2014 | 25.4 | 3.3 | 96 | 24.2 | 3.4 | 169 | 8.3% | 1.20 [0.36, 2.04] |
| Lam LCW 2015 | 25.6 | 2.4 | 147 | 25.5 | 2.5 | 131 | 17.3% | 0.10 [-0.48, 0.68] |
| Langoni 2019 | 25 | 4.7 | 26 | 20.4 | 4.1 | 26 | 1.0% | 4.60 [2.20, 7.00] |
| Lazarou 2017 | 28 | 2.39 | 66 | 25.65 | 3.27 | 63 | 5.9% | 2.35 [1.36, 3.34] |
| Li L 2021 | 27.79 | 1.18 | 42 | 25.42 | 2.28 | 42 | 9.6% | 2.37 [1.59, 3.15] |
| Qi M 2019 | 28.2 | 1 | 16 | 27.3 | 1.7 | 16 | 6.2% | 0.90 [-0.07, 1.87] |
| Tomoto T 2021 | 28.5 | 1.46 | 22 | 28.5 | 1.5 | 30 | 8.8% | 0.00 [-0.81, 0.81] |
| Varela S 2012① | 20.66 | 7.39 | 17 | 19.53 | 5.5 | 15 | 0.3% | 1.13 [-3.35, 5.61] |
| Varela S 2012② | 21.06 | 5.4 | 16 | 19.53 | 5.5 | 15 | 0.4% | 1.53 [-2.31, 5.37] |
| Wei XH 2014 | 25.53 | 0.82 | 30 | 24.67 | 1.42 | 30 | 16.8% | 0.86 [0.27, 1.45] |
| **Total (95% CI)** | | | **585** | | | **647** | **100.0%** | **1.23 [0.99, 1.47]** |

Heterogeneity: Chi² = 112.89, df = 13 (P < 0.00001); I² = 88%
Test for overall effect: Z = 10.04 (P < 0.00001)

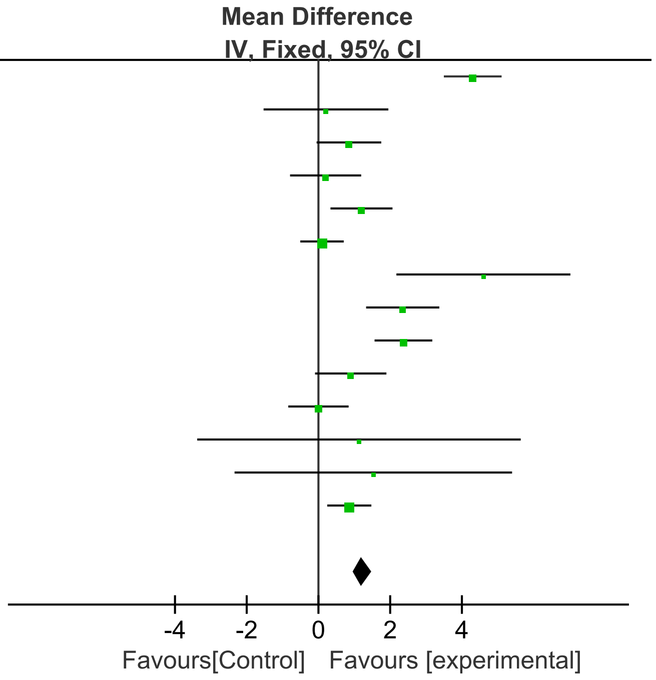


Figure 3: Effect sizes of forest plots for aerobic exercise versus control-MMSE outcomes. MMSE, mini-mental state examination

For peer review only

| Study or Subgroup | Experimental Mean | Experimental SD | Experimental Total | Control Mean | Control SD | Control Total | Weight | Mean Difference IV, Fixed, 95% CI |
|---|---|---|---|---|---|---|---|---|
| **2.2.1 multi-component aerobic exercise VS controls** | | | | | | | | |
| Bademli K 2019 | 26.542 | 1.84 | 30 | 22.24 | 1.15 | 30 | 9.6% | 4.30 [3.53, 5.08] |
| de Oliveira2019 | 28.25 | 0.92 | 12 | 28.04 | 2.2 | 7 | 2.0% | 0.21 [-1.50, 1.92] |
| Lam LCW 2015 | 25.6 | 2.4 | 147 | 25.5 | 2.5 | 131 | 17.3% | 0.10 [-0.48, 0.68] |
| Langoni 2019 | 25 | 4.7 | 26 | 20.4 | 4.1 | 26 | 1.0% | 4.60 [2.20, 7.00] |
| Li L 2021 | 27.79 | 1.18 | 42 | 25.42 | 2.28 | 42 | 9.6% | 2.37 [1.59, 3.15] |
| **Subtotal (95% CI)** | | | **257** | | | **236** | **39.5%** | **1.79 [1.41, 2.17]** |
| Heterogeneity: Chi² = 83.77, df = 4 (P < 0.00001); I² = 95% | | | | | | | | |
| Test for overall effect: Z = 9.18 (P < 0.00001) | | | | | | | | |
| **2.2.2 mind-body exercise VS controls** | | | | | | | | |
| Doi T 2017 | 26.29 | 2.6 | 55 | 25.44 | 2.18 | 63 | 7.6% | 0.85 [-0.02, 1.72] |
| Lam LC 2014 | 25.4 | 3.3 | 96 | 24.2 | 3.4 | 169 | 8.3% | 1.20 [0.36, 2.04] |
| Lazarou 2017 | 28 | 2.39 | 66 | 25.65 | 3.27 | 63 | 5.9% | 2.35 [1.36, 3.34] |
| Qi M 2019 | 28.2 | 1 | 16 | 27.3 | 1.7 | 16 | 6.2% | 0.90 [-0.07, 1.87] |
| **Subtotal (95% CI)** | | | **233** | | | **311** | **28.0%** | **1.28 [0.83, 1.74]** |
| Heterogeneity: Chi² = 6.03, df = 3 (P = 0.11); I² = 50% | | | | | | | | |
| Test for overall effect: Z = 5.51 (P < 0.00001) | | | | | | | | |
| **2.2.3 Conventional aerobic exercise VS controls** | | | | | | | | |
| Kohanpour 2017 | 24.4 | 1.42 | 10 | 24.2 | 0.63 | 10 | 6.2% | 0.20 [-0.76, 1.16] |
| Tomoto T 2021 | 28.5 | 1.46 | 22 | 28.5 | 1.5 | 30 | 8.8% | 0.00 [-0.81, 0.81] |
| Varela S 2012① | 20.66 | 7.39 | 17 | 19.53 | 5.5 | 15 | 0.3% | 1.13 [-3.35, 5.61] |
| Varela S 2012② | 21.06 | 5.4 | 16 | 19.53 | 5.5 | 15 | 0.4% | 1.53 [-2.31, 5.37] |
| Wei XH 2014 | 25.53 | 0.82 | 30 | 24.67 | 1.42 | 30 | 16.8% | 0.86 [0.27, 1.45] |
| **Subtotal (95% CI)** | | | **95** | | | **100** | **32.5%** | **0.51 [0.09, 0.93]** |
| Heterogeneity: Chi² = 3.62, df = 4 (P = 0.46); I² = 0% | | | | | | | | |
| Test for overall effect: Z = 2.38 (P = 0.02) | | | | | | | | |
| **Total (95% CI)** | | | **585** | | | **647** | **100.0%** | **1.23 [0.99, 1.47]** |
| Heterogeneity: Chi² = 112.89, df = 13 (P < 0.00001); I² = 88% | | | | | | | | |
| Test for overall effect: Z = 10.04 (P < 0.00001) | | | | | | | | |
| Test for subgroup differences: Chi² = 19.47, df = 2 (P < 0.0001), I² = 89.7% | | | | | | | | |

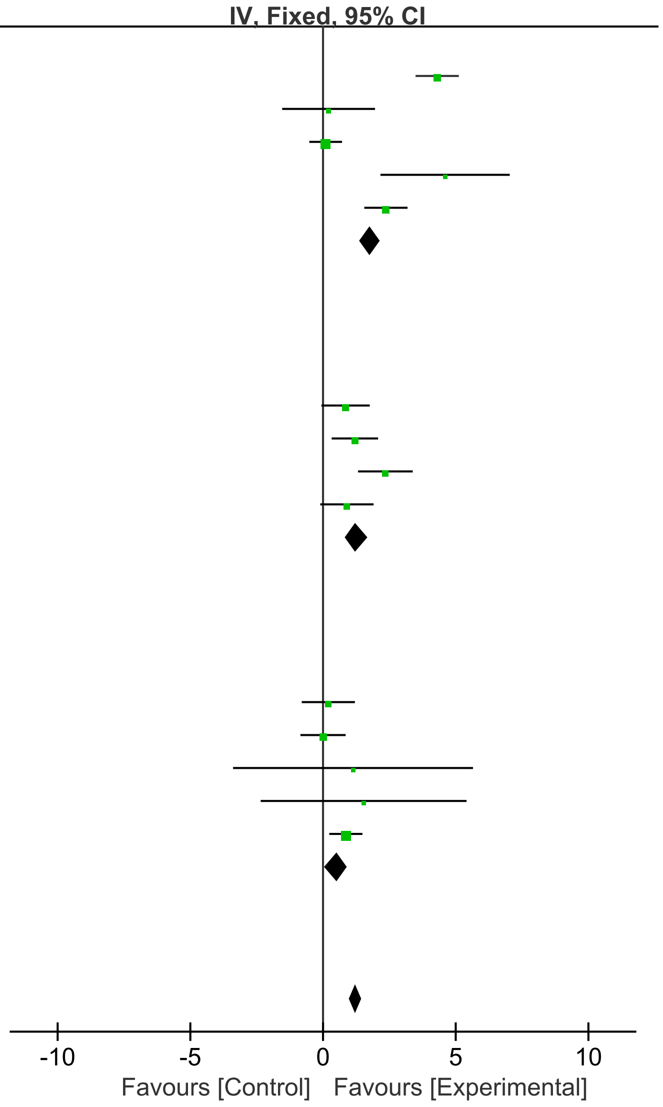


Figure 4: Effect sizes of forest plots for the different aerobic exercise-MMSE outcomes. MMSE, mini-mental state examination.

For peer review only

| Study or Subgroup | Experimental Mean | Experimental SD | Experimental Total | Control Mean | Control SD | Control Total | Weight | Mean Difference IV, Fixed, 95% CI |
|---|---|---|---|---|---|---|---|---|
| Choi W 2018 | 25.13 | 2.78 | 30 | 21.46 | 3.11 | 30 | 5.7% | 3.67 [2.18, 5.16] |
| Lazarou 2017 | 26 | 2.94 | 66 | 19.82 | 5.18 | 63 | 5.9% | 6.18 [4.72, 7.64] |
| Li F 2022 | 26.82 | 1.84 | 22 | 25.54 | 1.89 | 24 | 10.9% | 1.28 [0.20, 2.36] |
| [illegible] | [illegible] | [illegible] | [illegible] | [illegible] | [illegible] | [illegible] | [illegible] | [illegible] |

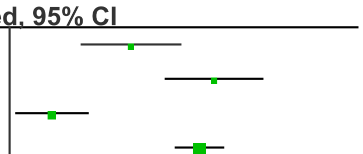

| Study or Subgroup | Experimental Mean | Experimental SD | Experimental Total | Control Mean | Control SD | Control Total | Weight | Mean Difference IV, Fixed, 95% CI |
|---|---|---|---|---|---|---|---|---|
| Choi W 2018 | 25.13 | 2.78 | 30 | 21.46 | 3.11 | 30 | 5.7% | 3.67 [2.18, 5.16] |
| Lazarou 2017 | 26 | 2.94 | 66 | 19.82 | 5.18 | 63 | 5.9% | 6.18 [4.72, 7.64] |
| Li F 2022 | 26.82 | 1.84 | 22 | 25.54 | 1.89 | 24 | 10.9% | 1.28 [0.20, 2.36] |
| Li L 2021 | 25.19 | 1.29 | 42 | 19.45 | 2 | 42 | 24.5% | 5.74 [5.02, 6.46] |
| Qi M 2019 | 24.3 | 2.2 | 16 | 23.7 | 2 | 16 | 6.0% | 0.60 [-0.86, 2.06] |
| Song D 2019 | 23.66 | 1.92 | 60 | 21.4 | 2.27 | 60 | 22.4% | 2.26 [1.51, 3.01] |
| Tao J 2019① | 24.55 | 2.33 | 20 | 22.1 | 2.59 | 20 | 5.4% | 2.45 [0.92, 3.98] |
| Tao J 2019② | 22.35 | 1.29 | 17 | 22.1 | 2.59 | 20 | 7.6% | 0.25 [-1.04, 1.54] |
| Zhu Y 2018 | 24.7 | 2.2 | 27 | 23.6 | 1.8 | 31 | 11.6% | 1.10 [0.06, 2.14] |
| **Total (95% CI)** | | | **300** | | | **306** | **100.0%** | **2.94 [2.58, 3.30]** |

Heterogeneity: Chi² = 129.08, df = 8 (P < 0.00001); $I^2$ = 94%
Test for overall effect: Z = 16.19 (P < 0.00001)

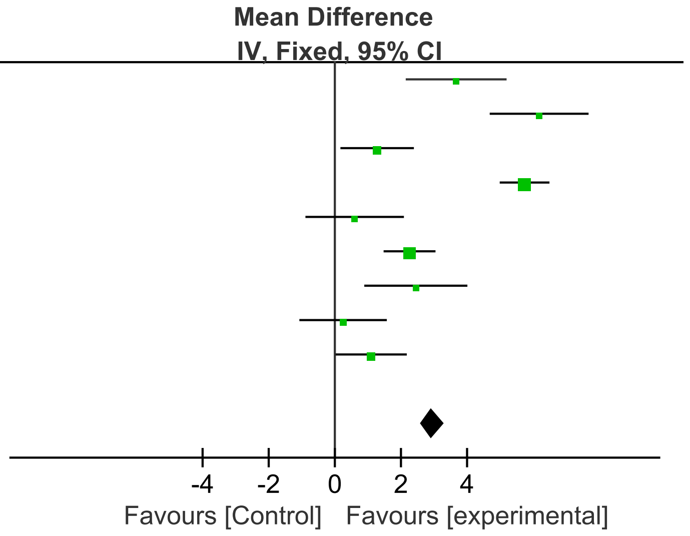


Figure 5: Effect sizes of forest plots for aerobic exercise versus controls-MoCA outcome. MoCA, Montreal Cognitive Assessment.

For peer review only

| Study or Subgroup | Experimental Mean | Experimental SD | Experimental Total | Control Mean | Control SD | Control Total | Weight | Mean Difference IV, Fixed, 95% CI |
|---|---|---|---|---|---|---|---|---|
| **3.2.1 multi-component aerobic exercise VS controls** | | | | | | | | |
| Li L 2021 | 25.19 | 1.29 | 42 | 19.45 | 2 | 42 | 26.0% | 5.74 [5.02, 6.46] |
| **Subtotal (95% CI)** | | | **42** | | | **42** | **26.0%** | **5.74 [5.02, 6.46]** |
| Heterogeneity: Not applicable | | | | | | | | |
| Test for overall effect: Z = 15.63 (P < 0.00001) | | | | | | | | |
| **3.2.2 mind-body exercise VS controls** | | | | | | | | |
| Li F 2022 | 26.82 | 1.84 | 22 | 25.54 | 1.89 | 24 | 11.6% | 1.28 [0.20, 2.36] |
| Qi M 2019 | 24.3 | 2.2 | 16 | 23.7 | 2 | 16 | 6.3% | 0.60 [-0.86, 2.06] |
| Tao J 2019① | 24.55 | 2.33 | 20 | 22.1 | 2.59 | 20 | 5.8% | 2.45 [0.92, 3.98] |
| Zhu Y 2018 | 24.7 | 2.2 | 27 | 23.6 | 1.8 | 31 | 12.4% | 1.10 [0.06, 2.14] |
| **Subtotal (95% CI)** | | | **85** | | | **91** | **36.1%** | **1.29 [0.67, 1.90]** |
| Heterogeneity: Chi² = 3.21, df = 3 (P = 0.36); I² = 6% | | | | | | | | |
| Test for overall effect: Z = 4.12 (P < 0.0001) | | | | | | | | |
| **3.2.3 Conventional aerobic exercise VS controls** | | | | | | | | |
| Choi W 2018 | 25.13 | 2.78 | 30 | 21.46 | 3.11 | 30 | 6.0% | 3.67 [2.18, 5.16] |
| Song D 2019 | 23.66 | 1.92 | 60 | 21.4 | 2.27 | 60 | 23.8% | 2.26 [1.51, 3.01] |
| Tao J 2019② | 22.35 | 1.29 | 17 | 22.1 | 2.59 | 20 | 8.1% | 0.25 [-1.04, 1.54] |
| **Subtotal (95% CI)** | | | **107** | | | **110** | **37.9%** | **2.06 [1.46, 2.65]** |
| Heterogeneity: Chi² = 12.30, df = 2 (P = 0.002); I² = 84% | | | | | | | | |
| Test for overall effect: Z = 6.76 (P < 0.00001) | | | | | | | | |
| **Total (95% CI)** | | | **234** | | | **243** | **100.0%** | **2.74 [2.37, 3.10]** |
| Heterogeneity: Chi² = 109.05, df = 7 (P < 0.00001); I² = 94% | | | | | | | | |
| Test for overall effect: Z = 14.61 (P < 0.00001) | | | | | | | | |
| Test for subgroup differences: Chi² = 93.54, df = 2 (P < 0.00001), I² = 97.9% | | | | | | | | |

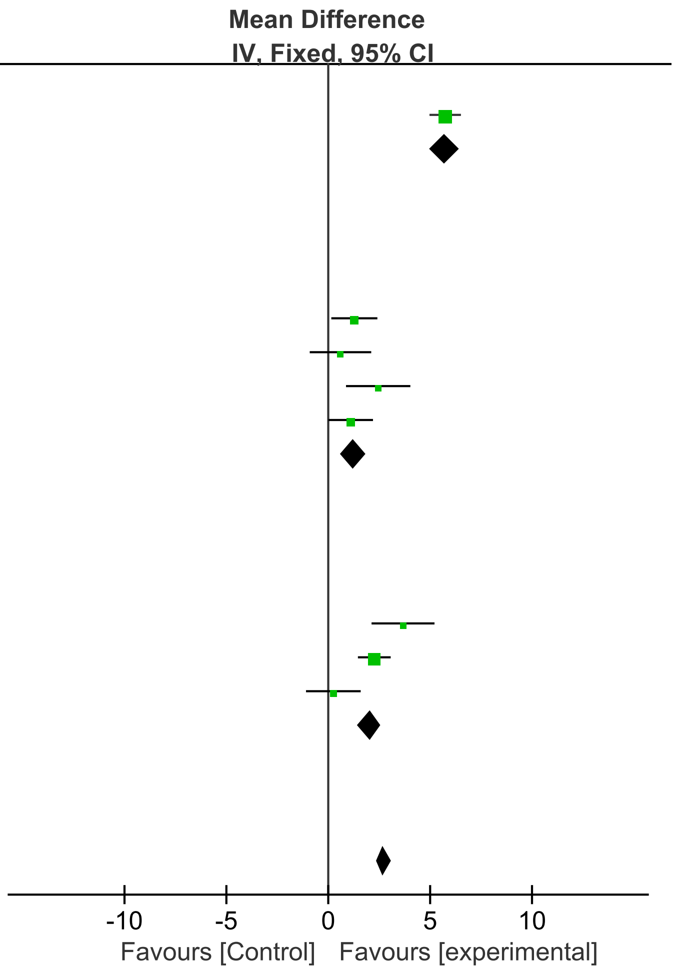


Figure 6: Effect sizes of forest plots for the different aerobic exercise-MoCA outcomes. MoCA, Montreal Cognitive Assessment

For peer review only

Supplementary table 1： Baseline characteristics of included trials

| Aerobic exercise type | study | region | | Participants | | | intervene | | | Measurement results |
|---|---|---|---|---|---|---|---|---|---|---|
| | | | | Sample size | age | female | manner | frequency | strength | |
| multi-component aerobic exercise | Li, L (2021)[12] | Asia/ China | Experimental group | 42 | 60–69（47.6%）、70–79（47.6%）、80 and above（4.8%） | 64% | 5 minutes warm-up, 5 minutes aerobic training, 5 minutes strength training, 5 minutes balance training, 5 minutes coordination training, 5 minutes sensitivity training | 30 minutes × 5 times× 24 weeks (6 months). | The warm-up is a 3-4/10 Perceptual Nu (RPE) level, and the rest of the workout is 4–5/10 RPE | MMSE MoCA |
| | | | Control group | 42 | 60–69（42.9%）、70–79（50.0%）、80 and above（7.1%） | 57% | General community health guidance | | | |
| | Bademli (2019)[15] | Asian/ Turkey | Experimental group | 30 | 72.24± 7.16 | 60% | 10 minutes of warm-up, 20 minutes of rhythmic exercise (including 22 simple exercises), 10 minutes of cooling exercise (low-intensity exercise), 40 minutes of free walking. | 80 minutes ×20 weeks, 4/3/7 days per week | 3-6 metabolic equivalents (ME). | MMSE |
| | | | Control group | 30 | 70.67 ± 8.34 | 57% | Continue with your daily activities | | | |
| | De Oliveira (2019)[14] | Euro-American/ Brazil | Experimental group | 7 | 71.85 ± 5.69 | 85.71% | 5 minutes of balance training,30 minutes of aerobic training, 20 minutes of strength training, 5 minutes of stretching exercises | 60 minutes × 2 times × 12 weeks | The intensity of the main training is 70% of the maximum oxygen consumption (VO2max) or 80% of the predicted maximum heart rate (HRmax). | MMSE |
| | | | Control group | 12 | 78.20± 5.26 | 41.66% | Clinical follow-up without any physical training | | | |
| | Langoni (2019)[13] | Euro-American/ Brazil | Experimental group | 26 | 72.6 ±7.8 | 76.9% | Strength training takes about30 minutes (with equipment including ankle weight, resistance latex tape and dumbbells) and aerobic training (20-30 minutes of walking). | 60 minutes× 2 times × 24 weeks | Aerobic training to walk at a maximum heart rate of 60% to 75%. | MMSE |
| | | | Control group | 26 | 71.9 ±7.9 | 76.9% | Do not initiate any form of physical or cognitive activity and keep in touch by phone | | | |
| | Lam (2015)[16] | Asia/ China | Experimental group | 147 | 75.5±6.7 | 76.9% | Stretching and conditioning exercises, mind-body exercises (such as tai chi) and aerobic exercises (such as static cycling). | 120 minutes× 3×48 weeks (12 months). | Unknown | MMSE |

For peer review only

| | | | | | | | | | | |
|---|---|---|---|---|---|---|---|---|---|---|
| | | | Control group | 131 | 75.4±6.1 | 77.9% | Social activities (e.g., tea parties, movie watching). | | | |
| mind-body exercise | Li,F (2022) | Euro-America/ United States | Experimental group | 22 | 74.5 ±5.6 | 36.4% | Traditional Tai Chi training | 60 minutes× 2 times × 16 weeks | The metabolic equivalent of Tai Chi exercise is 3.5-4.3 MET | MoCA |
| | | | Control group | 24 | 74.9±6.3 | 62.5% | Stretching exercises | | | |
| | Tao,J (2019)[10] ① | Asia/ China | Experimental group | 20 | 66.17±4.17 | 75% | 15 minutes to warm up, 40 minutes to eight danjin training and 5 minutes to cool down | 60 minutes × 3 times × 24 weeks | The Batdan Nishiki Movement MET is 2.68 | MoCA |
| | | | Control group | 20 | 65.97±5.66 | 70% | Maintain their original level of physical activity, do not receive any specific exercise interventions, health education | | | |
| | Qi,M (2019)[18] | Asia/ China | Experimental group | 16 | 70.6±6.2 | 69% | 35 minutes of moderate intensity aerobic dance (5 minutes of warm-up, 25 minutes of target heart rate dance and 5 minutes of music cooling). | 35 minutes × 3 times× 12 weeks (three months). | 60-80% maximum heart rate as the target heart rate for aerobic dance | MMSE MoCA |
| | | | Control group | 16 | 69.1±8.1 | 75% | Routine care | | | |
| | Zhu,Y (2018)[19] | Asia/ China | Experimental group | 29 | 70.3±6.7 | 51.7% | 5 minutes of warm-up, 25 minutes of target heart rate dance and 5 minutes of cooling | 35 minutes × 3 times× 12 weeks (three months | The target heart rate is set at 60%-80% of your maximum heart rate | MoCA |
| | | | Control group | 31 | 69.0±7.3 | 67.7% | Routine care | | | |
| | Lazarou, (2017)[20] | Euro-American/ Greece | Experimental group | 66 | 65.89± 10.76 | 80% | International Ballroom Dance (5 minutes of warm-up, review of previous dance lessons, 45 minutes of new material (tricks/dances), and 10 minutes of cooling time). | 60 minutes× 2 times× 40 weeks (10 months). | Dancing About 6 METs | MMSE MoCA |
| | | | Control group | 33 | 67.92±9.47 | 76% | No intervention was accepted, the educational meeting was received | | | |
| | Doi,T (2017)[21] | Asia/ Japan | Experimental group | 67 | 75.7±4.1 | 50.7% | The dance program is based on ballroom dances, including salsa, rumba, waltz, cha-cha, bruce, kitba and tango | 60 minutes × once × 40 weeks | Dancing About 6 METs | MMSE |
| | | | Control group | 67 | 76.0±4.9 | 46.3% | Health education class | | | |
| | Lam (2014)[22] | Asia/ China | Experimental group | 171 | 77.2±6.3 | 73% | 24 Styles of Tai Chi " Training. | During the introductory phase, the coaching process is 4 to 6 weeks. | The metabolic equivalent of Tai Chi exercise is 3.5-4.3 MET | MMSE |

For peer review only

| | | | | | | | | | | |
|---|---|---|---|---|---|---|---|---|---|---|
| | | | | | | | | During the maintenance phase, the frequency of interventions was ≥ 30 minutes per day and ≥ 3 days a week. Until the 1stmonth of the 2nd month | | |
| | | | Control group | 218 | 78.3±6.6 | 79% | Stretching and relaxation exercises | | | |
| Conventional aerobic exercise | Tomoto, (2021)[23] | Euro-American /USA | Experimental group | 22 | 64.8 ± 6.4 | 55% | Treadmill running | 1-10 weeks: 25-30 minutes × 3 times. 11-25 weeks: 30-35minutes× 3 or 4 times.26 weeks - 1 year: 30-40 minutes× 4-5 times | The intensity is 75%-85% of the maximum heart rate (HR). | MMSE |
| | | | Control group | 30 | 66. 1 ± 6.8 | 53% | Stretching and conditioning interventions | | | |
| | Song,D (2019)[24] | Asia/ China | Experimental group | 60 | 76.22 ±5.76 | 80% | Aerobic tread exercises | 60 minutes× 3 times × 16 weeks | For moderate-intensity workouts using the Borg Scale, the Borg score is 12-14 | MoCA |
| | | | Control group | 60 | 75.33 ±6.78 | 70% | Health education class | | | |
| | Tao,J (2019)[10] ② | Asia/ China | Experimental group | 17 | 64.32±2.60 | 59% | Warm up for 15 minutes, walk fast for 40 minutes, cool down for 5 minutes. | 60 minutes × 3 times × 24 weeks | The participants' heart rate was controlled at 55%-75% of the reserve heart rate | MoCA |
| | | | Control group | 20 | 65.97±5.6 | 70% | Maintain the original level of physical activity and do not receive any specific exercise interventions, health education | | | |
| | Choi,W (2018)[25] | Asia/ South Korea | Experimental group | 30 | 74.90±5.10 | 80% | 10min warm-up activity, 40min GKP exercise and 10min cooling activity. | 60 minutes× 2 times × 6 weeks | Unknown | MoCA |
| | | | Control group | 30 | 74.23±4.38 | 83% | Stretching, warm-up and cooling exercises are the same as in the exercise group | | | |
| | Kohanpour (2017)[26] | Asia/ Iran | Experimental group | 10 | 60-70 | 0% | 8min warm-up, (8-26)min running and cooling down for 5min | 21-39 minutes × 3 times × 12 weeks | The intensity is 75% to 85% of the maximum heart rate | MMSE |
| | | | Control group | 10 | 60-70 | 0% | Habitual lifestyle | | | |
| | Wei, X H (2014)[27] | Asia/ China | Experimental group | 30 | 66.73±5.48 | 30% | Handball training | 30 minutes × 5 times × 6 months (120 times). | The average intensity of aerobic exercise is about 60% of your maximum heart rate | MMSE |
| | | | Control group | 30 | 65.27±4.63 | 36.7% | Maintain the original way of life and entertainment | | | |

For peer review only

| | | | | | | | | | |
|---|---|---|---|---|---|---|---|---|---|
| Varela, S (2012)[11] ① | Euro-American/ Spain | Experimental group | 17 | 79.24± 10.07 | Unknown | 5 minutes of warm-up, 20 minutes of recumbent bike ride, and 5 minutes of stretching to cool down | 30 minutes × 3 times × 12 weeks | Exercise intensity is set at 40% of the participant's reserve heart rate (maximum heart rate - resting heart rate). | MMSE |
| | | Control group | 15 | 79.40 ±6.72 | | Social entertainment | | | |
| Varela, S (2012)[11] ② | Euro-American/ Spain | Experimental group | 16 | 76.44 ±11.38 | Unknown | 5 minutes of warm-up, 20 minutes of recumbent bike ride, and 5 minutes of stretching to cool down | 30 minutes × 3 times × 12 weeks | The exercise intensity is set at 60% of the participant's reserve heart rate (maximum heart rate - resting heart rate). | MMSE |
| | | Control group | 15 | 79.40 ±6.72 | | Social entertainment | | | |

For peer review only

Embase®

# Embase Session Results (23 Mar 2022)

| No. | Query | Results |
|---|---|---|
| #22 | ((('aerobic exercise'/exp OR ('aerobic dance':ab,ti OR 'aerobic dancing':ab,ti OR aerobics:ab,ti OR 'aerobics exercise':ab,ti OR 'dancing, aerobic':ab,ti OR 'exercise, aerobic':ab,ti OR 'low impact aerobic exercise':ab,ti OR 'low impact aerobics':ab,ti OR 'step aerobics':ab,ti)) OR ('exercise'/exp OR ('biometric exercise':ab,ti OR effort:ab,ti OR 'exercise capacity':ab,ti OR 'exercise performance':ab,ti OR 'exercise training':ab,ti OR exertion:ab,ti OR 'fitness training':ab,ti OR 'fitness workout':ab,ti OR 'physical conditioning':ab,ti OR human:ab,ti OR 'physical effort':ab,ti OR 'physical exercise':ab,ti OR 'physical exertion':ab,ti OR 'physical work-out':ab,ti OR 'physical workout':ab,ti)) OR ('physical activity'/exp OR 'activity, physical':ab,ti)) AND (('cognitive defect'/exp OR ('cognition disorders':ab,ti OR 'cognitive defects':ab,ti OR 'cognitive deficit':ab,ti OR 'cognitive disability':ab,ti OR 'cognitive disorder':ab,ti OR 'cognitive disorders':ab,ti OR 'cognitive dysfunction':ab,ti OR 'cognitive impairment':ab,ti OR 'delirium, dementia, amnestic, cognitive disorders':ab,ti OR overinclusion:ab,ti OR 'response interference':ab,ti)) OR ('mild cognitive impairment'/exp OR 'amnestic mild cognitive impairment':ab,ti))) AND ('aged'/exp OR ('aged patient':ab,ti OR 'aged people':ab,ti OR 'aged person':ab,ti OR 'aged subject':ab,ti OR elderly:ab,ti OR 'elderly patient':ab,ti OR 'elderly people':ab,ti OR 'elderly person':ab,ti OR 'elderly subject':ab,ti OR 'senior citizen':ab,ti OR senium:ab,ti)) | 16100 |
| #21 | 'aged'/exp OR ('aged patient':ab,ti OR 'aged people':ab,ti OR 'aged person':ab,ti OR 'aged subject':ab,ti OR elderly:ab,ti OR 'elderly patient':ab,ti OR 'elderly people':ab,ti OR 'elderly person':ab,ti OR 'elderly subject':ab,ti OR 'senior citizen':ab,ti OR senium:ab,ti) | 3555832 |
| #20 | 'aged patient':ab,ti OR 'aged people':ab,ti OR 'aged person':ab,ti OR 'aged subject':ab,ti OR elderly:ab,ti OR 'elderly patient':ab,ti OR 'elderly people':ab,ti OR 'elderly person':ab,ti OR 'elderly subject':ab,ti OR 'senior citizen':ab,ti OR senium:ab,ti | 385314 |
| #19 | 'aged'/exp | 3469661 |
| #18 | (('aerobic exercise'/exp OR ('aerobic dance':ab,ti OR 'aerobic dancing':ab,ti OR aerobics:ab,ti OR 'aerobics exercise':ab,ti OR 'dancing, aerobic':ab,ti OR 'exercise, aerobic':ab,ti OR 'low impact aerobic exercise':ab,ti OR 'low impact aerobics':ab,ti OR 'step aerobics':ab,ti)) OR ('exercise'/exp OR ('biometric exercise':ab,ti OR effort:ab,ti OR 'exercise capacity':ab,ti OR 'exercise performance':ab,ti OR 'exercise training':ab,ti OR exertion:ab,ti OR 'fitness training':ab,ti OR 'fitness workout':ab,ti OR 'physical conditioning':ab,ti OR human:ab,ti OR 'physical effort':ab,ti OR 'physical exercise':ab,ti OR 'physical exertion':ab,ti OR 'physical work-out':ab,ti OR 'physical workout':ab,ti)) OR ('physical activity'/exp OR 'activity, physical':ab,ti)) AND (('cognitive defect'/exp OR ('cognition disorders':ab,ti OR 'cognitive defects':ab,ti OR 'cognitive deficit':ab,ti OR 'cognitive disability':ab,ti OR 'cognitive disorder':ab,ti OR 'cognitive disorders':ab,ti OR 'cognitive dysfunction':ab,ti OR 'cognitive impairment':ab,ti OR 'delirium, dementia, amnestic, cognitive disorders':ab,ti OR overinclusion:ab,ti OR 'response interference':ab,ti)) OR ('mild cognitive impairment'/exp OR 'amnestic mild cognitive impairment':ab,ti)) | 76351 |
| #17 | ('cognitive defect'/exp OR ('cognition disorders':ab,ti OR 'cognitive defects':ab,ti OR 'cognitive deficit':ab,ti OR 'cognitive disability':ab,ti OR 'cognitive disorder':ab,ti OR 'cognitive disorders':ab,ti OR 'cognitive dysfunction':ab,ti OR 'cognitive impairment':ab,ti OR 'delirium, dementia, amnestic, cognitive disorders':ab,ti OR overinclusion:ab,ti OR 'response interference':ab,ti)) OR ('mild cognitive impairment'/exp OR 'amnestic mild cognitive impairment':ab,ti) | 577692 |
| #16 | ('aerobic exercise'/exp OR ('aerobic dance':ab,ti OR 'aerobic dancing':ab,ti OR aerobics:ab,ti OR 'aerobics exercise':ab,ti OR 'dancing, aerobic':ab,ti OR 'exercise, aerobic':ab,ti OR 'low impact aerobic exercise':ab,ti OR 'low impact aerobics':ab,ti OR 'step aerobics':ab,ti)) OR ('exercise'/exp OR ('biometric exercise':ab,ti OR effort:ab,ti OR 'exercise capacity':ab,ti OR 'exercise performance':ab,ti OR 'exercise training':ab,ti OR exertion:ab,ti OR 'fitness training':ab,ti OR 'fitness workout':ab,ti OR 'physical conditioning':ab,ti OR human:ab,ti OR 'physical effort':ab,ti OR 'physical exercise':ab,ti OR 'physical exertion':ab,ti OR 'physical work-out':ab,ti OR 'physical workout':ab,ti)) OR ('physical activity'/exp OR 'activity, physical':ab,ti) | 4258734 |
| #15 | 'mild cognitive impairment'/exp OR 'amnestic mild cognitive impairment':ab,ti | 32561 |
| #14 | 'amnestic mild cognitive impairment':ab,ti | 3430 |
| #13 | 'mild cognitive impairment'/exp | 32022 |
| #12 | 'cognitive defect'/exp OR ('cognition disorders':ab,ti OR 'cognitive defects':ab,ti OR 'cognitive deficit':ab,ti OR 'cognitive disability':ab,ti OR 'cognitive disorder':ab,ti OR 'cognitive disorders':ab,ti OR 'cognitive dysfunction':ab,ti OR 'cognitive impairment':ab,ti OR 'delirium, dementia, amnestic, cognitive disorders':ab,ti OR overinclusion:ab,ti OR 'response interference':ab,ti) | 577692 |
| | 'cognition disorders':ab,ti OR 'cognitive defects':ab,ti OR 'cognitive deficit':ab,ti OR 'cognitive disability':ab,ti | |

| | | |
|---|---|---|
| #11 | OR 'cognitive disorder':ab,ti OR 'cognitive disorders':ab,ti OR 'cognitive dysfunction':ab,ti OR 'cognitive impairment':ab,ti OR 'delirium, dementia, amnestic, cognitive disorders':ab,ti OR overinclusion:ab,ti OR 'response interference':ab,ti | 136389 |
| #10 | 'cognitive defect'/exp | 557350 |
| #9 | 'physical activity'/exp OR 'activity, physical':ab,ti | 486507 |
| #8 | 'activity, physical':ab,ti | 1355 |
| #7 | 'physical activity'/exp | 485896 |
| #6 | 'exercise'/exp OR ('biometric exercise':ab,ti OR effort:ab,ti OR 'exercise capacity':ab,ti OR 'exercise performance':ab,ti OR 'exercise training':ab,ti OR exertion:ab,ti OR 'fitness training':ab,ti OR 'fitness workout':ab,ti OR 'physical conditioning':ab,ti OR human:ab,ti OR 'physical effort':ab,ti OR 'physical exercise':ab,ti OR 'physical exertion':ab,ti OR 'physical work-out':ab,ti OR 'physical workout':ab,ti) | 3884161 |
| #5 | 'biometric exercise':ab,ti OR effort:ab,ti OR 'exercise capacity':ab,ti OR 'exercise performance':ab,ti OR 'exercise training':ab,ti OR exertion:ab,ti OR 'fitness training':ab,ti OR 'fitness workout':ab,ti OR 'physical conditioning':ab,ti OR human:ab,ti OR 'physical effort':ab,ti OR 'physical exercise':ab,ti OR 'physical exertion':ab,ti OR 'physical work-out':ab,ti OR 'physical workout':ab,ti | 3576566 |
| #4 | 'exercise'/exp | 399161 |
| #3 | 'aerobic exercise'/exp OR ('aerobic dance':ab,ti OR 'aerobic dancing':ab,ti OR aerobics:ab,ti OR 'aerobics exercise':ab,ti OR 'dancing, aerobic':ab,ti OR 'exercise, aerobic':ab,ti OR 'low impact aerobic exercise':ab,ti OR 'low impact aerobics':ab,ti OR 'step aerobics':ab,ti) | 20255 |
| #2 | 'aerobic dance':ab,ti OR 'aerobic dancing':ab,ti OR aerobics:ab,ti OR 'aerobics exercise':ab,ti OR 'dancing, aerobic':ab,ti OR 'exercise, aerobic':ab,ti OR 'low impact aerobic exercise':ab,ti OR 'low impact aerobics':ab,ti OR 'step aerobics':ab,ti | 1495 |
| #1 | 'aerobic exercise'/exp | 19335 |

ELSEVIER

© 2022 Elsevier Life Sciences IP Limited except certain content provided by third parties.
Embase is a trade mark of Elsevier Life Sciences IP Limited. RELX Group and the RE symbol are trade marks of RELX Group plc, used under license.

For peer review only - http://bmjopen.bmj.com/site/about/guidelines.xhtml

# PRISMA 2020 Checklist

| Section and Topic | Item # | Checklist item | Location where item is reported |
|---|---|---|---|
| **TITLE** | | | |
| Title | 1 | Identify the report as a systematic review. | Page 1 |
| **ABSTRACT** | | | |
| Abstract | 2 | See the PRISMA 2020 for Abstracts checklist. | Page 2 |
| **INTRODUCTION** | | | |
| Rationale | 3 | Describe the rationale for the review in the context of existing knowledge. | Page 2 |
| Objectives | 4 | Provide an explicit statement of the objective(s) or question(s) the review addresses. | Page 1 |
| **METHODS** | | | |
| Eligibility criteria | 5 | Specify the inclusion and exclusion criteria for the review and how studies were grouped for the syntheses. | Page 2 |
| Information sources | 6 | Specify all databases, registers, websites, organisations, reference lists and other sources searched or consulted to identify studies. Specify the date when each source was last searched or consulted. | Page 2 and figure |
| Search strategy | 7 | Present the full search strategies for all databases, registers and websites, including any filters and limits used. | supplementary statement |
| Selection process | 8 | Specify the methods used to decide whether a study met the inclusion criteria of the review, including how many reviewers screened each record and each report retrieved, whether they worked independently, and if applicable, details of automation tools used in the process. | Page 2 |
| Data collection process | 9 | Specify the methods used to collect data from reports, including how many reviewers collected data from each report, whether they worked independently, any processes for obtaining or confirming data from study investigators, and if applicable, details of automation tools used in the process. | Page 3 |
| Data items | 10a | List and define all outcomes for which data were sought. Specify whether all results that were compatible with each outcome domain in each study were sought (e.g. for all measures, time points, analyses), and if not, the methods used to decide which results to collect. | Page 3 |
| | 10b | List and define all other variables for which data were sought (e.g. participant and intervention characteristics, funding sources). Describe any assumptions made about any missing or unclear information. | Page 2 |
| Study risk of bias assessment | 11 | Specify the methods used to assess risk of bias in the included studies, including details of the tool(s) used, how many reviewers assessed each study and whether they worked independently, and if applicable, details of automation tools used in the process. | Page 3 |
| Effect measures | 12 | Specify for each outcome the effect measure(s) (e.g. risk ratio, mean difference) used in the synthesis or presentation of results. | Page 3 |
| Synthesis methods | 13a | Describe the processes used to decide which studies were eligible for each synthesis (e.g. tabulating the study intervention characteristics and comparing against the planned groups for each synthesis (item #5)). | Page 2 |
| | 13b | Describe any methods required to prepare the data for presentation or synthesis, such as handling of missing summary statistics, or data conversions. | Page 3 |
| | 13c | Describe any methods used to tabulate or visually display results of individual studies and syntheses. | Page 3 |
| | 13d | Describe any methods used to synthesize results and provide a rationale for the choice(s). If meta-analysis was performed, describe the model(s), method(s) to identify the presence and extent of statistical heterogeneity, and software package(s) used. | Page 3 |
| | 13e | Describe any methods used to explore possible causes of heterogeneity among study results (e.g. subgroup analysis, meta-regression). | Page 3 |
| | 13f | Describe any sensitivity analyses conducted to assess robustness of the synthesized results. | Page 3 |
| Reporting bias assessment | 14 | Describe any methods used to assess risk of bias due to missing results in a synthesis (arising from reporting biases). | -( Does not meet the number of |

For peer review only - http://bmjopen.bmj.com/site/about/guidelines.xhtml

## PRISMA 2020 Checklist

| Section and Topic | Item # | Checklist item | Location where item is reported |
|---|---|---|---|
| | | | studies required for funnel plots) |
| Certainty assessment | 15 | Describe any methods used to assess certainty (or confidence) in the body of evidence for an outcome. | Page 8 |
| **RESULTS** | | | |
| Study selection | 16a | Describe the results of the search and selection process, from the number of records identified in the search to the number of studies included in the review, ideally using a flow diagram. | Figure 1 |
| | 16b | Cite studies that might appear to meet the inclusion criteria, but which were excluded, and explain why they were excluded. | Figure 1 |
| Study characteristics | 17 | Cite each included study and present its characteristics. | Table 2 |
| Risk of bias in studies | 18 | Present assessments of risk of bias for each included study. | Figure 2 |
| Results of individual studies | 19 | For all outcomes, present, for each study: (a) summary statistics for each group (where appropriate) and (b) an effect estimate and its precision (e.g. confidence/credible interval), ideally using structured tables or plots. | All figure |
| Results of syntheses | 20a | For each synthesis, briefly summarise the characteristics and risk of bias among contributing studies. | Page 8 |
| | 20b | Present results of all statistical syntheses conducted. If meta-analysis was done, present for each the summary estimate and its precision (e.g. confidence/credible interval) and measures of statistical heterogeneity. If comparing groups, describe the direction of the effect. | Figure 3-6<br>Page 8-10 |
| | 20c | Present results of all investigations of possible causes of heterogeneity among study results. | Page 8<br>Figure 2 |
| | 20d | Present results of all sensitivity analyses conducted to assess the robustness of the synthesized results. | Figure 11 |
| Reporting biases | 21 | Present assessments of risk of bias due to missing results (arising from reporting biases) for each synthesis assessed. | - |
| Certainty of evidence | 22 | Present assessments of certainty (or confidence) in the body of evidence for each outcome assessed. | Table 4 |
| **DISCUSSION** | | | |
| Discussion | 23a | Provide a general interpretation of the results in the context of other evidence. | Page 10 |
| | 23b | Discuss any limitations of the evidence included in the review. | Page 11 |
| | 23c | Discuss any limitations of the review processes used. | Page 11 |
| | 23d | Discuss implications of the results for practice, policy, and future research. | Page 11 |
| **OTHER INFORMATION** | | | |
| Registration and protocol | 24a | Provide registration information for the review, including register name and registration number, or state that the review was not registered. | Page 2 |
| | 24b | Indicate where the review protocol can be accessed, or state that a protocol was not prepared. | - |
| | 24c | Describe and explain any amendments to information provided at registration or in the protocol. | -( Not modified) |
| Support | 25 | Describe sources of financial or non-financial support for the review, and the role of the funders or sponsors in the review. | Page 15 |
| Competing | 26 | Declare any competing interests of review authors. | - |

For peer review only - http://bmjopen.bmj.com/site/about/guidelines.xhtml

# PRISMA 2020 Checklist

| Section and Topic | Item # | Checklist item | Location where item is reported |
|---|---|---|---|
| interests | | | |
| Availability of data, code and other materials | 27 | Report which of the following are publicly available and where they can be found: template data collection forms; data extracted from included studies; data used for all analyses; analytic code; any other materials used in the review. | Page 2 and Page 3 |

*From:* Page MJ, McKenzie JE, Bossuyt PM, Boutron I, Hoffmann TC, Mulrow CD, et al. The PRISMA 2020 statement: an updated guideline for reporting systematic reviews. BMJ 2021;372:n71. doi: 10.1136/bmj.n71

For more information, visit: http://www.prisma-statement.org/

For peer review only

BMJ Open

# Effects of different aerobic exercises on the global cognitive function of the elderly with mild cognitive impairment: a meta-analysis

| | |
|---|---|
| Journal: | *BMJ Open* |
| Manuscript ID | bmjopen-2022-067293.R1 |
| Article Type: | Original research |
| Date Submitted by the Author: | 22-Feb-2023 |
| Complete List of Authors: | Han, Conglin; Weifang Medical University; Beijing Rehabilitation Hospital,<br><br>Sun, Weishuang; Weifang Medical University<br>Zhang, Dan; Weifang Medical University<br>Xi, Xiaoshuang; Capital Medical University<br>Zhang, Rong; Yunnan University of Chinese Medicine<br>Gong, Weijun; Capital Medical University |
| <b>Primary Subject Heading</b>: | Rehabilitation medicine |
| Secondary Subject Heading: | Sports and exercise medicine, Geriatric medicine |
| Keywords: | NEUROLOGY, SPORTS MEDICINE, Aging, Aged |
| | |

SCHOLARONE™ Manuscripts

*I, the Submitting Author has the right to grant and does grant on behalf of all authors of the Work (as defined in the below author licence), an exclusive licence and/or a non-exclusive licence for contributions from authors who are: i) UK Crown employees; ii) where BMJ has agreed a CC-BY licence shall apply, and/or iii) in accordance with the terms applicable for US Federal Government officers or employees acting as part of their official duties; on a worldwide, perpetual, irrevocable, royalty-free basis to BMJ Publishing Group Ltd ("BMJ") its licensees and where the relevant Journal is co-owned by BMJ to the co-owners of the Journal, to publish the Work in this journal and any other BMJ products and to exploit all rights, as set out in our licence.*

*The Submitting Author accepts and understands that any supply made under these terms is made by BMJ to the Submitting Author unless you are acting as an employee on behalf of your employer or a postgraduate student of an affiliated institution which is paying any applicable article publishing charge ("APC") for Open Access articles. Where the Submitting Author wishes to make the Work available on an Open Access basis (and intends to pay the relevant APC), the terms of reuse of such Open Access shall be governed by a Creative Commons licence – details of these licences and which Creative Commons licence will apply to this Work are set out in our licence referred to above.*

*Other than as permitted in any relevant BMJ Author's Self Archiving Policies, I confirm this Work has not been accepted for publication elsewhere, is not being considered for publication elsewhere and does not duplicate material already published. I confirm all authors consent to publication of this Work and authorise the granting of this licence.*

For peer review only

# Effects of different aerobic exercises on the global cognitive function of the elderly with mild cognitive impairment: a meta-analysis

Conglin Han[1], Weishuang Sun[1], Dan Zhang[1] , Xiaoshuang Xi[2], Rong Zhang[3], Weijun Gong[4]*

1 Beijing Rehabilitation Hospital, Capital Medical University, Beijing 100144, China
Rehabilitation Medicine Academy, Weifang Medical University, Shandong 261053, China
2 Beijing Rehabilitation Medicine Academy, Capital Medical University, Beijing 100144, China
3 Second Clinical Medical Academy, Yunnan University of Chinese Medicine, Yunnan 650500, China
4 Department of Neurological Rehabilitation, Beijing Rehabilitation Hospital, Capital Medical University, Beijing 100144, China
*Corresponding author: Weijun Gong, Correspondence: E-mail: gwj197104@ccmu.edu.cn.

ORCID：0000-0002-9134-8218 (Weijun Gong)

**Abstract**

OBJECTIVE: To summarize the effects of various types of aerobic exercise on the global cognitive function of the elderly with mild cognitive impairment(MCI).

METHODS: Electronic databases of PubMed, EMBASE, and the Cochrane Library, clinical trial registry websites, and clinical randomized controlled trials published in peer-reviewed journals were searched through March 2022. For included studies, differences in endpoint values for global cognitive function were analyzed across studies based on the outcome of the Simple Mental State Examination Scale (MMSE) or the Montreal Cognitive Assessment Scale (MoCA). A paired meta-analysis using a random effects model was performed to directly test the efficacy of different types of exercise.

RESULTS: A total of 1680 patients who participated in 20 randomized controlled trials were included in this study. Based on outcomes of MMSE analysis, the aerobic exercise, which was beneficial for global cognitive function in MCI patients, included multi-component aerobic exercise (MD=1.79, 95% CI [1.41, 2.17], $p<0.01$), mind-body exercise (MD=1.29, 95% CI [0.67, 1.90], $p<0.01$) and conventional aerobic exercise MD=0.51, 95% CI [0.09, 0.93 ], $p<0.05$). With the evaluation of MoCA, multi-component aerobic exercise (MD=5.54,95% CI [5.02, 6.46], $p<0.01$), mind-body exercise (MD=1.29,95% CI [0.67, 1.90], $p<0.01$) and conventional aerobic exercise (MD=2.06,95% CI [1.46, 2.65], $p< 0.01$) were showed significant beneficial effects for the patient. However, there was a high degree of heterogeneity between the results of multi-component aerobic exercise (MMSE) and conventional aerobic exercise (MoCA), which was analyzed and explored.

CONCLUSIONS: In general, multi-component aerobic exercise, mind-body exercise, and conventional aerobic exercise were beneficial in improving global cognitive function in the elderly with mild cognitive impairment. Nevertheless, the improvement effect of mind-body exercise is more reliable compared to multi-component aerobic exercise and conventional aerobic exercise.

Keywords: Mild cognitive impairment, aerobic exercise, elderly

For peer review only

**Strengths and limitations of this study**

- This is a comprehensive meta-analysis of aerobic exercise interventions for mild cognitive impairment (MCI), with a particular focus on the different types of aerobic exercise types individually.
- By analyzing the results of overall cognitive function, we concluded that all types of aerobic exercise were beneficial for the cognitive function of MCI patients.
- This meta-analysis explores and highlights the heterogeneity of some of the results and important limitations in the literature, such as older and female MCI patients, who may have poorer outcomes for aerobic exercise interventions.

## 1. Introduction

Along with the development and improvement in modern science, medical technology, and treatment, the average life expectancy of people around the world has been continuously extended. Age-related health problems are becoming more and more prominent. Dementia is one of the common diseases of aging and a major cause of disability in the elderly[1]. The World Health Organization estimates that about 50 million people worldwide suffer from dementia, with a new case every three seconds, and the number of people with dementia will triple by 2050[2]. Severe dementia can place a huge emotional and financial burden on families and society, so it is important to take measures for early detection, early intervention, and early treatment of dementia. Mild cognitive impairment (MCI) is an intermediate state between normal cognition and dementia. MCI usually refers to cognitive decline beyond that caused by normal aging, but without significant impairment in Activity of Daily Living (ADL)[3]. Currently, medications have a limited role in preventing and treating MCI[4,5], so there is a large body of research focusing on non-pharmacological interventions.

It has been suggested that some non-pharmacological interventions, such as food, exercise, and cognitive stimulation, may be beneficial for the improvement of cognitive function in MCI patients[3,4,6]. It has also shown that physical activity has a greater influence on the cognitive performance of MCI patients than drugs. For the elderly population with a decline in cognitive function, several studies have also confirmed that aerobic exercise appears to be effective in improving cognitive function[7-9]. However, there are various types of aerobic exercise, and the aerobic exercise that can be performed may vary in different populations (For example, it is difficult for people with lower limb mobility restrictions to run). As a result, an analysis is needed to compare the effects of different types of aerobic exercise on the improvement of global cognitive function in the elderly with MCI.

## 2. Methods

This review has been registered in the International Prospective Systematic Evaluation Registry database (PROSPERO, http://www.cdr.york.ac.uk/PROSPERO/) under the registration number: CRD42022327386. We performed a meta-analysis according to the PRISMA guidelines.

### 2.1. Inclusion criteria

Types of studies: Human subject studies designed as randomized controlled trials (RCT) published in English were included without year restriction.

Types of participants: Subjects defined as MCI by the original authors and aged >60 years were included. We excluded healthy aging elderly adults, patients with any form of dementia, or those diagnosed with cognitive impairment due to definite etiologies, such as trauma or vascular or psychiatric diseases. Any clinical subtype of MCI was considered eligible.

Types of interventions: We included supervised structured exercises of any frequency, intensity, duration, or time directed at improving physical fitness. The interventions were various forms of exercise containing aerobic exercise, including conventional aerobic exercise (simple and common aerobic exercises such as running, brisk walking, and cycling), mind-body exercise (Mind–body exercise focuses on mind, body, psychology, and behavior, including breathing and physical exercise, meditation, and so on[10]. It is a type of aerobic exercise of low to moderate intensity, with common forms such as tai chi, yoga, and dance[11,12].), and multi-component aerobic exercise (aerobic exercise combined with other forms of exercise, such as resistance training, balance training). Aerobic exercise was defined as the involvement of major muscle groups throughout the body in addition to the primary involvement of oxygen for energy supply.

Types of control groups: Controls could be exercises of stretching, activities of health education, routine care, daily lifestyle, and social recreation.

Types of outcomes: The results are presented using measurable cognitive screening instruments: the Mini-Mental State Examination (MMSE) and the Montreal Cognitive Assessment (MoCA). and are available as mean (M) and standard deviation (SD).

### 2.2. Exclusion criteria

Intervention methods could only be a variety of exercise training modalities; those with specific instructions to have other training components, such as cognitive training, were excluded. Non-intervention studies, articles with only research protocols, review articles, unpublished studies, abstracts or papers, and articles for which full text or data were not available were excluded. Non-English articles were excluded.

### 2.3. Search strategy

We systematically searched the PubMed, EMBASE, and Cochrane Library electronic databases, clinical trial registration websites, and clinical randomized controlled trials published in peer-reviewed journals from the earliest available record until March 2022. We searched using subject terms and free words. The keywords used included search terms related to sports exercise (e.g., exercise or physical activity or aerobic exercise) and cognitive impairment (e.g., mild cognitive impairment or cognitive dysfunction or amnestic mild cognitive impairment), and search terms related to older adults ( e.g., older adults or older patients). The search strategies for Embase, Pubmed and the Cochrane Library are described in Supplementary Document 1. Endnote X9.1 was used to store and sort the retrieved randomized controlled trials and to remove duplicates. Initial screening was performed by the first author based on the title and abstract.



For peer review only - http://bmjopen.bmj.com/site/about/guidelines.xhtml

This was followed by an independent screening of the full text by two reviewers according to the eligibility criteria specified in the study protocol. Disagreements in included studies were resolved by negotiation.

**2.4. Data extraction and analysis**

A standardized data extraction form was applied to extract data based on the following parameters: (1) basic information about the included studies (author, year, country) (2) basic characteristics of the study population (sample size, age, and gender of participants) (3) intervention characteristics (type of intervention, the intervention specifics, frequency and intensity of the intervention) (4) Outcome measures: we chose the two most commonly used scales for assessing global cognitive functioning, the MMSE and the MoCA. We performed a meta-analysis using the endpoint measures.

For each outcome, the effect sizes are reported as the mean difference with 95% confidence intervals (CI) for each study. We pooled these values and conducted the meta-analysis using the Review Manager (RevMan) version 5.3. Heterogeneity between trial design options was unclear; therefore, a random-effects model was chosen. The $I^2$ statistic was computed as a measure of heterogeneity[13]. Cochrane's handbook recommends heterogeneity as not important (0–40%), moderate heterogeneity (30–60%), substantial heterogeneity (50–90%), and considerable heterogeneity (75–100%). Subgroup or sensitivity analyses were used to address significant clinical heterogeneity, or only descriptive analyses were performed.

**2.5. Risk of Bias and quality assessment**

The Cochrane risk of bias assessment tool was used to independently assess the risk of bias in included trials. The assessment covered the following parameters: random sequence generation, allocation concealment, blinding, incomplete data, selective reporting, and others ( Cochrane Handbook for Systematic Reviews of Interventions 8.5 ) . Each parameter was divided into three categories: low risk, unclear, and high risk. Each study was categorized using the following criteria: low risk of bias (all parameters were rated as low risk); moderate risk of bias (with parameters rated as unclear and no parameters rated as high risk); and high risk of bias (as long as one or more parameters were rated as high risk)( Cochrane Handbook for Systematic Reviews of Interventions Table 8.7.a). Two reviewers ( C H and W S ) assessed each trial independently. Any disagreements were discussed with a third reviewer ( W G ) until a final decision was achieved.

**2.6. Patient and public involvement**

There was no direct patient or public involvement in this review.

**3. Results**

**3.1. Search results**

By searching electronic databases and literature, we obtained a total of 24,255 relevant references. After duplicates were removed, and titles and abstracts were checked against predefined inclusion criteria, 102 complete references were retained, and we reviewed each paper in detail. A total of 84 trials did not meet the predefined inclusion criteria: non-randomized controlled trials (n=4), no control group meeting the criteria (n=5), full text not available (n=6), assessment results not meeting the criteria (n=23), the study without results (n=9), data not available (n=13), intervention modality not meeting the pre-defined criteria (n=7), and study population not meeting the inclusion criteria (n=17). Therefore, a total of 18 articles were included in this study. The search process is shown in Figure 1.

**3.2 Research Characteristics**

A total of 18 randomized controlled trials were included in this study, published from 2011 to 2022. The characteristics of all included studies are summarized in Supplementary Table 1. The studies were conducted in different countries: China (n=8), the United States (n=2), Brazil (n=2), Japan (n=1), Korea (n=1), Turkey (n=1), Iran (n=1), Greece (n=1), and Spain (n=1). All included participants were 1700 and they were assigned to either the experimental or control group, and the sample size of the study ranged from 19-389. All participants were diagnosed with MCI. One study[14] was composed of the mind-body exercise intervention group and the conventional aerobic intervention group with their corresponding control group. Another study[15] was designed as two groups with different exercise intensities (40% and 60% reserve heart rate) for the conventional aerobic intervention. For all included studies, 5 experimental groups underwent multi-component exercise training with aerobic exercise[16-20]; 7 experimental groups underwent mind-body exercise training[14,21-26]; 8 experimental groups underwent conventional aerobic exercise training[14,15,27-31]. The total intervention duration for all studies ranged from 6 weeks to 12 months. The frequency was 21-80 min per session 1-5 times per week. Three experimental groups had an exercise intensity of roughly low intensity[14-16], i.e., maximum heart rate <60% or Borg scale (level 10) <5 or Borg scale (level 20) <12 or metabolic equivalent during exercise (MET) <3 or reserve heart rate <59%; 15 experimental groups had an exercise intensity of roughly moderate to high intensity[14,15,17-19,21-28,30,31], i.e., maximum heart rate ≥60% or Borg scale (10th grade) ≥5 or Borg scale (20th grade)) ≥ 12 or metabolic equivalent during exercise (MET) ≥ 3 or reserve heart rate ≥ 59%; the intensity of exercise in two other experimental groups was unclear[20,29].Ten studies assessed global cognitive function with the MMSE[15,17-20,25-27,30,31];5 studies assessed global cognitive function with the MoCA[14,21,23,28,29]; and 3 additional studies used both the MMSE and the MoCA[16,22,24].

**3.3. Literature quality evaluation**



For peer review only - http://bmjopen.bmj.com/site/about/guidelines.xhtml

As shown in Figure 2, the quality of studies included in the trials varied. Two trials had a low overall risk of bias[23,28], five trials had a moderate overall risk of bias[14,17,21,26,27], and eleven trials had a high overall risk of bias[15,16,18-20,22,24,25,29-31]. Regarding the method of randomization, 13 trials[14,16,17,19-24,28-31] had simple randomization and 5 trials[15,18,25-27] had stratified randomization. In terms of intervention allocation concealment, 9 trials[16,18-20,22,24,26,27,29] used open randomization tables, 3 trials[21,23,28] used sealed envelopes, and the remaining 6 trials[14,15,17,25,30,31] were unclear.

**3.4. Effects of aerobic exercises on global cognitive function**

We analyzed the trials in which the assessment results were MMSE and MoCA separately. The detailed analysis was then divided into multi-component aerobic exercise, mind-body exercise, and conventional aerobic exercise according to the exercise modality.

**3.4.1 The assessment with MMSE**

**3.4.1.1 The improvement of global cognitive function**

A total of 13 trials were included, and as shown in Figure 3, random-effects model meta-analysis revealed that aerobic exercise with MMSE outcomes was effective in improving global cognitive function compared to controls (MD=1.39, 95% CI [0.62, 2.17], p<0.01). Significant heterogeneity was found in the study ($I^2$=88%, p<0.01).

**3.4.1.2 The effect of various forms of exercise on global cognitive function**

All trials were divided into multi-component aerobic exercise, mind-body exercise, and conventional aerobic exercise by exercise modality, and the results of the meta-analysis was shown in Figure 4. As can be seen from the figure, after grouping by exercise modality, the difference between groups was significant ($I^2$=89.7%, p<0.01) indicating that the grouping was meaningful. Based on the results after grouping, it was clear that the conventional aerobic exercise intervention group had a significant improvement in global cognitive function (MD=0.51, 95% CI [0.09, 0.93], P<0.05) and a significant decrease in heterogeneity ($I^2$=0%, P=0.48); and the mind-body exercise intervention has also resulted in a significant improvement effect (MD=1.28, 95% CI [0.83 1.74], P<0.01) and a significant decrease in heterogeneity ($I^2$=50%, P=0.11); while multi-component aerobic exercise, despite also having a significant beneficial effect (MD=1.79, 95% CI [1.41, 2.17], P<0.01) had a very high heterogeneity ($I^2$=95%, P<0.01) and therefore needs further analysis. We performed sensitivity analyses and subgroup analyses on the multi-component aerobic exercise intervention group, and the results were shown in Tables 1 and 2. After sensitivity analyses, the removal of either study did not show any influence on the results obtained and heterogeneity remained almost unchanged. We performed subgroup analyses based on the sample size of the trial, exercise intensity, geographic region, risk assessment grading, duration, frequency, and total duration of the intervention. It was clear from the results that no meaningful results were obtained after subgroup analysis.

Table 1：The sensitivity analysis of Multi-component exercise (MMSE)

| Exclude studies | MD | 95% CI | $I^2$,% | Homogeneity Test | |
|---|---|---|---|---|---|
| | | | | Q | P |
| Bademli K 2019 | 0.99** | 0.55,1.43 | 90% | 30.76 | ＜0. 01 |
| de Oliveira2019 | 1.88)** | 1.48,2.27 | 96% | 80.32 | ＜0. 01 |
| Lam LCW 2015 | 3.11** | 2.60,3.63 | 88% | 25.06 | ＜0. 01 |
| 2019 Langoni | 1.72** | 1.33,2.11 | 96% | 78.37 | ＜0. 01 |
| Li L 2021 | 1.61** | 1.17,2.05 | 96% | 80.96 | ＜0. 01 |

** P＜0.01

Table 2：The subgroup analysis of Multi-component exercise (MMSE)

| Subgroups | | Number of studies | Number of participants | MD | 95% CI | $I^2$ | Q-test | $P^g$ |
|---|---|---|---|---|---|---|---|---|
| Sample size [a] | Small | 2 | 71 | 1.69* | 0. 30，3. 08 | 88% | ＜0.01 | =0. 88 |
| | Large | 3 | 422 | 1.80** | 1. 40，2. 20 | 97% | ＜0. 01 | |
| region | Asia | 3 | 422 | 1.80** | 1. 40，2. 20 | 97% | ＜0. 01 | =0. 88 |
| | Europe | 2 | 71 | 1.69* | 0. 30，3. 08 | 88% | ＜0. 01 | |
| Exercise intensity [b] | low | 1 | 84 | 2.37** | 1. 59，3. 15 | — | — | =0.01 |
| | Medium | 3 | 131 | 3.68** | 3. 00，4. 36 | 88% | ＜0. 01 | |



For peer review only - http://bmjopen.bmj.com/site/about/guidelines.xhtml

| | | | | | | | | |
|---|---|---|---|---|---|---|---|---|
| | to high | | | | | | | |
| Duration of intervention [c] | short | 1 | 84 | 2.37** | 1. 59，3. 15 | — | — | ＜0. 01 |
| | middle | 3 | 349 | 0.33 | -0. 20，0. 87 | 84% | ＜0. 01 | |
| | long | 1 | 60 | 4.30** | 3. 53，5. 08 | — | — | |
| Frequency of intervention [d] | middle | 3 | 349 | 0.33 | -0. 20，0. 87 | 84% | ＜0. 01 | ＜0. 01 |
| | high | 2 | 144 | 3.34** | 2. 79，3. 88 | 92% | ＜0. 01 | |
| Total intervention time [e] | middle | 4 | 409 | 1.61** | 1. 17，2. 05 | 96% | ＜0. 01 | =0.09 |
| | long | 1 | 84 | 2.37** | 1. 59，3. 15 | — | — | |
| Deviation risk rating [f] | middle | 1 | 52 | 4.60** | 2.20，7.00 | — | — | ＜0. 01 |
| | high | 4 | 441 | 1.72** | 1.33，2.11 | 96% | ＜0. 01 | |

[a]＜60=Small sample size；≥60=Large sample size，[b]Maximum heart rate <60% or Borg scale (10th grade) <5 or Borg scale (20th grade) < 12 or metabolic equivalent (MET) <3 or reserve heart rate <59% =Low strength; Maximum heart rate ≥ 60% or Borg scale (10th grade) ≥5 or Borg scale (20th grade) ≥ 12 or metabolic equivalent (MET) ≥3 or reserve heart rate ≥59% = medium to high intensity，[c]≤30 minutes = short；＞30 且≤60 minutes = middle； >60 minutes =long [d] 1 time/week = low; 2-3 times/week = middle； 4 times and more /week =high [e]＜3 months (12 weeks) = short； ≥ 3 months and < 6 months (12-23 weeks) = middle； ≥6 months (24 weeks) =long [f]All criteria are rated as low risk= Low risk of bias；Some standards are rated as unclear and not rated as high risk = medium deviation risk; As long as a standard is rated as high risk = high risk of deviation, [g]Differences between groups

* P＜0.05

** P＜0.01

For peer review only

### 3.4.2 The assessment with MoCA

#### 3.4.2.1 The improvement of global cognitive function

A total of 8 trials were included, and as shown in Figure 5, the random-effects model meta-analysis revealed that aerobic exercise with MoCA outcomes was effective in improving global cognitive function compared to controls (MD=2.62,95% CI [1.14, 4.10], p<0.01). Significant heterogeneity was found in the study (I²=94%, p<0.01).

#### 3.4.2.2 The effect of various forms of exercise on global cognitive function

Similarly, all trials with MoCA results were categorized by exercise modality into multi-component aerobic exercise, mind-body exercise, and conventional aerobic exercise. Meta-analysis results were shown in Figure 6. The significant difference between the groups with different exercise modality interventions (I²=97.6%, p<0.01) indicated that the effect of different exercise intervention groups was distinct. The cognitive function scores were significantly higher in the mind-body exercise intervention group than in the control group (MD=1.29,95% CI [0.67, 1.90], p<0.01) and less heterogeneous (I²=6%, p=0.36), which seemed to show that the improvement of the mind-body exercise was better. Although the results were also beneficial in the conventional aerobic exercise intervention group (MD=2.06,95% CI [1.46, 2.65], p<0.01), the greater heterogeneity (I²=84%, p<0.01) still needed further explanation. There was only one multi-component aerobic exercise trial with a positive MoCA outcome (MD=5.54, 95% CI [5.02, 6.46], p<0.01). Since there were only three trials in the conventional aerobic exercise intervention group, it was not convenient to subdivide them into subgroups. After sensitivity analysis on a case-by-case basis, as shown in Table 3, we found a small decrease in heterogeneity after excluding the article Tao J 2019[14] (I²=63%, p=0.10).

Table 3：The sensitivity analysis of conventional aerobic exercise (MoCA).

| Exclude studies | MD | 95% CI | I²,% | Homogeneity Test | |
|---|---|---|---|---|---|
| | | | | Q | P |
| Choi In 2018 | 1.75** | 1.10,2.40 | 86% | 6.96 | ＜0. 01 |
| Song D 2019 | 1.71** | 0.74,2.69 | 91% | 11.54 | ＜0. 01 |
| Tao J 2019② | 2.55** | 1.87,3.22 | 63% | 2.73 | =0. 10 |

** P < 0.01

## 4. Discussion

This meta-analysis explored the effects of different aerobic exercise types on global cognitive function in the elderly with MCI. The studies in this review were from different countries, including China. The overall quality of trials was variable, with most trials having a

moderate or high risk of bias. Our study analyzed improvements in cognitive function through endpoint indicators of global cognitive function and found that all three types of aerobic exercise above produced positive outcomes. This review may provide valuable evidence for future studies.

**4.1.Summary of the literature**

There were few previous meta-analyses[32,33] that explored the effects of multi-component exercise on cognitive function in MCI. Although some of their results suggested a beneficial effect of multi-component aerobic exercise on cognitive function in the elderly with MCI, the intervention criteria for the studies included in these meta-analyses were not only exercise but also cognitive training and mental stimulation, which were not the same as in our study. The Meta-analysis by Borges -Machado et al. [34] discussed multi-component exercise, which was defined as exercise that combined aerobic, strength, posture, and balance training. Although the study found improvements in ADL in the subjects, the study was conducted in patients with dementia and the results did not determine the effects on cognitive function.

Previous studies[35,36] confirmed the beneficial effects of mind-body exercise on global cognitive function in people with MCI. Unlike the present study, they had slight differences in the type of trial studies, age, and outcome evaluation indicators in the inclusion criteria. Our inclusion criteria were relatively more stringent. These previous studies combined different cognitive evaluation metrics expressed as standardized mean differences (SMD). We selected the two most common indicators of global cognitive function evaluation (MMSE and MoCA) to be analyzed separately and expressed the results as mean difference (MD).

We did not find any meta-analysis related to conventional aerobic exercise in patients with MCI. Although there are a few reviews[9,37,38] reporting aerobic exercise in MCI patients, they did not analyze the types of aerobic exercise separately. Therefore, we believed that the analysis of the present study was still of practical relevance.

**4.2. Findings of this study**

The results of our study demonstrated a significant effect of multi-component aerobic exercise, mind-body exercise, and conventional aerobic exercise on global cognitive function in older adults with MCI analyzed by both the MMSE and MoCA. However, there was high heterogeneity in the results of both the MMSE meta-analysis of multi-component aerobic exercise and the MoCA meta-analysis of conventional aerobic exercise. We focused on these points.

**4.3. Analysis of the results of mind-body exercise**

All studies of mind-body exercise reported positive results. The results of both the MMSE and MoCA meta-analyses were significant effects and low heterogeneity, which would seem to indicate the effectiveness of mind-body exercise in improving cognitive function in older adults with MCI. One possible explanation for the beneficial results of the mind-body exercise was that mind-body exercise was a form of physical exercise supplemented with different degrees of cognitive function training. For example, Tai Chi, a Chinese system of slow meditative physical exercise designed for mind relaxation and balance, required attention, memory, and other aspects in addition to physical activity. Adding cognitive training to physical activity seems to have better effects on cognitive function improvement than training alone[39]. Another possible explanation was that depression was an important risk factor for cognitive decline in people with MCI and that mind-body exercise improved mood in older adults better than other physical exercises[35].

**4.4. Heterogeneity analysis**

From a total of five studies analyzed by MMSE for multi-component aerobic exercise, two studies[18,20] were no meaningful change in outcomes. We analyzed these studies and speculated the possible reasons for such high heterogeneity. In the trial studied by de Oliveira et al.[18], the authors indicated that participants in both the trial and control groups were receiving medication, but no further medication-related descriptions. We are not sure if the medication affected the cognitive function of the subjects and thus the effect of the intervention. In another trial[20] that reported negative results, each exercise type of its multi-component aerobic exercise was performed separately, whereas common multi-component exercises combine different components in a single exercise session[34]. This was therefore the most different aspect of this study from other multi-component exercise training, and one possible explanation was that different components were more effective when combined in a single exercise session. One study showed[40] that short-term tai chi training did not significantly improve cognitive function in adults when comparing long-term and short-term tai chi training. In another trial[16], the duration of aerobic exercise was too short at only 5 minutes. Thus we speculated that there may be some difference in the results produced by the different times of aerobic exercise.

Conventional aerobic exercise is more common than multi-component aerobic exercise and mind-body exercise. The meta-analysis of conventional aerobic exercise in MoCA had high heterogeneity. It was well known that various physical conditions deteriorate with age in the elderly population, and the risk of progression to dementia in MCI patients increases accordingly[41]. Therefore, we speculated that the older the MCI patients were, the less beneficial the intervention would be. Some of the study[29] subjects were residents of the same community and were more female (80%), which may have made the study less representative. A review[42] had shown that older women have a higher degree of cognitive deterioration than men, which seemed to be possibly related to estrogen. In addition to these possible reasons, problems such as small sample sizes, poor subject compliance, and investigator implementation bias that occurred in some studies[14,18-20] may also have contributed to the high heterogeneity.

**4.5. Limitations**

There were several limitations of this review. One was that there was considerable heterogeneity between some of the included studies, representing both clinical and methodological heterogeneity, due to differences between participants (For example, older adults may have unknown comorbidities and the possibility of taking medications as a result, which may affect the effectiveness of the intervention), interventions, and study designs. The second was that it could not rule out that certain control groups would also be of varying degrees of benefit to cognitive function, such as social activities[43]. Third, only some of the trials that assessed global cognitive outcomes were included, and not those that assessed separately cognitive domains. Fourth, only studies of trials with MMSE and MoCA



For peer review only - http://bmjopen.bmj.com/site/about/guidelines.xhtml

outcomes were selected. Although these two cognitive evaluation metrics are the most commonly used, they also undoubtedly excluded other studies that might be of value.

### 4.6. Implications for future research

Different types of aerobic exercise have different experimental interventions. The duration, frequency, and intensity of the exercise intervention may have different degrees of influence on the results of the trial. Follow-up research protocols could focus on this to explore the design of better interventions. Follow-up surveys at the end of the trial are also important to demonstrate the long-term effects of the trial. The exercise components of multi-component aerobic training are complex and future studies could investigate better combinations of exercise components to achieve better improvements.

In addition, previous studies have indicated that the MoCA is superior to the MMSE for screening diagnoses in MCI patients, making it more recommended to replace the MMSE with the MoCA for future scales measuring global cognitive function[44]. in addition to the MMSE and the MoCA, the CDR (Clinical Dementia Rating) is a worthwhile scale to refer to and use. In addition to the global cognitive function, it would be interesting to assess specific domains of cognitive function (e.g., memory, attention), which may allow the inclusion of additional studies and better delineate the sources of heterogeneity involved.

## 5. Conclusion

The results of this meta-analysis suggest that all types of aerobic exercise (including multi-component aerobic exercise, mind-body exercise, and conventional aerobic exercise) were positive for improving global cognitive function in older adults with MCI. However, the conclusion should be treated cautiously due to the heterogeneity and limited research. Additional well-designed RCTs are needed to provide more evidence for this conclusion.

**Contributors:** All authors made a substantial contribution to this work. Conglin Han, Weishuang Sun, Dan Zhang, Xiaoshuang Xi, Rong Zhang and Weijun Gong all contributed to the conception and design of the review. Conglin Han drafted the paper with all the authors critically reviewing it and suggesting amendments prior to submission. All authors had full access to all of the data (including statistical reports and tables) in the study and can take responsibility for the integrity of the data and the accuracy of the data analysis.

**Competing interests:** There are no competing interests for any author.

**Funding:** This work was supported by Capital's Funds for Health Improvement and Research(2022-1-2251), Natural Science Foundation of China (81972148) and Beijing Natural Science Foundation of China (7222101).

**Data sharing statement:** All data relevant to the study are included in the article or uploaded as supplementary information.

**Ethics statements**

Patient consent for publication

Not applicable.

**Ethics approval**

This study does not directly involve human participants. Therefore, it is not applicable.

Figure 1: Study flow diagram.

Figure 2: Risk of bias assessment of included trials.

Figure 3: Effect sizes of forest plots for aerobic exercise versus controls-MMSE outcomes. MMSE, mini-mental state examination.

Figure 4: Effect sizes of forest plots for the different aerobic exercise-MMSE outcomes. MMSE, mini-mental state examination.

Figure 5: Effect sizes of forest plots for aerobic exercise versus controls-MoCA outcome. MoCA, Montreal Cognitive Assessment.

Figure 6: Effect sizes of forest plots for the different aerobic exercise-MoCA outcomes. MoCA, Montreal Cognitive Assessment

For peer review only



For peer review only - http://bmjopen.bmj.com/site/about/guidelines.xhtml

## References

[1] The Lancet Public H. Reinvigorating the public health response to dementia[J]. Lancet Public Health, 2021, 6(10): e696.

[2] WHO Guidelines Approved by the Guidelines Review Committee, Risk Reduction of Cognitive Decline and Dementia: WHO Guidelines, Geneva: World Health Organization © World Health Organization 2019., 2019.

[3] Sanford A M. Mild Cognitive Impairment[J]. Clin Geriatr Med, 2017, 33(3): 325-337.

[4] Jongsiriyanyong S, Limpawattana P. Mild Cognitive Impairment in Clinical Practice: A Review Article[J]. Am J Alzheimers Dis Other Demen, 2018, 33(8): 500-507.

[5] Schneider L S, Mangialasche F, Andreasen N, et al. Clinical trials and late-stage drug development for Alzheimer's disease: an appraisal from 1984 to 2014[J]. J Intern Med, 2014, 275(3): 251-83.

[6] Eshkoor S A, Hamid T A, Mun C Y, et al. Mild cognitive impairment and its management in older people[J]. Clin Interv Aging, 2015, 10: 687-93.

[7] Law C K, Lam F M, Chung R C, et al. Physical exercise attenuates cognitive decline and reduces behavioural problems in people with mild cognitive impairment and dementia: a systematic review[J]. J Physiother, 2020, 66(1): 9-18.

[8] Ahlskog J E, Geda Y E, Graff-Radford N R, et al. Physical exercise as a preventive or disease-modifying treatment of dementia and brain aging[J]. Mayo Clin Proc, 2011, 86(9): 876-84.

[9] Yong L, Liu L, Ding T, et al. Evidence of Effect of Aerobic Exercise on Cognitive Intervention in Older Adults With Mild Cognitive Impairment[J]. Front Psychiatry, 2021, 12: 713671.



For peer review only - http://bmjopen.bmj.com/site/about/guidelines.xhtml

[10] Li Z, Liu S, Wang L, et al. Mind-Body Exercise for Anxiety and Depression in COPD Patients: A Systematic Review and Meta-Analysis[J]. Int J Environ Res Public Health, 2019, 17(1).

[11] Zhang T, Liu W, Gao S. Effects of mind-body exercises on cognitive impairment in people with Parkinson's disease: A mini-review[J]. Front Neurol, 2022, 13: 931460.

[12] Wu C, Yi Q, Zheng X, et al. Effects of Mind-Body Exercises on Cognitive Function in Older Adults: A Meta-Analysis[J]. J Am Geriatr Soc, 2019, 67(4): 749-758.

[13] Higgins J P, Altman D G, Gøtzsche P C, et al. The Cochrane Collaboration's tool for assessing risk of bias in randomised trials[J]. Bmj, 2011, 343: d5928.

[14] Tao J, Liu J, Chen X, et al. Mind-body exercise improves cognitive function and modulates the function and structure of the hippocampus and anterior cingulate cortex in patients with mild cognitive impairment[J]. Neuroimage. Clinical, 2019, 23: 101834.

[15] Varela S, Ayán C, Cancela J M, et al. Effects of two different intensities of aerobic exercise on elderly people with mild cognitive impairment: a randomized pilot study[J]. Clin Rehabil, 2012, 26(5): 442-50.

[16] Li L, Liu M, Zeng H, et al. Multi-component exercise training improves the physical and cognitive function of the elderly with mild cognitive impairment: a six-month randomized controlled trial[J]. Annals of palliative medicine, 2021, 10(8): 8919-8929.

[17] Langoni C D S, Resende T L, Barcellos A B, et al. Effect of Exercise on Cognition, Conditioning, Muscle Endurance, and Balance in Older Adults With Mild Cognitive Impairment: a Randomized Controlled Trial[J]. Journal of geriatric physical therapy (2001), 2019, 42(2): E15-E22.



For peer review only - http://bmjopen.bmj.com/site/about/guidelines.xhtml

[18] De Oliveira Silva F, Ferreira J V, Plácido J, et al. Three months of multimodal training contributes to mobility and executive function in elderly individuals with mild cognitive impairment, but not in those with Alzheimer's disease: a randomized controlled trial[J]. Maturitas, 2019, 126: 28-33.

[19] Bademli K, Lok N, Canbaz M, et al. Effects of Physical Activity Program on cognitive function and sleep quality in elderly with mild cognitive impairment: a randomized controlled trial[J]. Perspectives in psychiatric care, 2019, 55(3): 401-408.

[20] Lam L C, Chan W C, Leung T, et al. Would older adults with mild cognitive impairment adhere to and benefit from a structured lifestyle activity intervention to enhance cognition?: a cluster randomized controlled trial[J]. PloS one, 2015, 10(3): e0118173.

[21] Li F, Harmer P, Fitzgerald K, et al. A cognitively enhanced online Tai Ji Quan training intervention for community-dwelling older adults with mild cognitive impairment: a feasibility trial[J]. BMC geriatrics, 2022, 22(1): 76.

[22] Qi M, Zhu Y I, Zhang L, et al. The effect of aerobic dance intervention on brain spontaneous activity in older adults with mild cognitive impairment: A resting-state functional MRI study[J]. Experimental and Therapeutic Medicine, 2019, 17(1): 715-722.

[23] Zhu Y, Wu H, Qi M, et al. Effects of a specially designed aerobic dance routine on mild cognitive impairment[J]. Clinical interventions in aging, 2018, 13: 1691-1700.

[24] Lazarou I, Parastatidis T, Tsolaki A, et al. International Ballroom Dancing Against Neurodegeneration: a Randomized Controlled Trial in Greek Community-Dwelling Elders With Mild Cognitive impairment[J]. American journal of Alzheimer's disease and other dementias, 2017, 32(8): 489-499.



For peer review only - http://bmjopen.bmj.com/site/about/guidelines.xhtml

[25] Doi T, Verghese J, Makizako H, et al. Effects of Cognitive Leisure Activity on Cognition in Mild Cognitive Impairment: Results of a Randomized Controlled Trial[J]. Journal of the American Medical Directors Association, 2017, 18(8): 686-691.

[26] Lam L C, Chan W M, Kwok T C, et al. Effectiveness of Tai Chi in maintenance of cognitive and functional abilities in mild cognitive impairment: a randomised controlled trial[J]. Hong Kong Med J, 2014, 20(3 Suppl 3): 20-3.

[27] Tomoto T, Liu J, Tseng B Y, et al. One-Year Aerobic Exercise Reduced Carotid Arterial Stiffness and Increased Cerebral Blood Flow in Amnestic Mild Cognitive Impairment[J]. Journal of Alzheimer's disease, 2021, 80(2): 841-853.

[28] Song D, Yu D S F. Effects of a moderate-intensity aerobic exercise programme on the cognitive function and quality of life of community-dwelling elderly people with mild cognitive impairment: a randomised controlled trial[J]. International journal of nursing studies, 2019, 93: 97-105.

[29] Choi W, Lee S. Ground Kayak Paddling Exercise Improves Postural Balance, Muscle Performance, and Cognitive Function in Older Adults with Mild Cognitive Impairment: a Randomized Controlled Trial[J]. Medical science monitor, 2018, 24: 3909-3915.

[30] Kohanpour M-A, Mohammad-Ali K, Maghsoud P, et al. The effects of aerobic exercise with lavender essence use on cognitive state and serum brain-derived neurotrophic factor levels in elderly with mild cognitive impairment[J]. Journal of herbmed pharmacology, 2017, 6(2): 80-84.

[31] Wei X H, Ji L L. Effect of handball training on cognitive ability in elderly with mild cognitive impairment[J]. Neuroscience letters, 2014, 566: 98-101.

[32] Özbe D, Graessel E, Donath C, et al. Immediate Intervention Effects of Standardized



For peer review only - http://bmjopen.bmj.com/site/about/guidelines.xhtml

Multicomponent Group Interventions on People with Cognitive Impairment: A Systematic Review[J]. J Alzheimers Dis, 2019, 67(2): 653-670.

[33] Bruderer-Hofstetter M, Rausch-Osthoff A K, Meichtry A, et al. Effective multicomponent interventions in comparison to active control and no interventions on physical capacity, cognitive function and instrumental activities of daily living in elderly people with and without mild impaired cognition - A systematic review and network meta-analysis[J]. Ageing Res Rev, 2018, 45: 1-14.

[34] Borges-Machado F, Silva N, Farinatti P, et al. Effectiveness of Multicomponent Exercise Interventions in Older Adults With Dementia: A Meta-Analysis[J]. Gerontologist, 2021, 61(8): e449-e462.

[35] Wang S, Yin H, Jia Y, et al. Effects of Mind-Body Exercise on Cognitive Function in Older Adults With Cognitive Impairment: A Systematic Review and Meta-analysis[J]. J Nerv Ment Dis, 2018, 206(12): 913-924.

[36] Zou L, Loprinzi P D, Yeung A S, et al. The Beneficial Effects of Mind-Body Exercises for People With Mild Cognitive Impairment: a Systematic Review With Meta-analysis[J]. Arch Phys Med Rehabil, 2019, 100(8): 1556-1573.

[37] Cammisuli D M, Innocenti A, Franzoni F, et al. Aerobic exercise effects upon cognition in Mild Cognitive Impairment: A systematic review of randomized controlled trials[J]. Arch Ital Biol, 2017, 155(1-2): 54-62.

[38] Zheng G, Xia R, Zhou W, et al. Aerobic exercise ameliorates cognitive function in older adults with mild cognitive impairment: a systematic review and meta-analysis of randomised controlled trials[J]. Br J Sports Med, 2016, 50(23): 1443-1450.



For peer review only - http://bmjopen.bmj.com/site/about/guidelines.xhtml

[39] Gheysen F, Poppe L, Desmet A, et al. Physical activity to improve cognition in older adults: can physical activity programs enriched with cognitive challenges enhance the effects? A systematic review and meta-analysis[J]. Int J Behav Nutr Phys Act, 2018, 15(1): 63.

[40] Walsh J N, Manor B, Hausdorff J, et al. Impact of Short- and Long-term Tai Chi Mind-Body Exercise Training on Cognitive Function in Healthy Adults: Results From a Hybrid Observational Study and Randomized Trial[J]. Glob Adv Health Med, 2015, 4(4): 38-48.

[41] Petersen R C, Lopez O, Armstrong M J, et al. Practice guideline update summary: Mild cognitive impairment: Report of the Guideline Development, Dissemination, and Implementation Subcommittee of the American Academy of Neurology[J]. Neurology, 2018, 90(3): 126-135.

[42] Alchalabi T, Prather C. Brain Health[J]. Clin Geriatr Med, 2021, 37(4): 593-604.

[43] James B D, Wilson R S, Barnes L L, et al. Late-life social activity and cognitive decline in old age[J]. J Int Neuropsychol Soc, 2011, 17(6): 998-1005.

[44] Pinto T C C, Machado L, Bulgacov T M, et al. Is the Montreal Cognitive Assessment (MoCA) screening superior to the Mini-Mental State Examination (MMSE) in the detection of mild cognitive impairment (MCI) and Alzheimer's Disease (AD) in the elderly?[J]. Int Psychogeriatr, 2019, 31(4): 491-504.



For peer review only - http://bmjopen.bmj.com/site/about/guidelines.xhtml

**Provenance and peer review:** Not commissioned; externally peer reviewed.

I [Conglin Han], the Submitting Author has the right to grant and does grant on behalf of all authors of the Work (as defined in the below author licence), an exclusive licence and/or a non-exclusive licence for contributions from authors who are: i) UK Crown employees; ii) where BMJ has agreed a CC BY licence shall apply, and/or iii) in accordance with the terms applicable for US Federal Government officers or employees acting as part of their official duties; on a worldwide, perpetual, irrevocable, royalty-free basis to BMJ Publishing Group Ltd (“BMJ”) its licensees and where the relevant Journal is co-owned by BMJ to the co-owners of the Journal, to publish the Work in [JOURNAL NAME] and any other BMJ products and to exploit all rights, as set out in our licence [link]

For peer review only



For peer review only - http://bmjopen.bmj.com/site/about/guidelines.xhtml

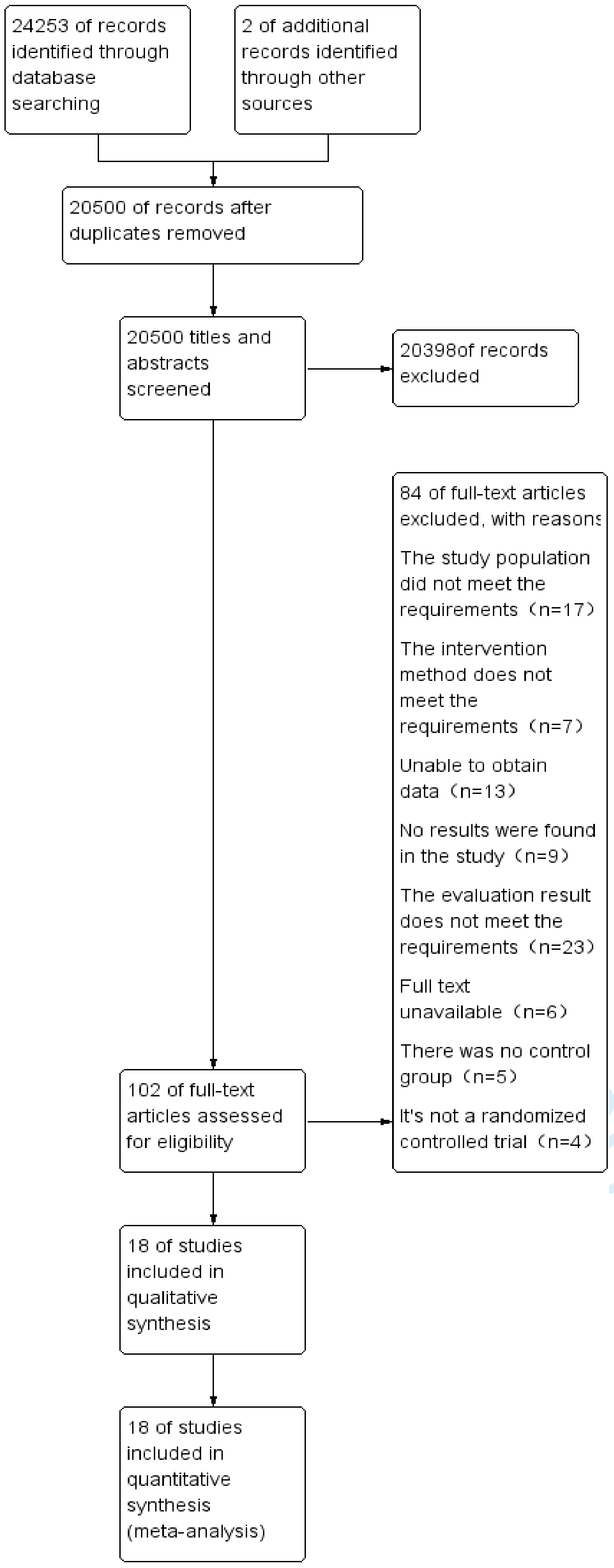


Figure 1： Study flow diagram.

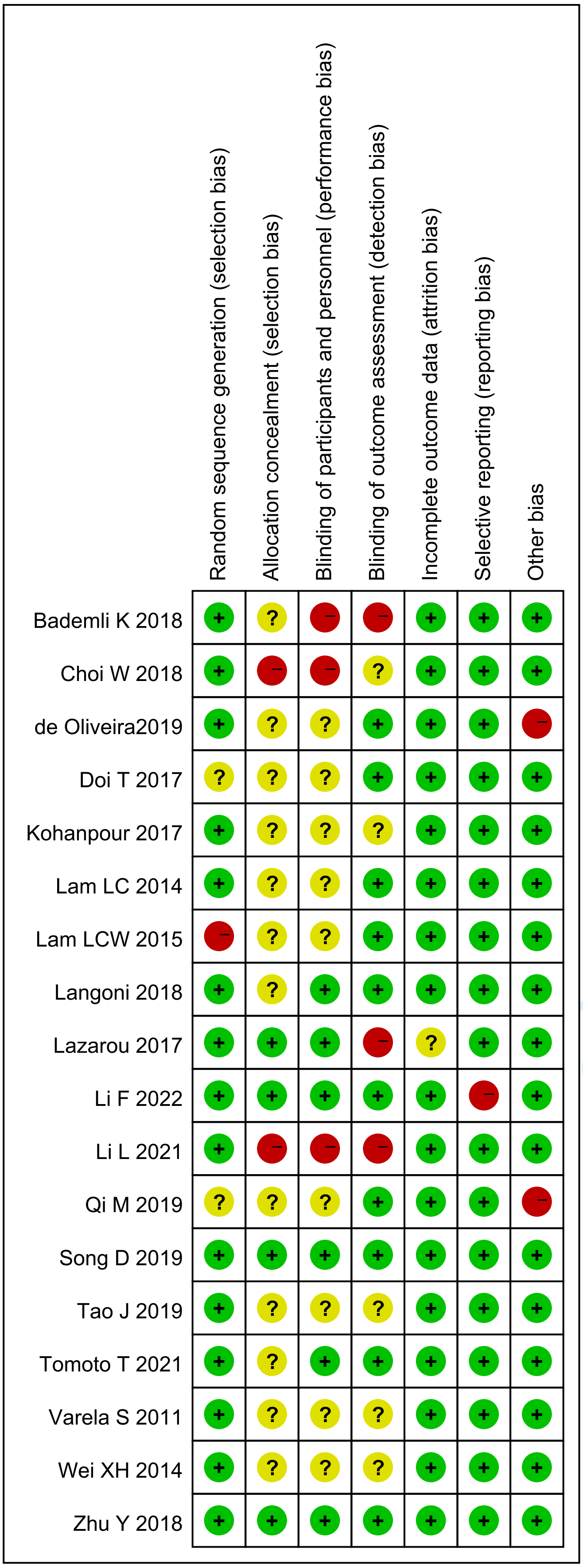


Figure 2: Risk of bias assessment of included trials.

| | Experimental | | | Control | | | | Mean Difference | Mean Difference |
|---|---|---|---|---|---|---|---|---|---|
| Study or Subgroup | Mean | SD | Total | Mean | SD | Total | Weight | IV, Fixed, 95% CI | IV, Fixed, 95% CI |
| Bademli K 2019 | 26.542 | 1.84 | 30 | 22.24 | 1.15 | 30 | 9.6% | 4.30 [3.53, 5.08] | |
| de Oliveira2019 | 28.25 | 0.92 | 12 | 28.04 | 2.2 | 7 | 2.0% | 0.21 [-1.50, 1.92] | |
| Doi T 2017 | 26.29 | 2.6 | 55 | 25.44 | 2.18 | 63 | 7.6% | 0.85 [-0.02, 1.72] | |
| Kohanpour 2017 | 24.4 | 1.42 | 10 | 24.2 | 0.63 | 10 | 6.2% | 0.20 [-0.76, 1.16] | |
| Lam LC 2014 | 25.4 | 3.3 | 96 | 24.2 | 3.4 | 169 | 8.3% | 1.20 [0.36, 2.04] | |
| Lam LCW 2015 | 25.6 | 2.4 | 147 | 25.5 | 2.5 | 131 | 17.3% | 0.10 [-0.48, 0.68] | |
| Langoni 2019 | 25 | 4.7 | 26 | 20.4 | 4.1 | 26 | 1.0% | 4.60 [2.20, 7.00] | |
| Lazarou 2017 | 28 | 2.39 | 66 | 25.65 | 3.27 | 63 | 5.9% | 2.35 [1.36, 3.34] | |
| Li L 2021 | 27.79 | 1.18 | 42 | 25.42 | 2.28 | 42 | 9.6% | 2.37 [1.59, 3.15] | |
| Qi M 2019 | 28.2 | 1 | 16 | 27.3 | 1.7 | 16 | 6.2% | 0.90 [-0.07, 1.87] | |
| Tomoto T 2021 | 28.5 | 1.46 | 22 | 28.5 | 1.5 | 30 | 8.8% | 0.00 [-0.81, 0.81] | |
| Varela S 2012① | 20.66 | 7.39 | 17 | 19.53 | 5.5 | 15 | 0.3% | 1.13 [-3.35, 5.61] | |
| Varela S 2012② | 21.06 | 5.4 | 16 | 19.53 | 5.5 | 15 | 0.4% | 1.53 [-2.31, 5.37] | |
| Wei XH 2014 | 25.53 | 0.82 | 30 | 24.67 | 1.42 | 30 | 16.8% | 0.86 [0.27, 1.45] | |
| **Total (95% CI)** | | | **585** | | | **647** | **100.0%** | **1.23 [0.99, 1.47]** | |

Heterogeneity: Chi² = 112.89, df = 13 (P < 0.00001); I² = 88%
Test for overall effect: Z = 10.04 (P < 0.00001)

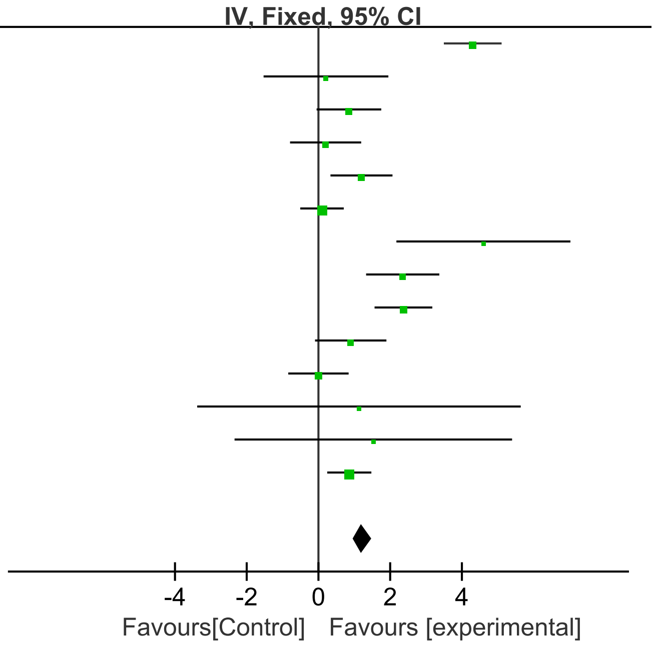


Figure 3: Effect sizes of forest plots for aerobic exercise versus control-MMSE outcomes. MMSE, mini-mental state examination

For peer review only

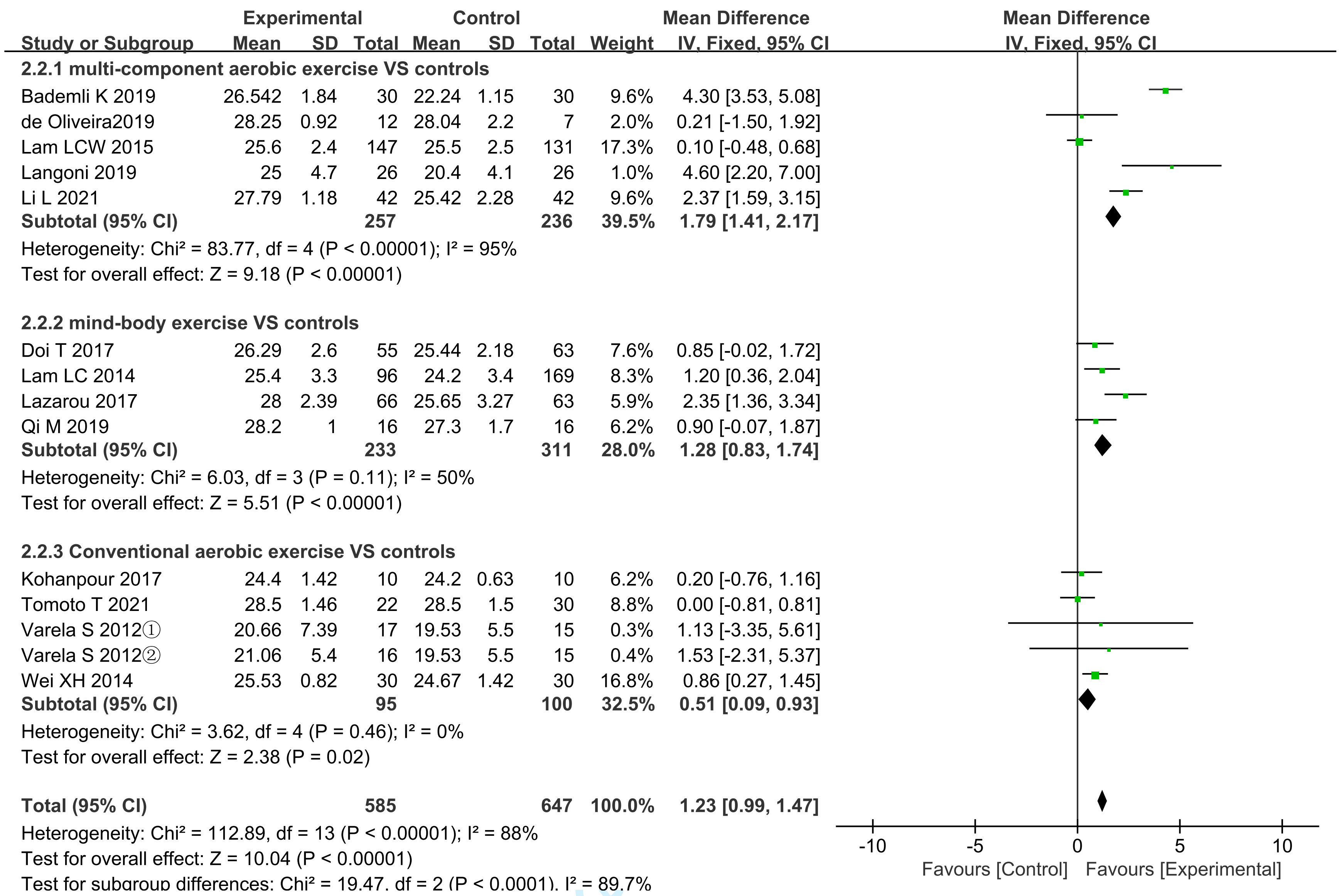


Figure 4: Effect sizes of forest plots for the different aerobic exercise-MMSE outcomes. MMSE, mini-mental state examination.

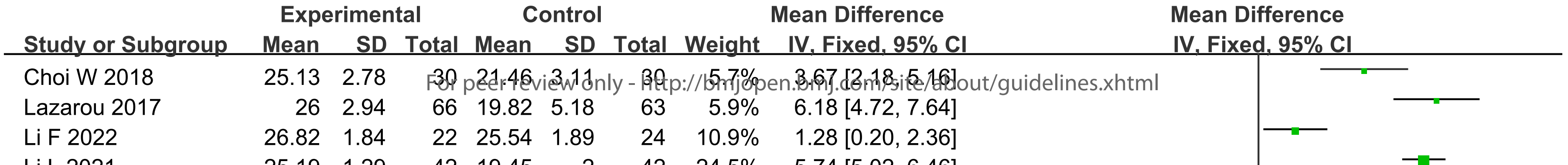

| Study or Subgroup | Experimental Mean | Experimental SD | Experimental Total | Control Mean | Control SD | Control Total | Weight | Mean Difference IV, Fixed, 95% CI |
|---|---|---|---|---|---|---|---|---|
| Choi W 2018 | 25.13 | 2.78 | 30 | 21.46 | 3.11 | 30 | 5.7% | 3.67 [2.18, 5.16] |
| Lazarou 2017 | 26 | 2.94 | 66 | 19.82 | 5.18 | 63 | 5.9% | 6.18 [4.72, 7.64] |
| Li F 2022 | 26.82 | 1.84 | 22 | 25.54 | 1.89 | 24 | 10.9% | 1.28 [0.20, 2.36] |
| Li L 2021 | 25.19 | 1.29 | 42 | 19.45 | 2 | 42 | 24.5% | 5.74 [5.02, 6.46] |
| Qi M 2019 | 24.3 | 2.2 | 16 | 23.7 | 2 | 16 | 6.0% | 0.60 [-0.86, 2.06] |
| Song D 2019 | 23.66 | 1.92 | 60 | 21.4 | 2.27 | 60 | 22.4% | 2.26 [1.51, 3.01] |
| Tao J 2019① | 24.55 | 2.33 | 20 | 22.1 | 2.59 | 20 | 5.4% | 2.45 [0.92, 3.98] |
| Tao J 2019② | 22.35 | 1.29 | 17 | 22.1 | 2.59 | 20 | 7.6% | 0.25 [-1.04, 1.54] |
| Zhu Y 2018 | 24.7 | 2.2 | 27 | 23.6 | 1.8 | 31 | 11.6% | 1.10 [0.06, 2.14] |
| **Total (95% CI)** | | | **300** | | | **306** | **100.0%** | **2.94 [2.58, 3.30]** |

Heterogeneity: Chi² = 129.08, df = 8 (P < 0.00001); I² = 94%

Test for overall effect: Z = 16.19 (P < 0.00001)

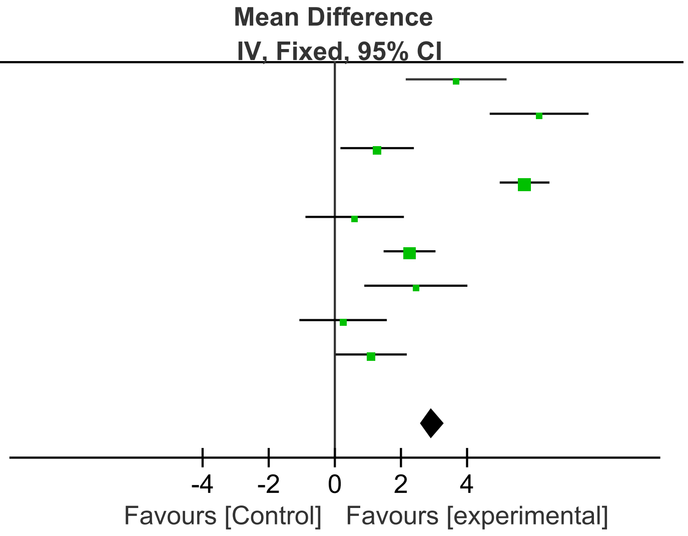


Figure 5: Effect sizes of forest plots for aerobic exercise versus controls-MoCA outcome. MoCA, Montreal Cognitive Assessment.

For peer review only

| Study or Subgroup | Experimental Mean | Experimental SD | Experimental Total | Control Mean | Control SD | Control Total | Weight | Mean Difference IV, Fixed, 95% CI |
|---|---|---|---|---|---|---|---|---|
| **3.2.1 multi-component aerobic exercise VS controls** | | | | | | | | |
| Li L 2021 | 25.19 | 1.29 | 42 | 19.45 | 2 | 42 | 26.0% | 5.74 [5.02, 6.46] |
| **Subtotal (95% CI)** | | | **42** | | | **42** | **26.0%** | **5.74 [5.02, 6.46]** |
| Heterogeneity: Not applicable | | | | | | | | |
| Test for overall effect: Z = 15.63 (P < 0.00001) | | | | | | | | |
| **3.2.2 mind-body exercise VS controls** | | | | | | | | |
| Li F 2022 | 26.82 | 1.84 | 22 | 25.54 | 1.89 | 24 | 11.6% | 1.28 [0.20, 2.36] |
| Qi M 2019 | 24.3 | 2.2 | 16 | 23.7 | 2 | 16 | 6.3% | 0.60 [-0.86, 2.06] |
| Tao J 2019① | 24.55 | 2.33 | 20 | 22.1 | 2.59 | 20 | 5.8% | 2.45 [0.92, 3.98] |
| Zhu Y 2018 | 24.7 | 2.2 | 27 | 23.6 | 1.8 | 31 | 12.4% | 1.10 [0.06, 2.14] |
| **Subtotal (95% CI)** | | | **85** | | | **91** | **36.1%** | **1.29 [0.67, 1.90]** |
| Heterogeneity: Chi² = 3.21, df = 3 (P = 0.36); I² = 6% | | | | | | | | |
| Test for overall effect: Z = 4.12 (P < 0.0001) | | | | | | | | |
| **3.2.3 Conventional aerobic exercise VS controls** | | | | | | | | |
| Choi W 2018 | 25.13 | 2.78 | 30 | 21.46 | 3.11 | 30 | 6.0% | 3.67 [2.18, 5.16] |
| Song D 2019 | 23.66 | 1.92 | 60 | 21.4 | 2.27 | 60 | 23.8% | 2.26 [1.51, 3.01] |
| Tao J 2019② | 22.35 | 1.29 | 17 | 22.1 | 2.59 | 20 | 8.1% | 0.25 [-1.04, 1.54] |
| **Subtotal (95% CI)** | | | **107** | | | **110** | **37.9%** | **2.06 [1.46, 2.65]** |
| Heterogeneity: Chi² = 12.30, df = 2 (P = 0.002); I² = 84% | | | | | | | | |
| Test for overall effect: Z = 6.76 (P < 0.00001) | | | | | | | | |
| **Total (95% CI)** | | | **234** | | | **243** | **100.0%** | **2.74 [2.37, 3.10]** |
| Heterogeneity: Chi² = 109.05, df = 7 (P < 0.00001); I² = 94% | | | | | | | | |
| Test for overall effect: Z = 14.61 (P < 0.00001) | | | | | | | | |
| Test for subgroup differences: Chi² = 93.54, df = 2 (P < 0.00001), I² = 97.9% | | | | | | | | |

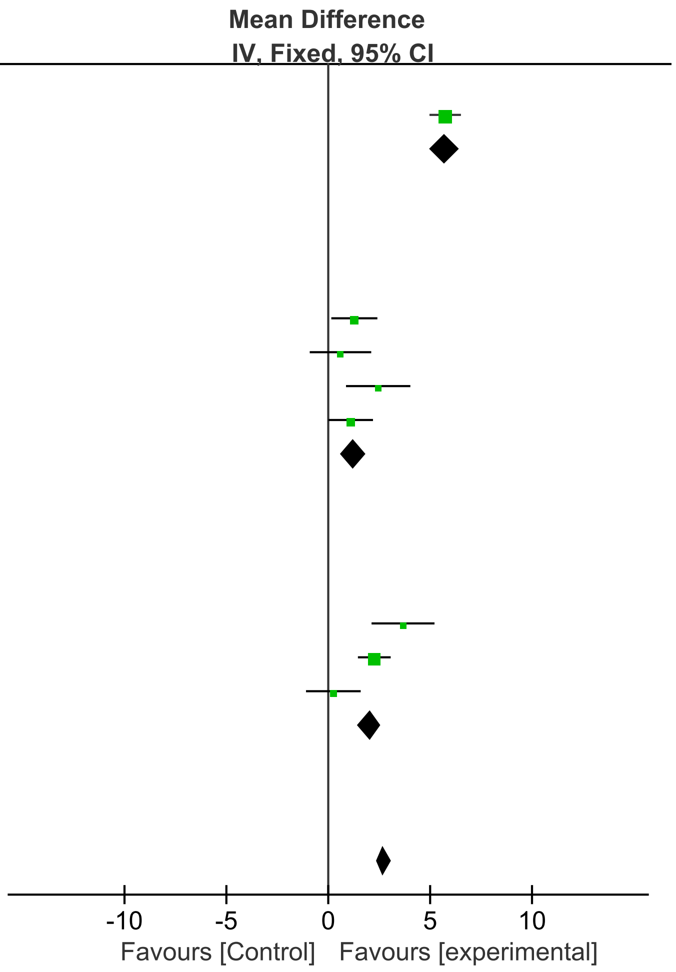


Figure 6: Effect sizes of forest plots for the different aerobic exercise-MoCA outcomes. MoCA, Montreal Cognitive Assessment

For peer review only

**Embase Search strategy**

| Search number | Search Details | Results |
|---|---|---|
| 1# | 'aerobic exercise'/exp | 19335 |
| 2# | 'aerobic dance':ab,ti OR 'aerobic dancing':ab,ti OR aerobics:ab,ti OR 'aerobics exercise':ab,ti OR 'dancing, aerobic':ab,ti OR 'exercise, aerobic':ab,ti OR 'low impact aerobic exercise':ab,ti OR 'low impact aerobics':ab,ti OR 'step aerobics':ab,ti | 1495 |
| 3# | 'aerobic exercise'/exp OR ('aerobic dance':ab,ti OR 'aerobic dancing':ab,ti OR aerobics:ab,ti OR 'aerobics exercise':ab,ti OR 'dancing, aerobic':ab,ti OR 'exercise, aerobic':ab,ti OR 'low impact aerobic exercise':ab,ti OR 'low impact aerobics':ab,ti OR 'step aerobics':ab,ti) | 20255 |
| 4# | 'exercise'/exp | 399161 |
| 5# | 'biometric exercise':ab,ti OR effort:ab,ti OR 'exercise capacity':ab,ti OR 'exercise performance':ab,ti OR 'exercise training':ab,ti OR exertion:ab,ti OR 'fitness training':ab,ti OR 'fitness workout':ab,ti OR 'physical conditioning':ab,ti OR human:ab,ti OR 'physical effort':ab,ti OR 'physical exercise':ab,ti OR 'physical exertion':ab,ti OR 'physical work-out':ab,ti OR 'physical workout':ab,ti | 3576566 |
| 6# | 'exercise'/exp OR ('biometric exercise':ab,ti OR effort:ab,ti OR 'exercise capacity':ab,ti OR 'exercise performance':ab,ti OR 'exercise training':ab,ti OR exertion:ab,ti OR 'fitness training':ab,ti OR 'fitness workout':ab,ti OR 'physical conditioning':ab,ti OR human:ab,ti OR 'physical effort':ab,ti OR 'physical exercise':ab,ti OR 'physical exertion':ab,ti OR 'physical work-out':ab,ti OR 'physical workout':ab,ti) | 3884161 |
| 7# | 'physical activity'/exp | 485896 |
| 8# | 'activity, physical':ab,ti | 1355 |
| 9# | 'physical activity'/exp OR 'activity, physical':ab,ti | 486507 |
| 10# | 'cognitive defect'/exp | 557350 |
| 11# | 'cognition disorders':ab,ti OR 'cognitive defects':ab,ti OR 'cognitive deficit':ab,ti OR 'cognitive disability':ab,ti OR 'cognitive disorder':ab,ti OR 'cognitive disorders':ab,ti OR 'cognitive dysfunction':ab,ti OR 'cognitive impairment':ab,ti OR 'delirium, dementia, amnestic, cognitive disorders':ab,ti OR overinclusion:ab,ti OR 'response interference':ab,ti | 136389 |
| 12# | 'cognitive defect'/exp OR ('cognition disorders':ab,ti OR 'cognitive defects':ab,ti OR 'cognitive deficit':ab,ti OR 'cognitive disability':ab,ti OR 'cognitive | 577692 |

For peer review only

| | | |
|---|---|---|
| | disorder':ab,ti OR 'cognitive disorders':ab,ti OR 'cognitive dysfunction':ab,ti OR 'cognitive impairment':ab,ti OR 'delirium, dementia, amnestic, cognitive disorders':ab,ti OR overinclusion:ab,ti OR 'response interference':ab,ti) | |
| 13# | 'mild cognitive impairment'/exp | 32022 |
| 14# | 'amnestic mild cognitive impairment':ab,ti | 3430 |
| 15 | 'mild cognitive impairment'/exp OR 'amnestic mild cognitive impairment':ab,ti | 32561 |
| 16# | ('aerobic exercise'/exp OR ('aerobic dance':ab,ti OR 'aerobic dancing':ab,ti OR aerobics:ab,ti OR 'aerobics exercise':ab,ti OR 'dancing, aerobic':ab,ti OR 'exercise, aerobic':ab,ti OR 'low impact aerobic exercise':ab,ti OR 'low impact aerobics':ab,ti OR 'step aerobics':ab,ti)) OR ('exercise'/exp OR ('biometric exercise':ab,ti OR effort:ab,ti OR 'exercise capacity':ab,ti OR 'exercise performance':ab,ti OR 'exercise training':ab,ti OR exertion:ab,ti OR 'fitness training':ab,ti OR 'fitness workout':ab,ti OR 'physical conditioning':ab,ti OR human:ab,ti OR 'physical effort':ab,ti OR 'physical exercise':ab,ti OR 'physical exertion':ab,ti OR 'physical work-out':ab,ti OR 'physical workout':ab,ti)) OR ('physical activity'/exp OR 'activity, physical':ab,ti) | 4258734 |
| 17# | ('cognitive defect'/exp OR ('cognition disorders':ab,ti OR 'cognitive defects':ab,ti OR 'cognitive deficit':ab,ti OR 'cognitive disability':ab,ti OR 'cognitive disorder':ab,ti OR 'cognitive disorders':ab,ti OR 'cognitive dysfunction':ab,ti OR 'cognitive impairment':ab,ti OR 'delirium, dementia, amnestic, cognitive disorders':ab,ti OR overinclusion:ab,ti OR 'response interference':ab,ti)) OR ('mild cognitive impairment'/exp OR 'amnestic mild cognitive impairment':ab,ti) | 577692 |
| 18# | (('aerobic exercise'/exp OR ('aerobic dance':ab,ti OR 'aerobic dancing':ab,ti OR aerobics:ab,ti OR 'aerobics exercise':ab,ti OR 'dancing, aerobic':ab,ti OR 'exercise, aerobic':ab,ti OR 'low impact aerobic exercise':ab,ti OR 'low impact aerobics':ab,ti OR 'step aerobics':ab,ti)) OR ('exercise'/exp OR ('biometric exercise':ab,ti OR effort:ab,ti OR 'exercise capacity':ab,ti OR 'exercise performance':ab,ti OR 'exercise training':ab,ti OR exertion:ab,ti OR 'fitness training':ab,ti OR 'fitness workout':ab,ti OR 'physical conditioning':ab,ti OR human:ab,ti OR 'physical effort':ab,ti OR 'physical exercise':ab,ti OR 'physical exertion':ab,ti OR 'physical work-out':ab,ti OR 'physical workout':ab,ti)) OR ('physical activity'/exp OR 'activity, physical':ab,ti)) AND (('cognitive defect'/exp OR ('cognition disorders':ab,ti OR 'cognitive defects':ab,ti OR 'cognitive deficit':ab,ti OR 'cognitive disability':ab,ti OR 'cognitive disorder':ab,ti OR 'cognitive disorders':ab,ti OR 'cognitive | 76351 |

For peer review only

| | | |
|---|---|---|
| | dysfunction':ab,ti OR 'cognitive impairment':ab,ti OR 'delirium, dementia, amnestic, cognitive disorders':ab,ti OR overinclusion:ab,ti OR 'response interference':ab,ti)) OR ('mild cognitive impairment'/exp OR 'amnestic mild cognitive impairment':ab,ti)) | |
| 19# | 'aged'/exp | 3469661 |
| 20# | 'aged patient':ab,ti OR 'aged people':ab,ti OR 'aged person':ab,ti OR 'aged subject':ab,ti OR elderly:ab,ti OR 'elderly patient':ab,ti OR 'elderly people':ab,ti OR 'elderly person':ab,ti OR 'elderly subject':ab,ti OR 'senior citizen':ab,ti OR senium:ab,ti | 385314 |
| 21# | 'aged'/exp OR ('aged patient':ab,ti OR 'aged people':ab,ti OR 'aged person':ab,ti OR 'aged subject':ab,ti OR elderly:ab,ti OR 'elderly patient':ab,ti OR 'elderly people':ab,ti OR 'elderly person':ab,ti OR 'elderly subject':ab,ti OR 'senior citizen':ab,ti OR senium:ab,ti) | 3555832 |
| 22# | ((('aerobic exercise'/exp OR ('aerobic dance':ab,ti OR 'aerobic dancing':ab,ti OR aerobics:ab,ti OR 'aerobics exercise':ab,ti OR 'dancing, aerobic':ab,ti OR 'exercise, aerobic':ab,ti OR 'low impact aerobic exercise':ab,ti OR 'low impact aerobics':ab,ti OR 'step aerobics':ab,ti)) OR ('exercise'/exp OR ('biometric exercise':ab,ti OR effort:ab,ti OR 'exercise capacity':ab,ti OR 'exercise performance':ab,ti OR 'exercise training':ab,ti OR exertion:ab,ti OR 'fitness training':ab,ti OR 'fitness workout':ab,ti OR 'physical conditioning':ab,ti OR human:ab,ti OR 'physical effort':ab,ti OR 'physical exercise':ab,ti OR 'physical exertion':ab,ti OR 'physical work-out':ab,ti OR 'physical workout':ab,ti)) OR ('physical activity'/exp OR 'activity, physical':ab,ti)) AND (('cognitive defect'/exp OR ('cognition disorders':ab,ti OR 'cognitive defects':ab,ti OR 'cognitive deficit':ab,ti OR 'cognitive disability':ab,ti OR 'cognitive disorder':ab,ti OR 'cognitive disorders':ab,ti OR 'cognitive dysfunction':ab,ti OR 'cognitive impairment':ab,ti OR 'delirium, dementia, amnestic, cognitive disorders':ab,ti OR overinclusion:ab,ti OR 'response interference':ab,ti)) OR ('mild cognitive impairment'/exp OR 'amnestic mild cognitive impairment':ab,ti))) AND ('aged'/exp OR ('aged patient':ab,ti OR 'aged people':ab,ti OR 'aged person':ab,ti OR 'aged subject':ab,ti OR elderly:ab,ti OR 'elderly patient':ab,ti OR 'elderly people':ab,ti OR 'elderly person':ab,ti OR 'elderly subject':ab,ti OR 'senior citizen':ab,ti OR senium:ab,ti)) | 16100 |

For peer review only

**Pubmed Search strategy**

| Search number | Search Details | Results |
|---|---|---|
| 1# | "Cognitive Dysfunction"[Mesh] | 228,279 |
| 2# | ((((((((((((((Exercises[Title/Abstract]) OR (Physical Activity[Title/Abstract])) OR (Physical Activities[Title/Abstract])) OR (Activities, Physical[Title/Abstract])) OR (Activity, Physical[Title/Abstract])) OR (Exercise*, Physical[Title/Abstract])) OR (Physical Exercise*[Title/Abstract])) OR (Acute Exercise*[Title/Abstract])) OR (Exercise*, Acute[Title/Abstract])) OR (Exercise*, Isometric[Title/Abstract])) OR (Isometric Exercise*[Title/Abstract])) OR (Exercise*, Aerobic[Title/Abstract])) OR (Aerobic Exercise*[Title/Abstract])) OR (Exercise Training*[Title/Abstract])) OR (Training*, Exercise[Title/Abstract]) | 293,650 |
| 3# | ("Exercise"[Mesh]) OR ((((((((((((((Exercises[Title/Abstract]) OR (Physical Activity[Title/Abstract])) OR (Physical Activities[Title/Abstract])) OR (Activities, Physical[Title/Abstract])) OR (Activity, Physical[Title/Abstract])) OR (Exercise*, Physical[Title/Abstract])) OR (Physical Exercise*[Title/Abstract])) OR (Acute Exercise*[Title/Abstract])) OR (Exercise*, Acute[Title/Abstract])) OR (Exercise*, Isometric[Title/Abstract])) OR (Isometric Exercise*[Title/Abstract])) OR (Exercise*, Aerobic[Title/Abstract])) OR (Aerobic Exercise*[Title/Abstract])) OR (Exercise Training*[Title/Abstract])) OR (Training*, Exercise[Title/Abstract])) | 409,704 |
| 4# | "Cognitive Dysfunction"[Mesh] | 29,147 |
| 5# | ((((((((((((Cognitive Dysfunctions[Title/Abstract]) OR (Dysfunction*, Cognitive[Title/Abstract])) OR (Cognitive Impairment*[Title/Abstract])) OR (Impairment*, Cognitive[Title/Abstract])) OR (Mild Cognitive Impairment*[Title/Abstract])) OR (Cognitive Impairment*, Mild[Title/Abstract])) OR (Impairment*, Mild Cognitive[Title/Abstract])) OR (Mild Neurocognitive Disorder*[Title/Abstract])) OR (Disorder*, Mild Neurocognitive[Title/Abstract])) OR (Neurocognitive Disorder*, Mild[Title/Abstract])) OR (Cognitive Decline*[Title/Abstract])) OR (Decline*, Cognitive[Title/Abstract])) OR (Mental Deterioration*[Title/Abstract])) OR (Deterioration*, Mental[Title/Abstract]) | 169,309 |
| 6# | ("Cognitive Dysfunction"[Mesh]) OR (((((((((((((Cognitive Dysfunctions[Title/Abstract]) OR (Dysfunction*, Cognitive[Title/Abstract])) OR (Cognitive Impairment*[Title/Abstract])) OR (Impairment*, Cognitive[Title/Abstract])) OR (Mild Cognitive Impairment*[Title/Abstract])) OR (Cognitive Impairment*, Mild[Title/Abstract])) OR (Impairment*, Mild Cognitive[Title/Abstract])) OR (Mild Neurocognitive Disorder*[Title/Abstract])) OR (Disorder*, Mild Neurocognitive[Title/Abstract])) OR (Neurocognitive Disorder*, Mild[Title/Abstract])) OR (Cognitive Decline*[Title/Abstract])) OR (Decline*, Cognitive[Title/Abstract])) OR (Mental Deterioration*[Title/Abstract])) OR (Deterioration*, Mental[Title/Abstract])) | 171,419 |
| 7# | (("Exercise"[Mesh]) OR ((((((((((((((Exercises[Title/Abstract]) OR (Physical Activity[Title/Abstract])) OR (Physical Activities[Title/Abstract])) OR (Activities, Physical[Title/Abstract])) OR (Activity, Physical[Title/Abstract])) OR (Exercise*, Physical[Title/Abstract])) OR (Physical | 6,686 |

Exercise*[Title/Abstract])) OR (Acute Exercise*[Title/Abstract])) OR (Exercise*, Acute[Title/Abstract])) OR (Exercise*, Isometric[Title/Abstract])) OR (Isometric Exercise*[Title/Abstract])) OR (Exercise*, Aerobic[Title/Abstract])) OR (Aerobic Exercise*[Title/Abstract])) OR (Exercise Training*[Title/Abstract])) OR (Training*, Exercise[Title/Abstract]))) AND (("Cognitive Dysfunction"[Mesh]) OR ((((((((((((((Cognitive Dysfunctions[Title/Abstract]) OR (Dysfunction*, Cognitive[Title/Abstract])) OR (Cognitive Impairment*[Title/Abstract])) OR (Impairment*, Cognitive[Title/Abstract])) OR (Mild Cognitive Impairment*[Title/Abstract])) OR (Cognitive Impairment*, Mild[Title/Abstract])) OR (Impairment*, Mild Cognitive[Title/Abstract])) OR (Mild Neurocognitive Disorder*[Title/Abstract])) OR (Disorder*, Mild Neurocognitive[Title/Abstract])) OR (Neurocognitive Disorder*, Mild[Title/Abstract])) OR (Cognitive Decline*[Title/Abstract])) OR (Decline*, Cognitive[Title/Abstract])) OR (Mental Deterioration*[Title/Abstract])) OR (Deterioration*, Mental[Title/Abstract])))

For peer review only

**Cochrane Library Search strategy**

| Search number | Search Details | Results |
|---|---|---|
| 1# | MeSH descriptor: [Exercise] explode all trees | 32772 |
| 2# | (Exercise*, Acute):ti,ab,kw OR (Acute Exercise*):ti,ab,kw OR (Training*, Exercise):ti,ab,kw OR (Exercise Training*):ti,ab,kw OR (Exercise*, Isometric):ti,ab,kw (Word variations have been searched) | 52015 |
| 3# | (Exercise*, Aerobic):ti,ab,kw OR (Aerobic Exercise*):ti,ab,kw OR (Physical Exercise*):ti,ab,kw OR (Physical Activity):ti,ab,kw OR (Activity, Physical):ti,ab,kw (Word variations have been searched) | 97132 |
| 4# | (Activities, Physical):ti,ab,kw OR (Exercise*, Physical):ti,ab,kw OR (Physical Activitie):ti,ab,kw (Word variations have been searched) | 68139 |
| 5# | #2 OR #3 OR #4 | 12098 |
| 6# | #1 OR #5 | 130117 |
| 7# | MeSH descriptor: [Cognitive Dysfunction] explode all trees | 2809 |
| 8# | (Impairment*, Mild Cognitive):ti,ab,kw OR (Disorder*, Mild Neurocognitive):ti,ab,kw OR (Mild Neurocognitive Disorder*):ti,ab,kw OR (Mild Cognitive Impairment*):ti,ab,kw OR (Neurocognitive Disorder*, Mild):ti,ab,kw (Word variations have been searched) | 5434 |
| 9# | (Cognitive Impairment*, Mild):ti,ab,kw OR (Dysfunction*, Cognitive):ti,ab,kw OR (Impairment*, Cognitive):ti,ab,kw OR (Cognitive Dysfunctions):ti,ab,kw OR (Cognitive Impairment*):ti,ab,kw (Word variations have been searched) | 23085 |
| 10# | (Mental Deterioration*):ti,ab,kw OR (Decline*, Cognitive):ti,ab,kw OR (Deterioration*, Mental;):ti,ab,kw OR (Cognitive Decline*):ti,ab,kw (Word variations have been searched) | 8024 |
| 11# | #7 OR #8 OR #9 OR #10 | 28106 |
| 12# | #6 AND #11 | 4714 |
| 13# | MeSH descriptor: [Aged] explode all trees | 242328 |
| 14# | (Elderly):ti,ab,kw (Word variations have been searched) | 57985 |
| 15# | #13 OR #14 | 283159 |
| 16# | #12 AND #15 | 1492 |

Supplementary Table 1： Baseline characteristics of included trials

| Aerobic exercise type | study | region | | Participants | | | intervene | | | | |
|---|---|---|---|---|---|---|---|---|---|---|---|
| | | | | Sample size | age | female | manner | frequency | strength | MMSE | MoCA |
| multi-component aerobic exercise | Li, L (2021)[12] | Asia/ China | Experimental group | 42 | 60–69（47.6%）、<br>70–79（47.6%）、<br>80 and above（4.8%） | 64% | 5 minutes warm-up, 5 minutes aerobic training, 5 minutes strength training, 5 minutes balance training, 5 minutes coordination training, 5 minutes sensitivity training | 30 minutes × 5 times× 24 weeks (6 months). | The warm-up is a 3-4/10 Perceptual Nu (RPE) level, and the rest of the workout is 4–5/10 RPE | 26.50±1.33 | 21.52±2.05 |
| | | | Control group | 42 | 60–69（42.9%）、<br>70–79（50.0%）、<br>80 and above（7.1%） | 57% | General community health guidance | | | 26.62±1.46 | 21.14±1.97 |
| | Bademli (2019)[15] | Asian/ Turkey | Experimental group | 30 | 72.24± 7.16 | 60% | 10 minutes of warm-up, 20 minutes of rhythmic exercise (including 22 simple exercises), 10 minutes of cooling exercise (low-intensity exercise), 40 minutes of free walking. | 80 minutes ×20 weeks, 4/3/7 days per week | 3-6 metabolic equivalents (ME). | 23.27 ± 2.17 | |
| | | | Control group | 30 | 70.67 ± 8.34 | 57% | Continue with your daily activities | | | 23.42 ± 1.07 | |
| | De Oliveira (2019)[14] | Euro-American/ Brazil | Experimental group | 7 | 71.85 ± 5.69 | 85.71% | 5 minutes of balance training,30 minutes of aerobic training, 20 minutes of strength training, 5 minutes of stretching exercises | 60 minutes × 2 times × 12 weeks | The intensity of the main training is 70% of the maximum oxygen consumption (VO2max) or 80% of the predicted maximum heart rate (HRmax). | 29 ±0.61 | |
| | | | Control group | 12 | 78.20± 5.26 | 41.66% | Clinical follow-up without any physical training | | | 27.8 ±2.57 | |
| | Langoni (2019)[13] | Euro-American/ Brazil | Experimental group | 26 | 72.6 ±7.8 | 76.9% | Strength training takes about30 minutes (with equipment including ankle weight, resistance latex tape and dumbbells) and aerobic training (20-30 minutes of walking). | 60 minutes× 2 times × 24 weeks | Aerobic training to walk at a maximum heart rate of 60% to 75%. | 21.9 (4.8) | |
| | | | Control group | 26 | 71.9 ±7.9 | 76.9% | Do not initiate any form of physical or cognitive activity and keep in touch by phone | | | 25.0 (4.7) | |
| | Lam (2015)[16] | Asia/ China | Experimental group | 147 | 75.5±6.7 | 76.9% | Stretching and conditioning exercises, mind-body exercises (such as tai chi) and aerobic exercises (such as static cycling). | 120 minutes× 3×48 weeks (12 months). | Unknown | 25.8(2.3) | |

For peer review only

| | | | | | | | | | | | |
|---|---|---|---|---|---|---|---|---|---|---|---|
| | | | Control group | 131 | 75.4±6.1 | 77.9% | Social activities (e.g., tea parties, movie watching). | | | 25.6(2.4) | |
| mind-body exercise | Li,F (2022) | Euro-America/ United States | Experimental group | 22 | 74.5 ±5.6 | 36.4% | Traditional Tai Chi training | 60 minutes× 2 times × 16 weeks | The metabolic equivalent of Tai Chi exercise is 3.5-4.3 MET | | 25.09±2.43 |
| | | | Control group | 24 | 74.9±6.3 | 62.5% | Stretching exercises | | | | 25.13±2.19 |
| | Tao,J (2019)[10] ① | Asia/ China | Experimental group | 20 | 66.17±4.17 | 75% | 15 minutes to warm up, 40 minutes to eight danjin training and 5 minutes to cool down | 60 minutes × 3 times × 24 weeks | The Batdan Nishiki Movement MET is 2.68 | | 22.45(2.16) |
| | | | Control group | 20 | 65.97±5.66 | 70% | Maintain their original level of physical activity, do not receive any specific exercise interventions, health education | | | | 21.00(2.36) |
| | Qi,M (2019)[18] | Asia/ China | Experimental group | 16 | 70.6±6.2 | 69% | 35 minutes of moderate intensity aerobic dance (5 minutes of warm-up, 25 minutes of target heart rate dance and 5 minutes of music cooling). | 35 minutes × 3 times× 12 weeks (three months). | 60-80% maximum heart rate as the target heart rate for aerobic dance | 27.3 (1.3) | 22.6 (2.1) |
| | | | Control group | 16 | 69.1±8.1 | 75% | Routine care | | | 27.1 (1.2) | 22.9 (1.7) |
| | Zhu,Y (2018)[19] | Asia/ China | Experimental group | 29 | 70.3±6.7 | 51.7% | 5 minutes of warm-up, 25 minutes of target heart rate dance and 5 minutes of cooling | 35 minutes × 3 times× 12 weeks (three months | The target heart rate is set at 60%-80% of your maximum heart rate | | 23.2 (1.9) |
| | | | Control group | 31 | 69.0±7.3 | 67.7% | Routine care | | | | 22.9 (2.1) |
| | Lazarou, (2017)[20] | Euro-American/ Greece | Experimental group | 66 | 65.89± 10.76 | 80% | International Ballroom Dance (5 minutes of warm-up, review of previous dance lessons, 45 minutes of new material (tricks/dances), and 10 minutes of cooling time). | 60 minutes× 2 times× 40 weeks (10 months). | Dancing About 6 METs | 27.60 2.19 | 24.70 2.25 |
| | | | Control group | 33 | 67.92±9.47 | 76% | No intervention was accepted, the educational meeting was received | | | 26.88 2.1 | 23.81 3.1 |
| | Doi,T (2017)[21] | Asia/ Japan | Experimental group | 67 | 75.7±4.1 | 50.7% | The dance program is based on ballroom dances, including salsa, rumba, waltz, cha-cha, bruce, kitba and tango | 60 minutes × once × 40 weeks | Dancing About 6 METs | 26.0 (2.6) | |
| | | | Control group | 67 | 76.0±4.9 | 46.3% | Health education class | | | 25.8 (2.4) | |
| | Lam (2014)[22] | Asia/ China | Experimental group | 171 | 77.2±6.3 | 73% | 24 Styles of Tai Chi " Training. | During the introductory phase, the coaching process is 4 to 6 weeks. | The metabolic equivalent of Tai Chi exercise is 3.5-4.3 MET | 25.1±3.0 | |

For peer review only

| | | | | | | | | | | | |
|---|---|---|---|---|---|---|---|---|---|---|---|
| | | | | | | | | During the maintenance phase, the frequency of interventions was ≥ 30 minutes per day and ≥ 3 days a week. Until the 1stmonth of the 2nd month | | | |
| | | | Control group | 218 | 78.3±6.6 | 79% | Stretching and relaxation exercises | | | 24.4±2.9 | |
| Conventional aerobic exercise | Tomoto, (2021)[23] | Euro-American /USA | Experimental group | 22 | 64.8 ± 6.4 | 55% | Treadmill running | 1-10 weeks: 25-30 minutes × 3 times. 11-25 weeks: 30-35minutes× 3 or 4 times.26 weeks - 1 year: 30-40 minutes× 4-5 times | The intensity is 75%-85% of the maximum heart rate (HR). | 29.0 ± 1.5 | |
| | | | Control group | 30 | 66. 1 ± 6.8 | 53% | Stretching and conditioning interventions | | | 28.8 ± 1.4 | |
| | Song,D (2019)[24] | Asia/ China | Experimental group | 60 | 76.22 ±5.76 | 80% | Aerobic tread exercises | 60 minutes× 3 times × 16 weeks | For moderate-intensity workouts using the Borg Scale, the Borg score is 12-14 | | 22.03 ? 1.81 |
| | | | Control group | 60 | 75.33 ±6.78 | 70% | Health education class | | | | 22.10 ? 1.92 |
| | Tao,J (2019)[10] ② | Asia/ China | Experimental group | 17 | 64.32±2.60 | 59% | Warm up for 15 minutes, walk fast for 40 minutes, cool down for 5 minutes. | 60 minutes × 3 times × 24 weeks | The participants' heart rate was controlled at 55%-75% of the reserve heart rate | | 21.47(2.27) |
| | | | Control group | 20 | 65.97±5.6 | 70% | Maintain the original level of physical activity and do not receive any specific exercise interventions, health education | | | | 21.00(2.36) |
| | Choi,W (2018)[25] | Asia/ South Korea | Experimental group | 30 | 74.90±5.10 | 80% | 10min warm-up activity, 40min GKP exercise and 10min cooling activity. | 60 minutes× 2 times × 6 weeks | Unknown | | 21.66 (3.24 |
| | | | Control group | 30 | 74.23±4.38 | 83% | Stretching, warm-up and cooling exercises are the same as in the exercise group | | | | 20.76 (3.02) |
| | Kohanpour (2017)[26] | Asia/ Iran | Experimental group | 10 | 60-70 | 0% | 8min warm-up, (8-26)min running and cooling down for 5min | 21-39 minutes × 3 times × 12 weeks | The intensity is 75% to 85% of the maximum heart rate | 22.70±1.63 | |
| | | | Control group | 10 | 60-70 | 0% | Habitual lifestyle | | | 24.30±0.94 | |
| | Wei, X H (2014)[27] | Asia/ China | Experimental group | 30 | 66.73±5.48 | 30% | Handball training | 30 minutes × 5 times × 6 months (120 times). | The average intensity of aerobic exercise is about 60% of your maximum heart rate | 24.33(1.65) | |
| | | | Control group | 30 | 65.27±4.63 | 36.7% | Maintain the original way of life and entertainment | | | 25.00(1.29) | |

For peer review only

| | | | | | | | | | |
|---|---|---|---|---|---|---|---|---|---|
| Varela, S (2012)[11] ① | Euro-American/ Spain | Experimental group | 17 | 79.24± 10.07 | Unknown | 5 minutes of warm-up, 20 minutes of recumbent bike ride, and 5 minutes of stretching to cool down | 30 minutes × 3 times × 12 weeks | Exercise intensity is set at 40% of the participant's reserve heart rate (maximum heart rate - resting heart rate). | 19.86 ? 5.12 |
| | | Control group | 15 | 79.40 ±6.72 | | Social entertainment | | | 21.80 ? 3.23 |
| Varela, S (2012)[11] ② | Euro-American/ Spain | Experimental group | 16 | 76.44 ±11.38 | Unknown | 5 minutes of warm-up, 20 minutes of recumbent bike ride, and 5 minutes of stretching to cool down | 30 minutes × 3 times × 12 weeks | The exercise intensity is set at 60% of the participant's reserve heart rate (maximum heart rate - resting heart rate). | 20.81 ? 4.69 |
| | | Control group | 15 | 79.40 ±6.72 | | Social entertainment | | | 21.80 ? 3.23 |

For peer review only

# PRISMA 2020 Checklist

| Section and Topic | Item # | Checklist item | Location where item is reported |
|---|---|---|---|
| **TITLE** | | | |
| Title | 1 | Identify the report as a systematic review. | Page 1 |
| **ABSTRACT** | | | |
| Abstract | 2 | See the PRISMA 2020 for Abstracts checklist. | Page 2 |
| **INTRODUCTION** | | | |
| Rationale | 3 | Describe the rationale for the review in the context of existing knowledge. | Page 2 |
| Objectives | 4 | Provide an explicit statement of the objective(s) or question(s) the review addresses. | Page 1 |
| **METHODS** | | | |
| Eligibility criteria | 5 | Specify the inclusion and exclusion criteria for the review and how studies were grouped for the syntheses. | Page 2 |
| Information sources | 6 | Specify all databases, registers, websites, organisations, reference lists and other sources searched or consulted to identify studies. Specify the date when each source was last searched or consulted. | Page 2 and figure |
| Search strategy | 7 | Present the full search strategies for all databases, registers and websites, including any filters and limits used. | supplementary statement |
| Selection process | 8 | Specify the methods used to decide whether a study met the inclusion criteria of the review, including how many reviewers screened each record and each report retrieved, whether they worked independently, and if applicable, details of automation tools used in the process. | Page 2 |
| Data collection process | 9 | Specify the methods used to collect data from reports, including how many reviewers collected data from each report, whether they worked independently, any processes for obtaining or confirming data from study investigators, and if applicable, details of automation tools used in the process. | Page 3 |
| Data items | 10a | List and define all outcomes for which data were sought. Specify whether all results that were compatible with each outcome domain in each study were sought (e.g. for all measures, time points, analyses), and if not, the methods used to decide which results to collect. | Page 3 |
| | 10b | List and define all other variables for which data were sought (e.g. participant and intervention characteristics, funding sources). Describe any assumptions made about any missing or unclear information. | Page 2 |
| Study risk of bias assessment | 11 | Specify the methods used to assess risk of bias in the included studies, including details of the tool(s) used, how many reviewers assessed each study and whether they worked independently, and if applicable, details of automation tools used in the process. | Page 3 |
| Effect measures | 12 | Specify for each outcome the effect measure(s) (e.g. risk ratio, mean difference) used in the synthesis or presentation of results. | Page 3 |
| Synthesis methods | 13a | Describe the processes used to decide which studies were eligible for each synthesis (e.g. tabulating the study intervention characteristics and comparing against the planned groups for each synthesis (item #5)). | Page 2 |
| | 13b | Describe any methods required to prepare the data for presentation or synthesis, such as handling of missing summary statistics, or data conversions. | Page 3 |
| | 13c | Describe any methods used to tabulate or visually display results of individual studies and syntheses. | Page 3 |
| | 13d | Describe any methods used to synthesize results and provide a rationale for the choice(s). If meta-analysis was performed, describe the model(s), method(s) to identify the presence and extent of statistical heterogeneity, and software package(s) used. | Page 3 |
| | 13e | Describe any methods used to explore possible causes of heterogeneity among study results (e.g. subgroup analysis, meta-regression). | Page 3 |
| | 13f | Describe any sensitivity analyses conducted to assess robustness of the synthesized results. | Page 3 |
| Reporting bias assessment | 14 | Describe any methods used to assess risk of bias due to missing results in a synthesis (arising from reporting biases). | -( Does not meet the number of |

For peer review only - http://bmjopen.bmj.com/site/about/guidelines.xhtml

# PRISMA 2020 Checklist

| Section and Topic | Item # | Checklist item | Location where item is reported |
|---|---|---|---|
| | | | studies required for funnel plots) |
| Certainty assessment | 15 | Describe any methods used to assess certainty (or confidence) in the body of evidence for an outcome. | Page 8 |
| **RESULTS** | | | |
| Study selection | 16a | Describe the results of the search and selection process, from the number of records identified in the search to the number of studies included in the review, ideally using a flow diagram. | Figure 1 |
| | 16b | Cite studies that might appear to meet the inclusion criteria, but which were excluded, and explain why they were excluded. | Figure 1 |
| Study characteristics | 17 | Cite each included study and present its characteristics. | Table 2 |
| Risk of bias in studies | 18 | Present assessments of risk of bias for each included study. | Figure 2 |
| Results of individual studies | 19 | For all outcomes, present, for each study: (a) summary statistics for each group (where appropriate) and (b) an effect estimate and its precision (e.g. confidence/credible interval), ideally using structured tables or plots. | All figure |
| Results of syntheses | 20a | For each synthesis, briefly summarise the characteristics and risk of bias among contributing studies. | Page 8 |
| | 20b | Present results of all statistical syntheses conducted. If meta-analysis was done, present for each the summary estimate and its precision (e.g. confidence/credible interval) and measures of statistical heterogeneity. If comparing groups, describe the direction of the effect. | Figure 3-6<br>Page 8-10 |
| | 20c | Present results of all investigations of possible causes of heterogeneity among study results. | Page 8<br>Figure 2 |
| | 20d | Present results of all sensitivity analyses conducted to assess the robustness of the synthesized results. | Figure 11 |
| Reporting biases | 21 | Present assessments of risk of bias due to missing results (arising from reporting biases) for each synthesis assessed. | - |
| Certainty of evidence | 22 | Present assessments of certainty (or confidence) in the body of evidence for each outcome assessed. | Table 4 |
| **DISCUSSION** | | | |
| Discussion | 23a | Provide a general interpretation of the results in the context of other evidence. | Page 10 |
| | 23b | Discuss any limitations of the evidence included in the review. | Page 11 |
| | 23c | Discuss any limitations of the review processes used. | Page 11 |
| | 23d | Discuss implications of the results for practice, policy, and future research. | Page 11 |
| **OTHER INFORMATION** | | | |
| Registration and protocol | 24a | Provide registration information for the review, including register name and registration number, or state that the review was not registered. | Page 2 |
| | 24b | Indicate where the review protocol can be accessed, or state that a protocol was not prepared. | - |
| | 24c | Describe and explain any amendments to information provided at registration or in the protocol. | -( Not modified) |
| Support | 25 | Describe sources of financial or non-financial support for the review, and the role of the funders or sponsors in the review. | Page 15 |
| Competing | 26 | Declare any competing interests of review authors. | - |

For peer review only

For peer review only - http://bmjopen.bmj.com/site/about/guidelines.xhtml

## PRISMA 2020 Checklist

| Section and Topic | Item # | Checklist item | Location where item is reported |
|---|---|---|---|
| interests | | | |
| Availability of data, code and other materials | 27 | Report which of the following are publicly available and where they can be found: template data collection forms; data extracted from included studies; data used for all analyses; analytic code; any other materials used in the review. | Page 2 and Page 3 |

*From:* Page MJ, McKenzie JE, Bossuyt PM, Boutron I, Hoffmann TC, Mulrow CD, et al. The PRISMA 2020 statement: an updated guideline for reporting systematic reviews. BMJ 2021;372:n71. doi: 10.1136/bmj.n71

For more information, visit: http://www.prisma-statement.org/

For peer review only

BMJ Open

# Effects of different aerobic exercises on the global cognitive function of the elderly with mild cognitive impairment: a meta-analysis

| | |
|---|---|
| Journal: | *BMJ Open* |
| Manuscript ID | bmjopen-2022-067293.R2 |
| Article Type: | Original research |
| Date Submitted by the Author: | 17-May-2023 |
| Complete List of Authors: | Han, Conglin; Weifang Medical University; Beijing Rehabilitation Hospital,<br><br>Sun, Weishuang; Weifang Medical University<br>Zhang, Dan; Weifang Medical University<br>Xi, Xiaoshuang; Capital Medical University<br>Zhang, Rong; Yunnan University of Chinese Medicine<br>Gong, Weijun; Capital Medical University |
| <b>Primary Subject Heading</b>: | Rehabilitation medicine |
| Secondary Subject Heading: | Sports and exercise medicine, Geriatric medicine |
| Keywords: | NEUROLOGY, SPORTS MEDICINE, Aging, Aged |
| | |

SCHOLARONE™
Manuscripts

*I, the Submitting Author has the right to grant and does grant on behalf of all authors of the Work (as defined in the below author licence), an exclusive licence and/or a non-exclusive licence for contributions from authors who are: i) UK Crown employees; ii) where BMJ has agreed a CC-BY licence shall apply, and/or iii) in accordance with the terms applicable for US Federal Government officers or employees acting as part of their official duties; on a worldwide, perpetual, irrevocable, royalty-free basis to BMJ Publishing Group Ltd ("BMJ") its licensees and where the relevant Journal is co-owned by BMJ to the co-owners of the Journal, to publish the Work in this journal and any other BMJ products and to exploit all rights, as set out in our licence.*

*The Submitting Author accepts and understands that any supply made under these terms is made by BMJ to the Submitting Author unless you are acting as an employee on behalf of your employer or a postgraduate student of an affiliated institution which is paying any applicable article publishing charge ("APC") for Open Access articles. Where the Submitting Author wishes to make the Work available on an Open Access basis (and intends to pay the relevant APC), the terms of reuse of such Open Access shall be governed by a Creative Commons licence – details of these licences and which Creative Commons licence will apply to this Work are set out in our licence referred to above.*

*Other than as permitted in any relevant BMJ Author's Self Archiving Policies, I confirm this Work has not been accepted for publication elsewhere, is not being considered for publication elsewhere and does not duplicate material already published. I confirm all authors consent to publication of this Work and authorise the granting of this licence.*

For peer review only

# Effects of different aerobic exercises on the global cognitive function of the elderly with mild cognitive impairment: a meta-analysis

Conglin Han[1], Weishuang Sun[1], Dan Zhang[1] , Xiaoshuang Xi[2], Rong Zhang[3], Weijun Gong[4*]

1 Beijing Rehabilitation Hospital, Capital Medical University, Beijing 100144, China
Rehabilitation Medicine Academy, Weifang Medical University, Shandong 261053, China
2 Beijing Rehabilitation Medicine Academy, Capital Medical University, Beijing 100144, China
3 Second Clinical Medical Academy, Yunnan University of Chinese Medicine, Yunnan 650500, China
4 Department of Neurological Rehabilitation, Beijing Rehabilitation Hospital, Capital Medical University, Beijing 100144, China
*Corresponding author: Weijun Gong, Correspondence: E-mail: gwj197104@ccmu.edu.cn.

ORCID：0000-0002-9134-8218 (Weijun Gong)

**Abstract**

Objectives: To summarize the effects of various types of aerobic exercise on the global cognitive function of the elderly with mild cognitive impairment(MCI).

Design: A meta-analysis of randomized controlled trials.

Data sources: PubMed, EMBASE, and the Cochrane Library were searched for clinical randomized controlled trials from the earliest available records to March 2022.

Eligibility criteria for selecting studies: We included randomized controlled trials of subjects older than 60 years with mild cognitive impairment. The outcome indicators of cognitive function of interest were the Mini-Mental State Examination (MMSE) and the Montreal Cognitive Assessment (MoCA).

Data extraction and synthesis: Two researchers independently screened the literature, extracted data and evaluated the quality of the included studies, with disagreements resolved by a third researcher. The Cochrane Handbook for Systematic Reviews of Interventions was used to assess the risk of bias. Meta-analysis was performed by RevMan V.5.3 software. Random effect models were used for meta-analysis.

Results: A total of 1680 patients who participated in 20 randomized controlled trials were included in this study. Based on outcomes of MMSE analysis, the aerobic exercise, which was beneficial for global cognitive function in MCI patients, included multi-component aerobic exercise (MD=1.79, 95% CI [1.41, 2.17], $p<0.01$) and mind-body exercise (MD=1.29, 95% CI [0.67, 1.90], $p<0.01$). The results of the meta-analysis of conventional aerobic exercise (MD=0.51, 95% CI [0.09, 0.93 ], $p<0.05$) turned out to be statistically insignificant after sensitivity analysis(MD=0.51,95% CI [0.09,0.93], $p=0.65$). With the evaluation of MoCA, multi-component aerobic exercise (MD=5.54,95% CI [5.02, 6.46], $p<0.01$), mind-body exercise (MD=1.29,95% CI [0.67, 1.90], $p<0.01$) and conventional aerobic exercise (MD=2.06,95% CI [1.46, 2.65], $p< 0.01$) were showed significant beneficial effects for the patient. However, there was a high degree of heterogeneity between the results of multi-component aerobic exercise (MMSE) and conventional aerobic exercise (MoCA), which was analyzed and explored.

Conclusions: In general, multi-component aerobic exercise and mind-body exercise were beneficial in improving global cognitive function in the elderly with mild cognitive impairment. Nevertheless, the improvement effect of mind-body exercise is more reliable compared to multi-component aerobic exercise and conventional aerobic exercise.

Keywords: Mild cognitive impairment, aerobic exercise, elderly



For peer review only - http://bmjopen.bmj.com/site/about/guidelines.xhtml

**Strengths and limitations of this study**

- A rigorous methodology was used for this study, including clear eligibility criteria, an extensive database search, study selection by two reviewers working independently, and risk of bias assessment.
- This protocol is based on the Preferred Reporting Items for Systematic Reviews and Meta-Analyses (PRISMA) project and the Cochrane Handbook for Systematic Reviews of Interventions.
- This study is the first to classify aerobic exercise and conduct a meta-analysis to explore the effects of different aerobic exercises on cognitive function in older adults with mild cognitive impairment.
- This meta-analysis will be used to inform future research to guide and improve aerobic exercise training practices in older adults with mild cognitive impairment.
- This study only analyzed changes in global cognitive functioning and did not include changes in specific cognitive domains.

## 1. Introduction

Along with the development and improvement in modern science, medical technology, and treatment, the average life expectancy of people around the world has been continuously extended. Age-related health problems are becoming more and more prominent. Dementia is one of the common diseases of aging and a major cause of disability in the elderly[1]. The World Health Organization estimates that about 50 million people worldwide suffer from dementia, with a new case every three seconds, and the number of people with dementia will triple by 2050[2]. Severe dementia can place a huge emotional and financial burden on families and society, so it is important to take measures for early detection, early intervention, and early treatment of dementia. Mild cognitive impairment (MCI) is an intermediate state between normal cognition and dementia. MCI usually refers to cognitive decline beyond that caused by normal aging, but without significant impairment in Activity of Daily Living (ADL)[3]. Currently, medications have a limited role in preventing and treating MCI[4,5], so there is a large body of research focusing on non-pharmacological interventions.

It has been suggested that some non-pharmacological interventions, such as food, exercise, and cognitive stimulation, may be beneficial for the improvement of cognitive function in MCI patients[3,4,6]. It has also shown that physical activity has a greater influence on the cognitive performance of MCI patients than drugs. For the elderly population with a decline in cognitive function, several studies have also confirmed that aerobic exercise appears to be effective in improving cognitive function[7-9]. However, there are various types of aerobic exercise, and the aerobic exercise that can be performed may vary in different populations (For example, it is difficult for people with lower limb mobility restrictions to run). As a result, an analysis is needed to compare the effects of different types of aerobic exercise on the improvement of global cognitive function in the elderly with MCI.

## 2. Methods

This study has been registered in the International Prospective Systematic Evaluation Registry database (PROSPERO, http://www.cdr.york.ac.uk/PROSPERO/) under the registration number: CRD42022327386. This study was conducted following the Preferred Reporting Items for Systematic Reviews and Meta-Analyses (PRISMA) statement.

### 2.1. Inclusion criteria

Types of studies: Human subject studies designed as randomized controlled trials (RCT) published in English were included without year restriction.

Types of participants: Subjects defined as MCI by the original authors and aged >60 years were included. We excluded healthy aging elderly adults, patients with any form of dementia, or those diagnosed with cognitive impairment due to definite etiologies, such as trauma or vascular or psychiatric diseases. Any clinical subtype of MCI was considered eligible.

Types of interventions: We included supervised structured exercises of any frequency, intensity, duration, or time directed at improving physical fitness. The interventions were various forms of exercise containing aerobic exercise, including conventional aerobic exercise -(simple and common aerobic exercises such as running, brisk walking, and cycling), mind-body exercise (Mind–body exercise focuses on mind, body, psychology, and behavior, including breathing and physical exercise, meditation, and so on[10]. It is a type of aerobic exercise of low to moderate intensity, with common forms such as tai chi, yoga, and dance[11,12].), and multi-component aerobic exercise (aerobic exercise combined with other forms of exercise, such as resistance training, balance training). Aerobic exercise was defined as the involvement of major muscle groups throughout the body in addition to the primary involvement of oxygen for energy supply.

Types of control groups: Controls could be exercises of stretching, activities of health education, routine care, daily lifestyle, and social recreation.

Types of outcomes: The results are presented using measurable cognitive screening instruments: the Mini-Mental State Examination (MMSE) and the Montreal Cognitive Assessment (MoCA). and are available as mean (M) and standard deviation (SD).

### 2.2. Exclusion criteria

Intervention methods could only be a variety of exercise training modalities; those with specific instructions to have other training components, such as cognitive training, were excluded. Non-intervention studies, articles with only research protocols, review articles, unpublished studies, abstracts or papers, and articles for which full text or data were not available were excluded. Non-English articles were excluded.

### 2.3. Search strategy

We systematically searched electronic databases in PubMed, EMBASE and the Cochrane Library from the earliest available records to March 2022. We searched using subject terms and free words. The keywords used included search terms related to sports exercise (e.g., exercise or physical activity or aerobic exercise) and cognitive impairment (e.g., mild cognitive impairment or cognitive dysfunction or amnestic mild cognitive impairment), and search terms related to older adults ( e.g., older adults or older patients). The search strategies for Embase, Pubmed and the Cochrane Library are described in Supplementary Document 1. Endnote X9.1 was used to store and sort the retrieved randomized controlled trials and to remove duplicates. Initial screening was performed by the first author

For peer review only



For peer review only - http://bmjopen.bmj.com/site/about/guidelines.xhtml

based on the title and abstract. This was followed by an independent screening of the full text by two reviewers according to the eligibility criteria specified in the study protocol. Disagreements in included studies were resolved by negotiation.

### 2.4. Data extraction and analysis

A standardized data extraction form was applied to extract data based on the following parameters: (1) basic information about the included studies (author, year, country) (2) basic characteristics of the study population (sample size, age, and gender of participants) (3) intervention characteristics (type of intervention, the intervention specifics, frequency and intensity of the intervention) (4) Outcome measures: we chose the two most commonly used scales for assessing global cognitive functioning, the MMSE and the MoCA. We performed a meta-analysis using the endpoint measures. Firstly, we conducted a meta-analysis of all aerobic exercise studies to obtain the results of the meta-analysis of MMSE and MoCA. After that, according to the type of aerobic exercise, they were divided into three groups (multi-component aerobic exercise, mind-body exercise and conventional aerobic exercise) and meta-analysis was conducted separately. For each type of aerobic exercise, meta-analysis results of MMSE and MoCA were also obtained.

For each outcome, the effect sizes are reported as the mean difference with 95% confidence intervals (CI) for each study. We pooled these values and conducted the meta-analysis using the Review Manager (RevMan) version 5.3. Heterogeneity between trial design options was unclear; therefore, a random-effects model was chosen. Our random effect model was based on the postulation that effect estimates were not the same but followed a normal distribution. While the area of the black square in forest plots denotes the weighted contribution of each study, $P < 0.05$ (two-tailed) was considered statistically significant. The $I^2$ statistic was computed as a measure of heterogeneity[13]. Cochrane's handbook recommends heterogeneity as not important (0–40%), moderate heterogeneity (30–60%), substantial heterogeneity (50–90%), and considerable heterogeneity (75–100%). Sensitivity analysis evaluated the stability of the results by leave-one-out method. Subgroup analyses were used to address significant clinical heterogeneity, or only descriptive analyses were performed.

### 2.5. Risk of Bias and quality assessment

The Cochrane risk of bias assessment tool was used to independently assess the risk of bias in included trials. The assessment covered the following parameters: random sequence generation, allocation concealment, blinding, incomplete data, selective reporting, and others ( Cochrane Handbook for Systematic Reviews of Interventions 8.5 ) . Each parameter was divided into three categories: low risk, unclear, and high risk. Each study was categorized using the following criteria: low risk of bias (all parameters were rated as low risk); moderate risk of bias (with parameters rated as unclear and no parameters rated as high risk); and high risk of bias (as long as one or more parameters were rated as high risk)( Cochrane Handbook for Systematic Reviews of Interventions Table 8.7.a). Two reviewers ( C H and W S ) assessed each trial independently. Any disagreements were discussed with a third reviewer ( W G ) until a final decision was achieved.

### 2.6. Assessment of evidence quality

The overall quality of the evidence was assessed using the Recommendations for Assessment, Development and Evaluation (GRADE) criteria[14]. In the system, the quality of evidence is classified into four levels: high quality, moderate quality, low quality, and very low quality[14].

### 2.7. Patient and public involvement

There was no direct patient or public involvement in this review.

## 3. Results

### 3.1. Search results

By searching electronic databases and literature, we obtained a total of 24,255 relevant references. After duplicates were removed, and titles and abstracts were checked against predefined inclusion criteria, 102 complete references were retained, and we reviewed each paper in detail. A total of 84 trials did not meet the predefined inclusion criteria: non-randomized controlled trials (n=4), no control group meeting the criteria (n=5), full text not available (n=6), assessment results not meeting the criteria (n=23), the study without results (n=9), data not available (n=13), intervention modality not meeting the pre-defined criteria (n=7), and study population not meeting the inclusion criteria (n=17). Therefore, a total of 18 articles were included in this study. The search process is shown in Figure 1.

### 3.2 Research Characteristics

A total of 18 randomized controlled trials were included in this study, published from 2011 to 2022. The characteristics of all included studies are summarized in Supplementary Document 2. The studies were conducted in different countries: China (n=8), the United States (n=2), Brazil (n=2), Japan (n=1), Korea (n=1), Turkey (n=1), Iran (n=1), Greece (n=1), and Spain (n=1). All included participants were 1700 and they were assigned to either the experimental or control group, and the sample size of the study ranged from 19-389. All participants were diagnosed with MCI. One study[15] was composed of the mind-body exercise intervention group and the conventional aerobic intervention group with their corresponding control group. Another study[16] was designed as two groups with different exercise intensities (40% and 60% reserve heart rate) for the conventional aerobic intervention. For all included studies, 5 experimental groups underwent multi-component exercise training with aerobic exercise[17-21]; 7 experimental groups underwent mind-body exercise training[15,22-27]; 8 experimental groups underwent conventional aerobic exercise training[15,16,28-32]. The total intervention duration for all studies ranged from 6 weeks to 12 months. The frequency was 21-80 min per session 1-5 times per week. Three experimental groups had an exercise intensity of roughly low intensity[15-17], i.e., maximum heart rate <60% or Borg scale (level 10) <5 or Borg scale (level 20) <12 or metabolic equivalent during exercise (MET) <3 or reserve heart rate <59%; 15 experimental groups had an exercise intensity of roughly moderate to high intensity[15,16,18-20,22-29,31,32], i.e., maximum heart rate ≥60% or Borg scale (10th grade) ≥5 or Borg scale (20th grade)) ≥ 12 or metabolic equivalent during exercise (MET) ≥ 3 or reserve heart rate ≥ 59%; the intensity of exercise in



For peer review only - http://bmjopen.bmj.com/site/about/guidelines.xhtml

two other experimental groups was unclear[21,30].Ten studies assessed global cognitive function with the MMSE[16,18-21,26-28,31,32];5 studies assessed global cognitive function with the MoCA[15,22,24,29,30]; and 3 additional studies used both the MMSE and the MoCA[17,23,25].

### 3.3. Literature quality evaluation

As shown in Figure 2, the quality of studies included in the trials varied. Two trials had a low overall risk of bias[24,29], five trials had a moderate overall risk of bias[15,18,22,27,28], and eleven trials had a high overall risk of bias[16,17,19-21,23,25,26,30-32]. Regarding the method of randomization, 13 trials[15,17,18,20-25,29-32] had simple randomization and 5 trials[16,19,26-28] had stratified randomization. In terms of intervention allocation concealment, 9 trials[17,19-21,23,25,27,28,30] used open randomization tables, 3 trials[22,24,29] used sealed envelopes, and the remaining 6 trials[15,16,18,26,31,32] were unclear.

We conducted a sub-group analysis based on the quality of the studies in order to test the strength of the evidence. The relevant results are shown in Supplementary Document 3. Generally, only the moderate risk group of conventional aerobic exercise (MoCA) had statistically insignificant results and there was only one study in this group.

### 3.4 Results of meta-analysis of all included studies

The results of MMSE included a total of 14 trials, as shown in Figure 3. Meta-analysis of the random effects model showed that aerobic exercise was effective in improving overall cognitive function compared with the control group (MD=1.23,95% CI [0.99, 1.47], $p<0.01$). Significant heterogeneity was found in the study ($I^2=88\%$, $p<0.01$).

The results of MoCA included a total of 8 trials, as shown in Figure 3. Randomized effects model meta-analysis showed that aerobic exercise was effective in improving overall cognitive function compared with control group (MD=2.62,95% CI [1.14, 4.10], $p<0.01$). Significant heterogeneity was found in the study ($I^2=94\%$, $p<0.01$).

### 3.5 Results of meta-analysis of different aerobic exercises

All trials were divided into multi-component aerobic exercise, mind-body exercise and regular aerobic exercise by exercise modality, and the results of meta-analysis are shown in Figures 4 and 5. As can be seen from the figures, after grouping by exercise modality, the between-group differences in the two outcome indicators of MMSE(MD=1.39, 95% CI [0.62, 2.17], $p<0.01$) and MoCA ($I^2=97.6\%$, $p<0.01$) were statistically significant, indicating that the grouping was meaningful.

#### 3.5.1 Results of meta-analysis of multi-component aerobic exercise

As shown in Figure 4, multi-component aerobic exercise had a significant beneficial effect in the MMSE outcome index (MD=1.79, 95% CI [1.41, 2.17], $P<0.01$) but the heterogeneity was very high ($I^2=95\%$, $P<0.01$), so further analysis is needed. We performed a subgroup analysis of the multi-component intervention group and the results are shown in Supplementary Document 4. We performed a subgroup analysis based on the sample size, exercise intensity, region, Deviation risk rating and duration, frequency and total time in the trial. From the results of these analyses, it is clear that no meaningful results were obtained after subgroup analysis. As shown in Figure 5, there was only one multi-component exercise trial with a MoCA outcome and the result was (MD=5.54,95% CI [5.02, 6.46], $p<0.01$).

#### 3.5.2 Results of meta-analysis of mind-body exercise

As shown in Figure 4, in the MMSE outcome, the mind-body exercise intervention had a significant improvement effect (MD=1.28, 95% CI [0.83, 1.74], $p<0.01$) and was not highly heterogeneous ($I^2=50\%$, $p=0.11$). As shown in Figure 5, there was also a clinically significant effect of mind-body exercise in the results of MoCA (MD=1.29, 95% CI [0.67, 1.90], $p<0.01$) and its heterogeneity was also low ($I^2=6\%$, $p=0.36$).

#### 3.5.3 Results of meta-analysis of conventional aerobic exercise

As shown in Figure 4, in the outcome of MMSE, the improvement in global cognitive function was significant in the conventional aerobic exercise intervention group (MD=0.51, 95% CI [0.09, 0.93], $p=0.02$) and without heterogeneity ($I^2=0\%$, $p=0.48$). Figure 5 demonstrates the MoCA results for conventional aerobic exercise. The results are also significant (MD=2.06, 95% CI [1.46, 2.65], $p<0.01$), but the heterogeneity ($I^2=84\%$, $p=0.002$) is high requiring further analysis and interpretation.

### 3.6 rating the body of evidence

Supplementary Document 5 presents the GRADE evidence profile for the RCT outcomes. The quality of evidence varies from moderate to very low. For multi-component exercise, there is very low evidence(MMSE and MoCA) that it has a significant clinical effect on global cognitive function. For mind-body exercise, moderate(MMSE) and low(MoCA) evidence suggests a clinically significant effect on global cognitive function. For conventional aerobic exercise, there is low and very low evidence of a clinically significant effect on global cognitive function.

### 3.7 Sensitivity analysis

Using a leave-one-out method, we tested the effect of excluding individual studies on the stability of the effect estimates. We found no significant effect on effect estimates after excluding each study, except for the results for conventional aerobic exercise (MMSE). This suggests that the vast majority of the final results were stable. Regarding the MMSE results for conventional aerobic exercise, after sensitivity analysis, we found that the results were no longer significant after excluding the Wei XH 2014 study[32] (MD=0.51, 95% CI [0.09,0.93], p=0. 65). Details of the sensitivity analysis are presented in Supplementary Document 6.

## 4. Discussion

To the best of our knowledge, our meta-analysis explored for the first time the effect of different aerobic exercise types on global cognitive function in elderly patients with MCI. Our study analyzed improvements in cognitive function through endpoint indicators of global cognitive function and found that both multi-component aerobic exercise and mind-body exercise produced positive effects. In



For peer review only - http://bmjopen.bmj.com/site/about/guidelines.xhtml

addition, the results of conventional aerobic exercise were inconsistent and may not have a clinically significant effect on global cognitive function. The effects of conventional aerobic exercise need to be further investigated in a large number of high-quality studies.

**4.1.Summary of the literature**

There were few previous meta-analyses[33,34] that explored the effects of multi-component exercise on cognitive function in MCI. Although some of their results suggested a beneficial effect of multi-component aerobic exercise on cognitive function in the elderly with MCI, the intervention criteria for the studies included in these meta-analyses were not only exercise but also cognitive training and mental stimulation, which were not the same as in our study. The Meta-analysis by Borges -Machado et al. [35] discussed multi-component exercise, which was defined as exercise that combined aerobic, strength, posture, and balance training. Although the study found improvements in ADL in the subjects, the study was conducted in patients with dementia and the results did not determine the effects on cognitive function.

Previous studies[36,37] confirmed the beneficial effects of mind-body exercise on global cognitive function in people with MCI. Unlike the present study, they had slight differences in the type of trial studies, age, and outcome evaluation indicators in the inclusion criteria. Our inclusion criteria were relatively more stringent. These previous studies combined different cognitive evaluation metrics expressed as standardized mean differences (SMD). We selected the two most common indicators of global cognitive function evaluation (MMSE and MoCA) to be analyzed separately and expressed the results as mean difference (MD).

We did not find any meta-analysis related to conventional aerobic exercise in patients with MCI. Although there are a few reviews[9,38,39] reporting aerobic exercise in MCI patients, they did not analyze the types of aerobic exercise separately. Therefore, we believed that the analysis of the present study was still of practical relevance.

**4.2. Analysis of the results of mind-body exercise**

All studies of mind-body exercise reported positive results. The results of both the MMSE and MoCA meta-analyses were significant effects and low heterogeneity, which would seem to indicate the effectiveness of mind-body exercise in improving cognitive function in older adults with MCI. One possible explanation for the beneficial results of the mind-body exercise was that mind-body exercise was a form of physical exercise supplemented with different degrees of cognitive function training. For example, Tai Chi, a Chinese system of slow meditative physical exercise designed for mind relaxation and balance, required attention, memory, and other aspects in addition to physical activity. Adding cognitive training to physical activity seems to have better effects on cognitive function improvement than training alone[40]. Another possible explanation was that depression was an important risk factor for cognitive decline in people with MCI and that mind-body exercise improved mood in older adults better than other physical exercises[36].

**4.3. Heterogeneity analysis of multi-component aerobic exercise**

From a total of five studies analyzed by MMSE for multi-component aerobic exercise, two studies[19,21] were no meaningful change in outcomes. We analyzed these studies and speculated that the possible reasons for such high heterogeneity. In the trial studied by de Oliveira et al.[19], the authors indicated that participants in both the trial and control groups were receiving medication, but no further medication-related descriptions. We are not sure if the medication affected the cognitive function of the subjects and thus the effect of the intervention. In another trial[21] that reported negative results, each exercise type of its multi-component aerobic exercise was performed separately, whereas common multi-component exercises combine different components in a single exercise session[35]. This was therefore the most different aspect of this study from other multi-component exercise training, and one possible explanation was that different components were more effective when combined in a single exercise session. One study showed[41] that short-term tai chi training did not significantly improve cognitive function in adults when comparing long-term and short-term tai chi training. In another trial[17], the duration of aerobic exercise was too short at only 5 minutes. Thus we speculated that there may be some difference in the results produced by different time of aerobic exercise.

**4.4. Analysis of the results of Conventional aerobic exercise**

The preliminary MMSE results for conventional aerobic exercise were significant, which would seem to demonstrate the effectiveness of conventional aerobic exercise. However, after sensitivity analysis was performed to exclude this Wei XH 2014 study[32], this result became meaningless. This suggests that the MMSE results for conventional aerobic exercise are unstable and we cannot conclude the effectiveness of conventional aerobic exercise on this basis. In fact, despite the large number of studies and recommendations[9,42,43] that conclude that aerobic exercise in life is beneficial for cognitive function, there are still many evidence-based studies[44,45] that have yielded non-positive results. A four-year trial[46] showed that aerobic exercise alone was not sufficient to change cognitive function, and that aerobic exercise up to a certain intensity and in combination with other forms of exercise might have better results. This may be the reason why the results are not positive.

The results of the meta-analysis on MoCA produced a high level of heterogeneity, which we tried to explain. First of all, in the three studies[15,29,30] with this outcome, there was a larger proportion of female subjects, which may have made the studies unrepresentative. A previous study[47] demonstrated a sex bias in older adults regarding the effects of aerobic exercise on cognitive function, with older women experiencing more significant cognitive benefits from aerobic exercise interventions than men. econd, one of the included studies[15] had a small sample size, which may have made the results of the trial less reliable.

In summary, given the above two results, we are cautious about the effect of conventional aerobic exercise. We believe that the improvement in cognitive function with conventional aerobic exercise may not always be significant and that future studies could improve the quality of research and provide more in-depth studies and analyses of specific interventions.

**4.5. Limitations**

There were several limitations of this review. The first is the number of included studies. Although the total number of included studies is not small, the interventions primarily explored in this study were a variety of different aerobic exercises, so the small number of included studies for each type of aerobic exercise makes the interpretation of the results obtained rather difficult. Secondly, the quality of the studies we included varied, and low-quality studies may make the results inaccurate. The third is that there was considerable heterogeneity between some of the included studies, representing both clinical and methodological heterogeneity, due to differences between participants (For example, older adults may have unknown comorbidities and the possibility of taking medications



For peer review only - http://bmjopen.bmj.com/site/about/guidelines.xhtml

as a result, which may affect the effectiveness of the intervention), interventions, and study designs. Fourth, it cannot be excluded that certain control groups would also have different levels of benefit on cognitive function such as social activities[48]. Fifth, only some of the trials that assessed global cognitive outcomes were included, and not those that assessed separately cognitive domains. Finally,, only studies of trials with MMSE and MoCA outcomes were selected. Although these two cognitive evaluation metrics are the most commonly used, they also undoubtedly excluded other studies that might be of value.

### 4.6. Implications for future research

Future studies should be designed with rigorous methods as well as controlled for various biases to improve the quality of the study.Different types of aerobic exercise have different experimental interventions. The duration, frequency and intensity of the exercise intervention may have different degrees of influence on the results of the trial. Follow-up research protocols could focus on this to explore the 7design of better interventions. The exercise components of multi-component aerobic training are complex and future studies could investigate better combinations of exercise components to achieve better improvements.

In addition, previous studies have indicated that the MoCA is superior to the MMSE for screening diagnoses in MCI patients, making it more recommended to replace the MMSE with the MoCA for future scales measuring global cognitive function[49]. in addition to the MMSE and the MoCA, the CDR (Clinical Dementia Rating) is a worthwhile scale to refer to and use. In addition to the global cognitive function, it would be interesting to assess specific domains of cognitive function (e.g., memory, attention), which may allow the inclusion of additional studies and better delineate the sources of heterogeneity involved.

## 5. Conclusion

The results of this meta-analysis suggest that two types of aerobic exercise (including multi-component aerobic exercise and mind-body exercise) have a positive effect on improving global cognitive function in older patients with MCI. However, the conclusion should be treated cautiously due to the heterogeneity and limited research. Additional well-designed RCTs are needed to provide more evidence for this conclusion.

**Contributors:** All authors made a substantial contribution to this work. Conglin Han, Weishuang Sun, Dan Zhang, Xiaoshuang Xi, Rong Zhang and Weijun Gong all contributed to the conception and design of the review. Conglin Han drafted the paper with all the authors critically reviewing it and suggesting amendments prior to submission. All authors had full access to all of the data (including statistical reports and tables) in the study and can take responsibility for the integrity of the data and the accuracy of the data analysis.

**Competing interests:** There are no competing interests for any author.

**Funding:** This work was supported by Capital's Funds for Health Improvement and Research(2022-1-2251), Natural Science Foundation of China (81972148) and Beijing Natural Science Foundation of China (7222101).

**Data sharing statement:** All data relevant to the study are included in the article or uploaded as supplementary information.

**Ethics statements**

Patient consent for publication

Not applicable.

**Ethics approval**

This study does not directly involve human participants. Therefore, it is not applicable.

Figure 1: Study flow diagram.

Figure 2: Risk of bias assessment of included trials.

Figure 3: (1)Effect sizes of forest plots for aerobic exercise versus controls-MMSE outcomes. MMSE, mini-mental state examination.

(2)Effect sizes of forest plots for aerobic exercise versus controls-MoCA outcome. MoCA, Montreal Cognitive Assessment.

Figure 4: Effect sizes of forest plots for the different aerobic exercise-MMSE outcomes. MMSE, mini-mental state examination.

Figure 5: Effect sizes of forest plots for the different aerobic exercise-MoCA outcomes. MoCA, Montreal Cognitive Assessment

For peer review only

References

[1] The Lancet Public H. Reinvigorating the public health response to dementia[J]. Lancet Public Health, 2021, 6(10): e696.

[2] WHO Guidelines Approved by the Guidelines Review Committee, Risk Reduction of Cognitive Decline and Dementia: WHO Guidelines, Geneva: World Health Organization © World Health Organization 2019., 2019.

[3] Sanford A M. Mild Cognitive Impairment[J]. Clin Geriatr Med, 2017, 33(3): 325-337.

[4] Jongsiriyanyong S, Limpawattana P. Mild Cognitive Impairment in Clinical Practice: A Review Article[J]. Am J Alzheimers Dis Other Demen, 2018, 33(8): 500-507.

[5] Schneider L S, Mangialasche F, Andreasen N, et al. Clinical trials and late-stage drug development for Alzheimer's disease: an appraisal from 1984 to 2014[J]. J Intern Med, 2014, 275(3): 251-83.

[6] Eshkoor S A, Hamid T A, Mun C Y, et al. Mild cognitive impairment and its management in older people[J]. Clin Interv Aging, 2015, 10: 687-93.

[7] Law C K, Lam F M, Chung R C, et al. Physical exercise attenuates cognitive decline and reduces behavioural problems in people with mild cognitive impairment and dementia: a systematic review[J]. J Physiother, 2020, 66(1): 9-18.

[8] Ahlskog J E, Geda Y E, Graff-Radford N R, et al. Physical exercise as a preventive or disease-modifying treatment of dementia and brain aging[J]. Mayo Clin Proc, 2011, 86(9): 876-84.

[9] Yong L, Liu L, Ding T, et al. Evidence of Effect of Aerobic Exercise on Cognitive Intervention in Older Adults With Mild Cognitive Impairment[J]. Front Psychiatry, 2021, 12:



For peer review only - http://bmjopen.bmj.com/site/about/guidelines.xhtml

For peer review only

713671.

[10] Li Z, Liu S, Wang L, et al. Mind-Body Exercise for Anxiety and Depression in COPD Patients: A Systematic Review and Meta-Analysis[J]. Int J Environ Res Public Health, 2019, 17(1).

[11] Zhang T, Liu W, Gao S. Effects of mind-body exercises on cognitive impairment in people with Parkinson's disease: A mini-review[J]. Front Neurol, 2022, 13: 931460.

[12] Wu C, Yi Q, Zheng X, et al. Effects of Mind-Body Exercises on Cognitive Function in Older Adults: A Meta-Analysis[J]. J Am Geriatr Soc, 2019, 67(4): 749-758.

[13] Higgins J P, Altman D G, Gøtzsche P C, et al. The Cochrane Collaboration's tool for assessing risk of bias in randomised trials[J]. Bmj, 2011, 343: d5928.

[14] Brozek J L, Akl E A, Alonso-Coello P, et al. Grading quality of evidence and strength of recommendations in clinical practice guidelines. Part 1 of 3. An overview of the GRADE approach and grading quality of evidence about interventions[J]. Allergy, 2009, 64(5): 669-77.

[15] Tao J, Liu J, Chen X, et al. Mind-body exercise improves cognitive function and modulates the function and structure of the hippocampus and anterior cingulate cortex in patients with mild cognitive impairment[J]. Neuroimage. Clinical, 2019, 23: 101834.

[16] Varela S, Ayán C, Cancela J M, et al. Effects of two different intensities of aerobic exercise on elderly people with mild cognitive impairment: a randomized pilot study[J]. Clin Rehabil, 2012, 26(5): 442-50.

[17] Li L, Liu M, Zeng H, et al. Multi-component exercise training improves the physical and cognitive function of the elderly with mild cognitive impairment: a six-month randomized controlled trial[J]. Annals of palliative medicine, 2021, 10(8): 8919-8929.



For peer review only

For peer review only - http://bmjopen.bmj.com/site/about/guidelines.xhtml

[18] Langoni C D S, Resende T L, Barcellos A B, et al. Effect of Exercise on Cognition, Conditioning, Muscle Endurance, and Balance in Older Adults With Mild Cognitive Impairment: a Randomized Controlled Trial[J]. Journal of geriatric physical therapy (2001), 2019, 42(2): E15-E22.

[19] De Oliveira Silva F, Ferreira J V, Plácido J, et al. Three months of multimodal training contributes to mobility and executive function in elderly individuals with mild cognitive impairment, but not in those with Alzheimer's disease: a randomized controlled trial[J]. Maturitas, 2019, 126: 28-33.

[20] Bademli K, Lok N, Canbaz M, et al. Effects of Physical Activity Program on cognitive function and sleep quality in elderly with mild cognitive impairment: a randomized controlled trial[J]. Perspectives in psychiatric care, 2019, 55(3): 401-408.

[21] Lam L C, Chan W C, Leung T, et al. Would older adults with mild cognitive impairment adhere to and benefit from a structured lifestyle activity intervention to enhance cognition?: a cluster randomized controlled trial[J]. PloS one, 2015, 10(3): e0118173.

[22] Li F, Harmer P, Fitzgerald K, et al. A cognitively enhanced online Tai Ji Quan training intervention for community-dwelling older adults with mild cognitive impairment: a feasibility trial[J]. BMC geriatrics, 2022, 22(1): 76.

[23] Qi M, Zhu Y I, Zhang L, et al. The effect of aerobic dance intervention on brain spontaneous activity in older adults with mild cognitive impairment: A resting-state functional MRI study[J]. Experimental and Therapeutic Medicine, 2019, 17(1): 715-722.

[24] Zhu Y, Wu H, Qi M, et al. Effects of a specially designed aerobic dance routine on mild cognitive impairment[J]. Clinical interventions in aging, 2018, 13: 1691-1700.



For peer review only - http://bmjopen.bmj.com/site/about/guidelines.xhtml

[25] Lazarou I, Parastatidis T, Tsolaki A, et al. International Ballroom Dancing Against Neurodegeneration: a Randomized Controlled Trial in Greek Community-Dwelling Elders With Mild Cognitive impairment[J]. American journal of Alzheimer's disease and other dementias, 2017, 32(8): 489-499.

[26] Doi T, Verghese J, Makizako H, et al. Effects of Cognitive Leisure Activity on Cognition in Mild Cognitive Impairment: Results of a Randomized Controlled Trial[J]. Journal of the American Medical Directors Association, 2017, 18(8): 686-691.

[27] Lam L C, Chan W M, Kwok T C, et al. Effectiveness of Tai Chi in maintenance of cognitive and functional abilities in mild cognitive impairment: a randomised controlled trial[J]. Hong Kong Med J, 2014, 20(3 Suppl 3): 20-3.

[28] Tomoto T, Liu J, Tseng B Y, et al. One-Year Aerobic Exercise Reduced Carotid Arterial Stiffness and Increased Cerebral Blood Flow in Amnestic Mild Cognitive Impairment[J]. Journal of Alzheimer's disease, 2021, 80(2): 841-853.

[29] Song D, Yu D S F. Effects of a moderate-intensity aerobic exercise programme on the cognitive function and quality of life of community-dwelling elderly people with mild cognitive impairment: a randomised controlled trial[J]. International journal of nursing studies, 2019, 93: 97-105.

[30] Choi W, Lee S. Ground Kayak Paddling Exercise Improves Postural Balance, Muscle Performance, and Cognitive Function in Older Adults with Mild Cognitive Impairment: a Randomized Controlled Trial[J]. Medical science monitor, 2018, 24: 3909-3915.

[31] Kohanpour M-A, Mohammad-Ali K, Maghsoud P, et al. The effects of aerobic exercise with lavender essence use on cognitive state and serum brain-derived neurotrophic factor



For peer review only - http://bmjopen.bmj.com/site/about/guidelines.xhtml

levels in elderly with mild cognitive impairment[J]. Journal of herbmed pharmacology, 2017, 6(2): 80-84.

[32] Wei X H, Ji L L. Effect of handball training on cognitive ability in elderly with mild cognitive impairment[J]. Neuroscience letters, 2014, 566: 98-101.

[33] Özbe D, Graessel E, Donath C, et al. Immediate Intervention Effects of Standardized Multicomponent Group Interventions on People with Cognitive Impairment: A Systematic Review[J]. J Alzheimers Dis, 2019, 67(2): 653-670.

[34] Bruderer-Hofstetter M, Rausch-Osthoff A K, Meichtry A, et al. Effective multicomponent interventions in comparison to active control and no interventions on physical capacity, cognitive function and instrumental activities of daily living in elderly people with and without mild impaired cognition - A systematic review and network meta-analysis[J]. Ageing Res Rev, 2018, 45: 1-14.

[35] Borges-Machado F, Silva N, Farinatti P, et al. Effectiveness of Multicomponent Exercise Interventions in Older Adults With Dementia: A Meta-Analysis[J]. Gerontologist, 2021, 61(8): e449-e462.

[36] Wang S, Yin H, Jia Y, et al. Effects of Mind-Body Exercise on Cognitive Function in Older Adults With Cognitive Impairment: A Systematic Review and Meta-analysis[J]. J Nerv Ment Dis, 2018, 206(12): 913-924.

[37] Zou L, Loprinzi P D, Yeung A S, et al. The Beneficial Effects of Mind-Body Exercises for People With Mild Cognitive Impairment: a Systematic Review With Meta-analysis[J]. Arch Phys Med Rehabil, 2019, 100(8): 1556-1573.

[38] Cammisuli D M, Innocenti A, Franzoni F, et al. Aerobic exercise effects upon cognition in

For peer review only



For peer review only - http://bmjopen.bmj.com/site/about/guidelines.xhtml

Mild Cognitive Impairment: A systematic review of randomized controlled trials[J]. Arch Ital Biol, 2017, 155(1-2): 54-62.

[39] Zheng G, Xia R, Zhou W, et al. Aerobic exercise ameliorates cognitive function in older adults with mild cognitive impairment: a systematic review and meta-analysis of randomised controlled trials[J]. Br J Sports Med, 2016, 50(23): 1443-1450.

[40] Gheysen F, Poppe L, Desmet A, et al. Physical activity to improve cognition in older adults: can physical activity programs enriched with cognitive challenges enhance the effects? A systematic review and meta-analysis[J]. Int J Behav Nutr Phys Act, 2018, 15(1): 63.

[41] Walsh J N, Manor B, Hausdorff J, et al. Impact of Short- and Long-term Tai Chi Mind-Body Exercise Training on Cognitive Function in Healthy Adults: Results From a Hybrid Observational Study and Randomized Trial[J]. Glob Adv Health Med, 2015, 4(4): 38-48.

[42] WHO Guidelines Approved by the Guidelines Review Committee, Global Recommendations on Physical Activity for Health, Geneva: World Health Organization Copyright © World Health Organization 2010., 2010.

[43] Northey J M, Cherbuin N, Pumpa K L, et al. Exercise interventions for cognitive function in adults older than 50: a systematic review with meta-analysis[J]. Br J Sports Med, 2018, 52(3): 154-160.

[44] Gates N, Fiatarone Singh M A, Sachdev P S, et al. The effect of exercise training on cognitive function in older adults with mild cognitive impairment: a meta-analysis of randomized controlled trials[J]. Am J Geriatr Psychiatry, 2013, 21(11): 1086-97.

[45] Snowden M, Steinman L, Mochan K, et al. Effect of exercise on cognitive performance in community-dwelling older adults: review of intervention trials and recommendations for public



For peer review only - http://bmjopen.bmj.com/site/about/guidelines.xhtml

health practice and research[J]. J Am Geriatr Soc, 2011, 59(4): 704-16.

[46] Komulainen P, Tuomilehto J, Savonen K, et al. Exercise, diet, and cognition in a 4-year randomized controlled trial: Dose-Responses to Exercise Training (DR's EXTRA)[J]. Am J Clin Nutr, 2021, 113(6): 1428-1439.

[47] Baker L D, Frank L L, Foster-Schubert K, et al. Effects of aerobic exercise on mild cognitive impairment: a controlled trial[J]. Arch Neurol, 2010, 67(1): 71-9.

[48] James B D, Wilson R S, Barnes L L, et al. Late-life social activity and cognitive decline in old age[J]. J Int Neuropsychol Soc, 2011, 17(6): 998-1005.

[49] Pinto T C C, Machado L, Bulgacov T M, et al. Is the Montreal Cognitive Assessment (MoCA) screening superior to the Mini-Mental State Examination (MMSE) in the detection of mild cognitive impairment (MCI) and Alzheimer's Disease (AD) in the elderly?[J]. Int Psychogeriatr, 2019, 31(4): 491-504.

For peer review only

**Provenance and peer review:** Not commissioned; externally peer reviewed.

I [Conglin Han], the Submitting Author has the right to grant and does grant on behalf of all authors of the Work (as defined in the below author licence), an exclusive licence and/or a non-exclusive licence for contributions from authors who are: i) UK Crown employees; ii) where BMJ has agreed a CC BY licence shall apply, and/or iii) in accordance with the terms applicable for US Federal Government officers or employees acting as part of their official duties; on a worldwide, perpetual, irrevocable, royalty-free basis to BMJ Publishing Group Ltd ("BMJ") its licensees and where the relevant Journal is co-owned by BMJ to the co-owners of the Journal, to publish the Work in [JOURNAL NAME] and any other BMJ products and to exploit all rights, as set out in our licence [link]



For peer review only - http://bmjopen.bmj.com/site/about/guidelines.xhtml

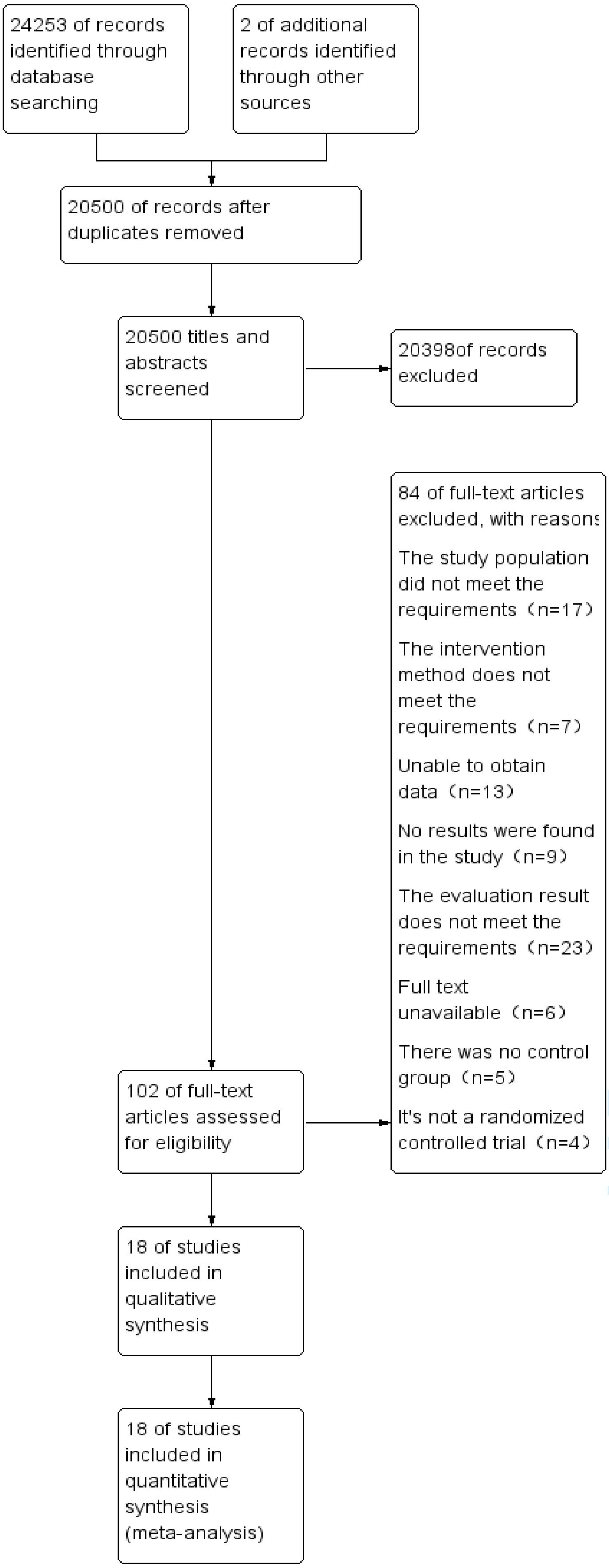


Figure 1: Study flow diagram.

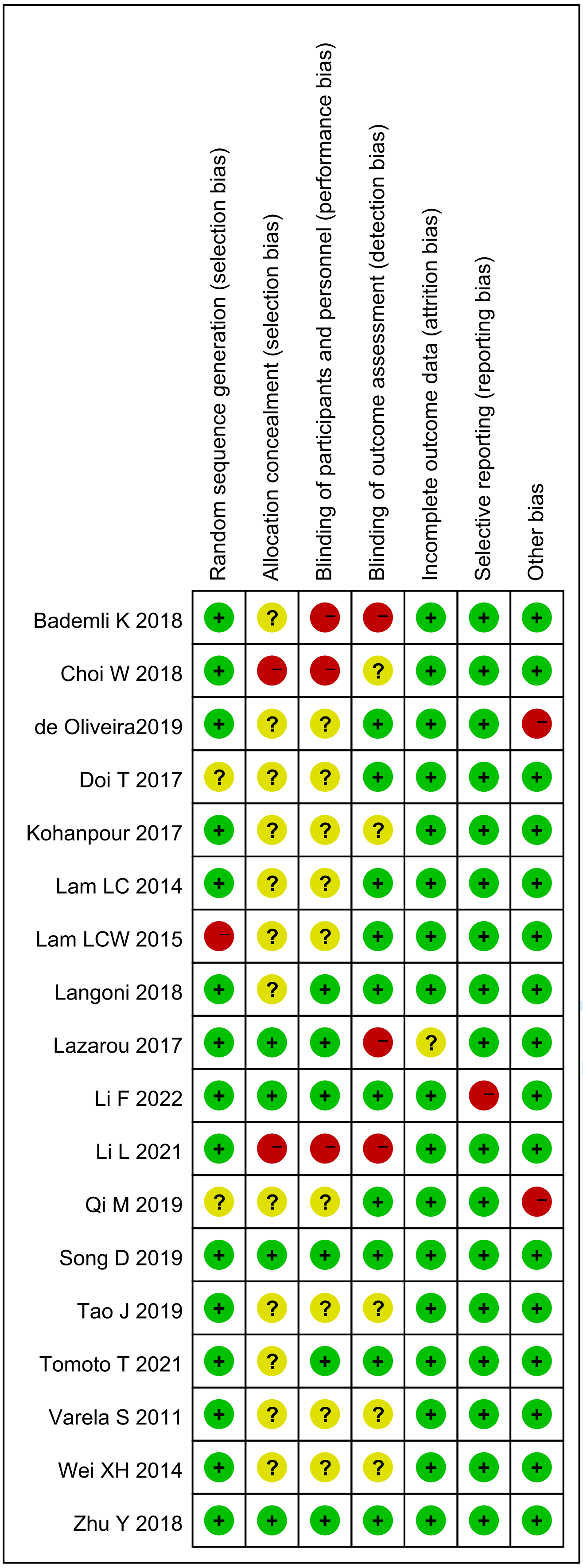


Figure 2: Risk of bias assessment of included trials.

| | Experimental | | | Control | | | | Mean Difference |
|---|---|---|---|---|---|---|---|---|
| Study or Subgroup | Mean | SD | Total | Mean | SD | Total | Weight | IV, Fixed, 95% CI |
| Bademli K 2019 | 26.542 | 1.84 | 30 | 22.24 | 1.15 | 30 | 9.6% | 4.30 [3.53, 5.08] |
| de Oliveira2019 | 28.25 | 0.92 | 12 | 28.04 | 2.2 | 7 | 2.0% | 0.21 [-1.50, 1.92] |
| Doi T 2017 | 26.29 | 2.6 | 55 | 25.44 | 2.18 | 63 | 7.6% | 0.85 [-0.02, 1.72] |
| Kohanpour 2017 | 24.4 | 1.42 | 10 | 24.2 | 0.63 | 10 | 6.2% | 0.20 [-0.76, 1.16] |
| Lam LC 2014 | 25.4 | 3.3 | 96 | 24.2 | 3.4 | 169 | 8.3% | 1.20 [0.36, 2.04] |
| Lam LCW 2015 | 25.6 | 2.4 | 147 | 25.5 | 2.5 | 131 | 17.3% | 0.10 [-0.48, 0.68] |
| Langoni 2019 | 25 | 4.7 | 26 | 20.4 | 4.1 | 26 | 1.0% | 4.60 [2.20, 7.00] |
| Lazarou 2017 | 28 | 2.39 | 66 | 25.65 | 3.27 | 63 | 5.9% | 2.35 [1.36, 3.34] |
| Li L 2021 | 27.79 | 1.18 | 42 | 25.42 | 2.28 | 42 | 9.6% | 2.37 [1.59, 3.15] |
| Qi M 2019 | 28.2 | 1 | 16 | 27.3 | 1.7 | 16 | 6.2% | 0.90 [-0.07, 1.87] |
| Tomoto T 2021 | 28.5 | 1.46 | 22 | 28.5 | 1.5 | 30 | 8.8% | 0.00 [-0.81, 0.81] |
| Varela S 2012① | 20.66 | 7.39 | 17 | 19.53 | 5.5 | 15 | 0.3% | 1.13 [-3.35, 5.61] |
| Varela S 2012② | 21.06 | 5.4 | 16 | 19.53 | 5.5 | 15 | 0.4% | 1.53 [-2.31, 5.37] |
| Wei XH 2014 | 25.53 | 0.82 | 30 | 24.67 | 1.42 | 30 | 16.8% | 0.86 [0.27, 1.45] |
| **Total (95% CI)** | | | **585** | | | **647** | **100.0%** | **1.23 [0.99, 1.47]** |

Heterogeneity: Chi² = 112.89, df = 13 (P < 0.00001); I² = 88%
Test for overall effect: Z = 10.04 (P < 0.00001)

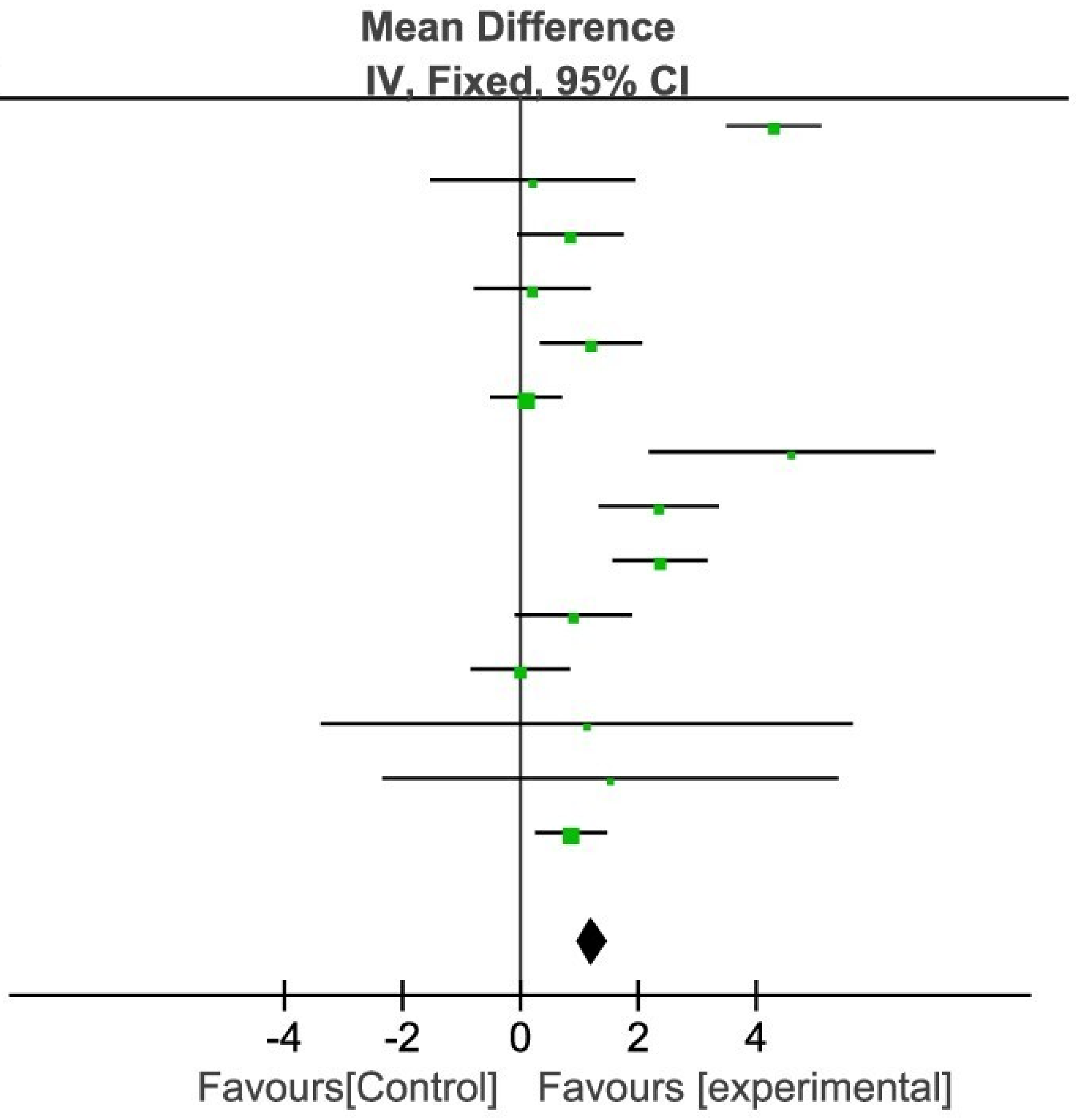


Figure 3: (1) Effect sizes of forest plots for aerobic exercise versus control-MMSE outcomes. MMSE, mini-mental state examination

| | Experimental | | | Control | | | | Mean Difference |
|---|---|---|---|---|---|---|---|---|
| Study or Subgroup | Mean | SD | Total | Mean | SD | Total | Weight | IV, Fixed, 95% CI |
| Choi W 2018 | 25.13 | 2.78 | 30 | 21.46 | 3.11 | 30 | 5.7% | 3.67 [2.18, 5.16] |
| Lazarou 2017 | 26 | 2.94 | 66 | 19.82 | 5.18 | 63 | 5.9% | 6.18 [4.72, 7.64] |
| Li F 2022 | 26.82 | 1.84 | 22 | 25.54 | 1.89 | 24 | 10.9% | 1.28 [0.20, 2.36] |
| Li L 2021 | 25.19 | 1.29 | 42 | 19.45 | 2 | 42 | 24.5% | 5.74 [5.02, 6.46] |
| Qi M 2019 | 24.3 | 2.2 | 16 | 23.7 | 2 | 16 | 6.0% | 0.60 [-0.86, 2.06] |
| Song D 2019 | 23.66 | 1.92 | 60 | 21.4 | 2.27 | 60 | 22.4% | 2.26 [1.51, 3.01] |
| Tao J 2019① | 24.55 | 2.33 | 20 | 22.1 | 2.59 | 20 | 5.4% | 2.45 [0.92, 3.98] |
| Tao J 2019② | 22.35 | 1.29 | 17 | 22.1 | 2.59 | 20 | 7.6% | 0.25 [-1.04, 1.54] |
| Zhu Y 2018 | 24.7 | 2.2 | 27 | 23.6 | 1.8 | 31 | 11.6% | 1.10 [0.06, 2.14] |
| **Total (95% CI)** | | | **300** | | | **306** | **100.0%** | **2.94 [2.58, 3.30]** |

Heterogeneity: Chi² = 129.08, df = 8 (P < 0.00001); I² = 94%
Test for overall effect: Z = 16.19 (P < 0.00001)

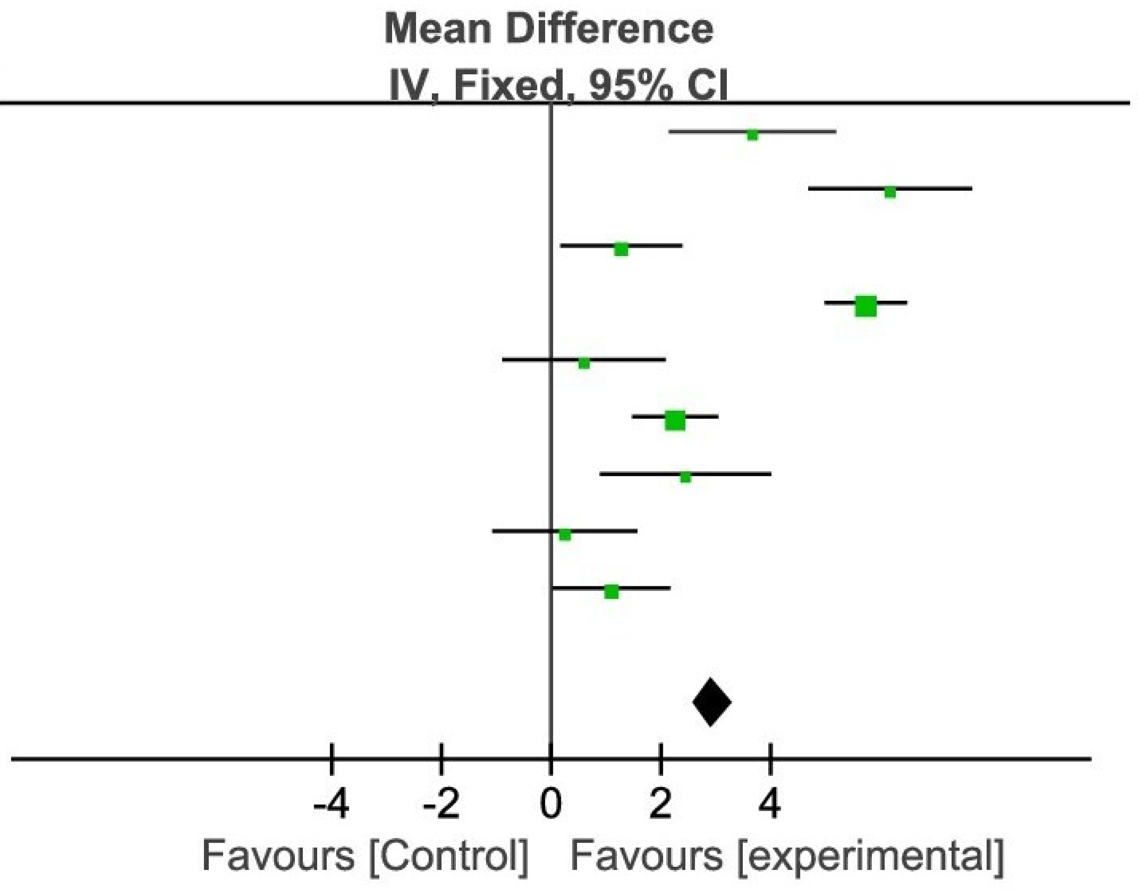


Figure 3: (2) Effect sizes of forest plots for aerobic exercise versus controls-MoCA outcome. MoCA, Montreal Cognitive Assessment.

| Study or Subgroup | Experimental Mean | Experimental SD | Experimental Total | Control Mean | Control SD | Control Total | Weight | Mean Difference IV, Fixed, 95% CI |
|---|---|---|---|---|---|---|---|---|
| **2.2.1 multi-component aerobic exercise VS controls** | | | | | | | | |
| Bademli K 2019 | 26.542 | 1.84 | 30 | 22.24 | 1.15 | 30 | 9.6% | 4.30 [3.53, 5.08] |
| de Oliveira2019 | 28.25 | 0.92 | 12 | 28.04 | 2.2 | 7 | 2.0% | 0.21 [-1.50, 1.92] |
| Lam LCW 2015 | 25.6 | 2.4 | 147 | 25.5 | 2.5 | 131 | 17.3% | 0.10 [-0.48, 0.68] |
| Langoni 2019 | 25 | 4.7 | 26 | 20.4 | 4.1 | 26 | 1.0% | 4.60 [2.20, 7.00] |
| Li L 2021 | 27.79 | 1.18 | 42 | 25.42 | 2.28 | 42 | 9.6% | 2.37 [1.59, 3.15] |
| **Subtotal (95% CI)** | | | **257** | | | **236** | **39.5%** | **1.79 [1.41, 2.17]** |
| Heterogeneity: Chi² = 83.77, df = 4 (P < 0.00001); I² = 95% | | | | | | | | |
| Test for overall effect: Z = 9.18 (P < 0.00001) | | | | | | | | |
| **2.2.2 mind-body exercise VS controls** | | | | | | | | |
| Doi T 2017 | 26.29 | 2.6 | 55 | 25.44 | 2.18 | 63 | 7.6% | 0.85 [-0.02, 1.72] |
| Lam LC 2014 | 25.4 | 3.3 | 96 | 24.2 | 3.4 | 169 | 8.3% | 1.20 [0.36, 2.04] |
| Lazarou 2017 | 28 | 2.39 | 66 | 25.65 | 3.27 | 63 | 5.9% | 2.35 [1.36, 3.34] |
| Qi M 2019 | 28.2 | 1 | 16 | 27.3 | 1.7 | 16 | 6.2% | 0.90 [-0.07, 1.87] |
| **Subtotal (95% CI)** | | | **233** | | | **311** | **28.0%** | **1.28 [0.83, 1.74]** |
| Heterogeneity: Chi² = 6.03, df = 3 (P = 0.11); I² = 50% | | | | | | | | |
| Test for overall effect: Z = 5.51 (P < 0.00001) | | | | | | | | |
| **2.2.3 Conventional aerobic exercise VS controls** | | | | | | | | |
| Kohanpour 2017 | 24.4 | 1.42 | 10 | 24.2 | 0.63 | 10 | 6.2% | 0.20 [-0.76, 1.16] |
| Tomoto T 2021 | 28.5 | 1.46 | 22 | 28.5 | 1.5 | 30 | 8.8% | 0.00 [-0.81, 0.81] |
| Varela S 2012① | 20.66 | 7.39 | 17 | 19.53 | 5.5 | 15 | 0.3% | 1.13 [-3.35, 5.61] |
| Varela S 2012② | 21.06 | 5.4 | 16 | 19.53 | 5.5 | 15 | 0.4% | 1.53 [-2.31, 5.37] |
| Wei XH 2014 | 25.53 | 0.82 | 30 | 24.67 | 1.42 | 30 | 16.8% | 0.86 [0.27, 1.45] |
| **Subtotal (95% CI)** | | | **95** | | | **100** | **32.5%** | **0.51 [0.09, 0.93]** |
| Heterogeneity: Chi² = 3.62, df = 4 (P = 0.46); I² = 0% | | | | | | | | |
| Test for overall effect: Z = 2.38 (P = 0.02) | | | | | | | | |
| **Total (95% CI)** | | | **585** | | | **647** | **100.0%** | **1.23 [0.99, 1.47]** |
| Heterogeneity: Chi² = 112.89, df = 13 (P < 0.00001); I² = 88% | | | | | | | | |
| Test for overall effect: Z = 10.04 (P < 0.00001) | | | | | | | | |
| Test for subgroup differences: Chi² = 19.47, df = 2 (P < 0.0001), I² = 89.7% | | | | | | | | |

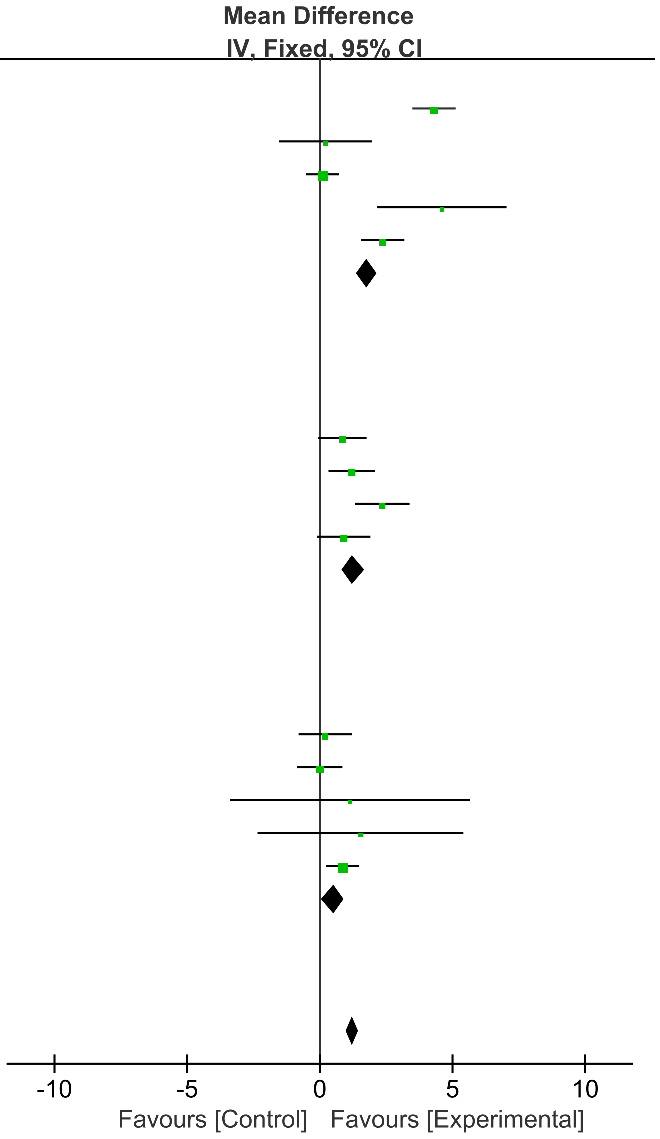


Figure 4: Effect sizes of forest plots for the different aerobic exercise-MMSE outcomes. MMSE, mini-mental state examination.

For peer review only

| Study or Subgroup | Experimental Mean | Experimental SD | Experimental Total | Control Mean | Control SD | Control Total | Weight | Mean Difference IV, Fixed, 95% CI |
|---|---|---|---|---|---|---|---|---|
| **3.2.1 multi-component aerobic exercise VS controls** | | | | | | | | |
| Li L 2021 | 25.19 | 1.29 | 42 | 19.45 | 2 | 42 | 26.0% | 5.74 [5.02, 6.46] |
| **Subtotal (95% CI)** | | | **42** | | | **42** | **26.0%** | **5.74 [5.02, 6.46]** |
| Heterogeneity: Not applicable | | | | | | | | |
| Test for overall effect: Z = 15.63 (P < 0.00001) | | | | | | | | |
| **3.2.2 mind-body exercise VS controls** | | | | | | | | |
| Li F 2022 | 26.82 | 1.84 | 22 | 25.54 | 1.89 | 24 | 11.6% | 1.28 [0.20, 2.36] |
| Qi M 2019 | 24.3 | 2.2 | 16 | 23.7 | 2 | 16 | 6.3% | 0.60 [-0.86, 2.06] |
| Tao J 2019① | 24.55 | 2.33 | 20 | 22.1 | 2.59 | 20 | 5.8% | 2.45 [0.92, 3.98] |
| Zhu Y 2018 | 24.7 | 2.2 | 27 | 23.6 | 1.8 | 31 | 12.4% | 1.10 [0.06, 2.14] |
| **Subtotal (95% CI)** | | | **85** | | | **91** | **36.1%** | **1.29 [0.67, 1.90]** |
| Heterogeneity: Chi² = 3.21, df = 3 (P = 0.36); I² = 6% | | | | | | | | |
| Test for overall effect: Z = 4.12 (P < 0.0001) | | | | | | | | |
| **3.2.3 Conventional aerobic exercise VS controls** | | | | | | | | |
| Choi W 2018 | 25.13 | 2.78 | 30 | 21.46 | 3.11 | 30 | 6.0% | 3.67 [2.18, 5.16] |
| Song D 2019 | 23.66 | 1.92 | 60 | 21.4 | 2.27 | 60 | 23.8% | 2.26 [1.51, 3.01] |
| Tao J 2019② | 22.35 | 1.29 | 17 | 22.1 | 2.59 | 20 | 8.1% | 0.25 [-1.04, 1.54] |
| **Subtotal (95% CI)** | | | **107** | | | **110** | **37.9%** | **2.06 [1.46, 2.65]** |
| Heterogeneity: Chi² = 12.30, df = 2 (P = 0.002); I² = 84% | | | | | | | | |
| Test for overall effect: Z = 6.76 (P < 0.00001) | | | | | | | | |
| **Total (95% CI)** | | | **234** | | | **243** | **100.0%** | **2.74 [2.37, 3.10]** |
| Heterogeneity: Chi² = 109.05, df = 7 (P < 0.00001); I² = 94% | | | | | | | | |
| Test for overall effect: Z = 14.61 (P < 0.00001) | | | | | | | | |
| Test for subgroup differences: Chi² = 93.54, df = 2 (P < 0.00001), I² = 97.9% | | | | | | | | |

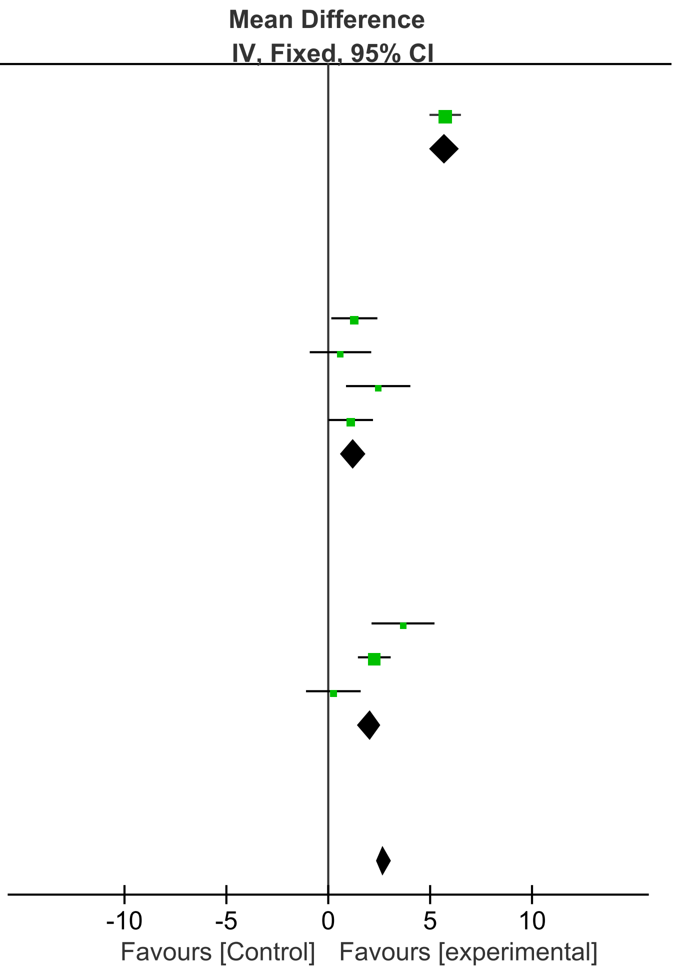


Figure 5: Effect sizes of forest plots for the different aerobic exercise-MoCA outcomes. MoCA, Montreal Cognitive Assessment

For peer review only

**Embase Search strategy**

| Search number | Search Details | Results |
|---|---|---|
| 1# | 'aerobic exercise'/exp | 19335 |
| 2# | 'aerobic dance':ab,ti OR 'aerobic dancing':ab,ti OR aerobics:ab,ti OR 'aerobics exercise':ab,ti OR 'dancing, aerobic':ab,ti OR 'exercise, aerobic':ab,ti OR 'low impact aerobic exercise':ab,ti OR 'low impact aerobics':ab,ti OR 'step aerobics':ab,ti | 1495 |
| 3# | 'aerobic exercise'/exp OR ('aerobic dance':ab,ti OR 'aerobic dancing':ab,ti OR aerobics:ab,ti OR 'aerobics exercise':ab,ti OR 'dancing, aerobic':ab,ti OR 'exercise, aerobic':ab,ti OR 'low impact aerobic exercise':ab,ti OR 'low impact aerobics':ab,ti OR 'step aerobics':ab,ti) | 20255 |
| 4# | 'exercise'/exp | 399161 |
| 5# | 'biometric exercise':ab,ti OR effort:ab,ti OR 'exercise capacity':ab,ti OR 'exercise performance':ab,ti OR 'exercise training':ab,ti OR exertion:ab,ti OR 'fitness training':ab,ti OR 'fitness workout':ab,ti OR 'physical conditioning':ab,ti OR human:ab,ti OR 'physical effort':ab,ti OR 'physical exercise':ab,ti OR 'physical exertion':ab,ti OR 'physical work-out':ab,ti OR 'physical workout':ab,ti | 3576566 |
| 6# | 'exercise'/exp OR ('biometric exercise':ab,ti OR effort:ab,ti OR 'exercise capacity':ab,ti OR 'exercise performance':ab,ti OR 'exercise training':ab,ti OR exertion:ab,ti OR 'fitness training':ab,ti OR 'fitness workout':ab,ti OR 'physical conditioning':ab,ti OR human:ab,ti OR 'physical effort':ab,ti OR 'physical exercise':ab,ti OR 'physical exertion':ab,ti OR 'physical work-out':ab,ti OR 'physical workout':ab,ti) | 3884161 |
| 7# | 'physical activity'/exp | 485896 |
| 8# | 'activity, physical':ab,ti | 1355 |
| 9# | 'physical activity'/exp OR 'activity, physical':ab,ti | 486507 |
| 10# | 'cognitive defect'/exp | 557350 |
| 11# | 'cognition disorders':ab,ti OR 'cognitive defects':ab,ti OR 'cognitive deficit':ab,ti OR 'cognitive disability':ab,ti OR 'cognitive disorder':ab,ti OR 'cognitive disorders':ab,ti OR 'cognitive dysfunction':ab,ti OR 'cognitive impairment':ab,ti OR 'delirium, dementia, amnestic, cognitive disorders':ab,ti OR overinclusion:ab,ti OR 'response interference':ab,ti | 136389 |
| 12# | 'cognitive defect'/exp OR ('cognition disorders':ab,ti OR 'cognitive defects':ab,ti OR 'cognitive deficit':ab,ti OR 'cognitive disability':ab,ti OR 'cognitive | 577692 |

| | | |
|---|---|---|
| | disorder':ab,ti OR 'cognitive disorders':ab,ti OR 'cognitive dysfunction':ab,ti OR 'cognitive impairment':ab,ti OR 'delirium, dementia, amnestic, cognitive disorders':ab,ti OR overinclusion:ab,ti OR 'response interference':ab,ti) | |
| 13# | 'mild cognitive impairment'/exp | 32022 |
| 14# | 'amnestic mild cognitive impairment':ab,ti | 3430 |
| 15 | 'mild cognitive impairment'/exp OR 'amnestic mild cognitive impairment':ab,ti | 32561 |
| 16# | ('aerobic exercise'/exp OR ('aerobic dance':ab,ti OR 'aerobic dancing':ab,ti OR aerobics:ab,ti OR 'aerobics exercise':ab,ti OR 'dancing, aerobic':ab,ti OR 'exercise, aerobic':ab,ti OR 'low impact aerobic exercise':ab,ti OR 'low impact aerobics':ab,ti OR 'step aerobics':ab,ti)) OR ('exercise'/exp OR ('biometric exercise':ab,ti OR effort:ab,ti OR 'exercise capacity':ab,ti OR 'exercise performance':ab,ti OR 'exercise training':ab,ti OR exertion:ab,ti OR 'fitness training':ab,ti OR 'fitness workout':ab,ti OR 'physical conditioning':ab,ti OR human:ab,ti OR 'physical effort':ab,ti OR 'physical exercise':ab,ti OR 'physical exertion':ab,ti OR 'physical work-out':ab,ti OR 'physical workout':ab,ti)) OR ('physical activity'/exp OR 'activity, physical':ab,ti) | 4258734 |
| 17# | ('cognitive defect'/exp OR ('cognition disorders':ab,ti OR 'cognitive defects':ab,ti OR 'cognitive deficit':ab,ti OR 'cognitive disability':ab,ti OR 'cognitive disorder':ab,ti OR 'cognitive disorders':ab,ti OR 'cognitive dysfunction':ab,ti OR 'cognitive impairment':ab,ti OR 'delirium, dementia, amnestic, cognitive disorders':ab,ti OR overinclusion:ab,ti OR 'response interference':ab,ti)) OR ('mild cognitive impairment'/exp OR 'amnestic mild cognitive impairment':ab,ti) | 577692 |
| 18# | (('aerobic exercise'/exp OR ('aerobic dance':ab,ti OR 'aerobic dancing':ab,ti OR aerobics:ab,ti OR 'aerobics exercise':ab,ti OR 'dancing, aerobic':ab,ti OR 'exercise, aerobic':ab,ti OR 'low impact aerobic exercise':ab,ti OR 'low impact aerobics':ab,ti OR 'step aerobics':ab,ti)) OR ('exercise'/exp OR ('biometric exercise':ab,ti OR effort:ab,ti OR 'exercise capacity':ab,ti OR 'exercise performance':ab,ti OR 'exercise training':ab,ti OR exertion:ab,ti OR 'fitness training':ab,ti OR 'fitness workout':ab,ti OR 'physical conditioning':ab,ti OR human:ab,ti OR 'physical effort':ab,ti OR 'physical exercise':ab,ti OR 'physical exertion':ab,ti OR 'physical work-out':ab,ti OR 'physical workout':ab,ti)) OR ('physical activity'/exp OR 'activity, physical':ab,ti)) AND (('cognitive defect'/exp OR ('cognition disorders':ab,ti OR 'cognitive defects':ab,ti OR 'cognitive deficit':ab,ti OR 'cognitive disability':ab,ti OR 'cognitive disorder':ab,ti OR 'cognitive disorders':ab,ti OR 'cognitive | 76351 |

For peer review only

| | | |
|---|---|---|
| | dysfunction':ab,ti OR 'cognitive impairment':ab,ti OR 'delirium, dementia, amnestic, cognitive disorders':ab,ti OR overinclusion:ab,ti OR 'response interference':ab,ti)) OR ('mild cognitive impairment'/exp OR 'amnestic mild cognitive impairment':ab,ti)) | |
| 19# | 'aged'/exp | 3469661 |
| 20# | 'aged patient':ab,ti OR 'aged people':ab,ti OR 'aged person':ab,ti OR 'aged subject':ab,ti OR elderly:ab,ti OR 'elderly patient':ab,ti OR 'elderly people':ab,ti OR 'elderly person':ab,ti OR 'elderly subject':ab,ti OR 'senior citizen':ab,ti OR senium:ab,ti | 385314 |
| 21# | 'aged'/exp OR ('aged patient':ab,ti OR 'aged people':ab,ti OR 'aged person':ab,ti OR 'aged subject':ab,ti OR elderly:ab,ti OR 'elderly patient':ab,ti OR 'elderly people':ab,ti OR 'elderly person':ab,ti OR 'elderly subject':ab,ti OR 'senior citizen':ab,ti OR senium:ab,ti) | 3555832 |
| 22# | ((('aerobic exercise'/exp OR ('aerobic dance':ab,ti OR 'aerobic dancing':ab,ti OR aerobics:ab,ti OR 'aerobics exercise':ab,ti OR 'dancing, aerobic':ab,ti OR 'exercise, aerobic':ab,ti OR 'low impact aerobic exercise':ab,ti OR 'low impact aerobics':ab,ti OR 'step aerobics':ab,ti)) OR ('exercise'/exp OR ('biometric exercise':ab,ti OR effort:ab,ti OR 'exercise capacity':ab,ti OR 'exercise performance':ab,ti OR 'exercise training':ab,ti OR exertion:ab,ti OR 'fitness training':ab,ti OR 'fitness workout':ab,ti OR 'physical conditioning':ab,ti OR human:ab,ti OR 'physical effort':ab,ti OR 'physical exercise':ab,ti OR 'physical exertion':ab,ti OR 'physical work-out':ab,ti OR 'physical workout':ab,ti)) OR ('physical activity'/exp OR 'activity, physical':ab,ti)) AND (('cognitive defect'/exp OR ('cognition disorders':ab,ti OR 'cognitive defects':ab,ti OR 'cognitive deficit':ab,ti OR 'cognitive disability':ab,ti OR 'cognitive disorder':ab,ti OR 'cognitive disorders':ab,ti OR 'cognitive dysfunction':ab,ti OR 'cognitive impairment':ab,ti OR 'delirium, dementia, amnestic, cognitive disorders':ab,ti OR overinclusion:ab,ti OR 'response interference':ab,ti)) OR ('mild cognitive impairment'/exp OR 'amnestic mild cognitive impairment':ab,ti))) AND ('aged'/exp OR ('aged patient':ab,ti OR 'aged people':ab,ti OR 'aged person':ab,ti OR 'aged subject':ab,ti OR elderly:ab,ti OR 'elderly patient':ab,ti OR 'elderly people':ab,ti OR 'elderly person':ab,ti OR 'elderly subject':ab,ti OR 'senior citizen':ab,ti OR senium:ab,ti)) | 16100 |

For peer review only

**Pubmed Search strategy**

| Search number | Search Details | Results |
|---|---|---|
| 1# | "Cognitive Dysfunction"[Mesh] | 228,279 |
| 2# | ((((((((((((((Exercises[Title/Abstract]) OR (Physical Activity[Title/Abstract])) OR (Physical Activities[Title/Abstract])) OR (Activities, Physical[Title/Abstract])) OR (Activity, Physical[Title/Abstract])) OR (Exercise*, Physical[Title/Abstract])) OR (Physical Exercise*[Title/Abstract])) OR (Acute Exercise*[Title/Abstract])) OR (Exercise*, Acute[Title/Abstract])) OR (Exercise*, Isometric[Title/Abstract])) OR (Isometric Exercise*[Title/Abstract])) OR (Exercise*, Aerobic[Title/Abstract])) OR (Aerobic Exercise*[Title/Abstract])) OR (Exercise Training*[Title/Abstract])) OR (Training*, Exercise[Title/Abstract]) | 293,650 |
| 3# | ("Exercise"[Mesh]) OR (((((((((((((((Exercises[Title/Abstract]) OR (Physical Activity[Title/Abstract])) OR (Physical Activities[Title/Abstract])) OR (Activities, Physical[Title/Abstract])) OR (Activity, Physical[Title/Abstract])) OR (Exercise*, Physical[Title/Abstract])) OR (Physical Exercise*[Title/Abstract])) OR (Acute Exercise*[Title/Abstract])) OR (Exercise*, Acute[Title/Abstract])) OR (Exercise*, Isometric[Title/Abstract])) OR (Isometric Exercise*[Title/Abstract])) OR (Exercise*, Aerobic[Title/Abstract])) OR (Aerobic Exercise*[Title/Abstract])) OR (Exercise Training*[Title/Abstract])) OR (Training*, Exercise[Title/Abstract])) | 409,704 |
| 4# | "Cognitive Dysfunction"[Mesh] | 29,147 |
| 5# | (((((((((((((Cognitive Dysfunctions[Title/Abstract]) OR (Dysfunction*, Cognitive[Title/Abstract])) OR (Cognitive Impairment*[Title/Abstract])) OR (Impairment*, Cognitive[Title/Abstract])) OR (Mild Cognitive Impairment*[Title/Abstract])) OR (Cognitive Impairment*, Mild[Title/Abstract])) OR (Impairment*, Mild Cognitive[Title/Abstract])) OR (Mild Neurocognitive Disorder*[Title/Abstract])) OR (Disorder*, Mild Neurocognitive[Title/Abstract])) OR (Neurocognitive Disorder*, Mild[Title/Abstract])) OR (Cognitive Decline*[Title/Abstract])) OR (Decline*, Cognitive[Title/Abstract])) OR (Mental Deterioration*[Title/Abstract])) OR (Deterioration*, Mental[Title/Abstract]) | 169,309 |
| 6# | ("Cognitive Dysfunction"[Mesh]) OR ((((((((((((((Cognitive Dysfunctions[Title/Abstract]) OR (Dysfunction*, Cognitive[Title/Abstract])) OR (Cognitive Impairment*[Title/Abstract])) OR (Impairment*, Cognitive[Title/Abstract])) OR (Mild Cognitive Impairment*[Title/Abstract])) OR (Cognitive Impairment*, Mild[Title/Abstract])) OR (Impairment*, Mild Cognitive[Title/Abstract])) OR (Mild Neurocognitive Disorder*[Title/Abstract])) OR (Disorder*, Mild Neurocognitive[Title/Abstract])) OR (Neurocognitive Disorder*, Mild[Title/Abstract])) OR (Cognitive Decline*[Title/Abstract])) OR (Decline*, Cognitive[Title/Abstract])) OR (Mental Deterioration*[Title/Abstract])) OR (Deterioration*, Mental[Title/Abstract])) | 171,419 |
| 7# | (("Exercise"[Mesh]) OR (((((((((((((((Exercises[Title/Abstract]) OR (Physical Activity[Title/Abstract])) OR (Physical Activities[Title/Abstract])) OR (Activities, Physical[Title/Abstract])) OR (Activity, Physical[Title/Abstract])) OR (Exercise*, Physical[Title/Abstract])) OR (Physical | 6,686 |

Exercise*[Title/Abstract])) OR (Acute Exercise*[Title/Abstract])) OR (Exercise*, Acute[Title/Abstract])) OR (Exercise*, Isometric[Title/Abstract])) OR (Isometric Exercise*[Title/Abstract])) OR (Exercise*, Aerobic[Title/Abstract])) OR (Aerobic Exercise*[Title/Abstract])) OR (Exercise Training*[Title/Abstract])) OR (Training*, Exercise[Title/Abstract]))) AND (("Cognitive Dysfunction"[Mesh]) OR ((((((((((((((Cognitive Dysfunctions[Title/Abstract]) OR (Dysfunction*, Cognitive[Title/Abstract])) OR (Cognitive Impairment*[Title/Abstract])) OR (Impairment*, Cognitive[Title/Abstract])) OR (Mild Cognitive Impairment*[Title/Abstract])) OR (Cognitive Impairment*, Mild[Title/Abstract])) OR (Impairment*, Mild Cognitive[Title/Abstract])) OR (Mild Neurocognitive Disorder*[Title/Abstract])) OR (Disorder*, Mild Neurocognitive[Title/Abstract])) OR (Neurocognitive Disorder*, Mild[Title/Abstract])) OR (Cognitive Decline*[Title/Abstract])) OR (Decline*, Cognitive[Title/Abstract])) OR (Mental Deterioration*[Title/Abstract])) OR (Deterioration*, Mental[Title/Abstract])))

**Cochrane Library Search strategy**

| Search number | Search Details | Results |
|---|---|---|
| 1# | MeSH descriptor: [Exercise] explode all trees | 32772 |
| 2# | (Exercise*, Acute):ti,ab,kw OR (Acute Exercise*):ti,ab,kw OR (Training*, Exercise):ti,ab,kw OR (Exercise Training*):ti,ab,kw OR (Exercise*, Isometric):ti,ab,kw (Word variations have been searched) | 52015 |
| 3# | (Exercise*, Aerobic):ti,ab,kw OR (Aerobic Exercise*):ti,ab,kw OR (Physical Exercise*):ti,ab,kw OR (Physical Activity):ti,ab,kw OR (Activity, Physical):ti,ab,kw (Word variations have been searched) | 97132 |
| 4# | (Activities, Physical):ti,ab,kw OR (Exercise*, Physical):ti,ab,kw OR (Physical Activitie):ti,ab,kw (Word variations have been searched) | 68139 |
| 5# | #2 OR #3 OR #4 | 12098 |
| 6# | #1 OR #5 | 130117 |
| 7# | MeSH descriptor: [Cognitive Dysfunction] explode all trees | 2809 |
| 8# | (Impairment*, Mild Cognitive):ti,ab,kw OR (Disorder*, Mild Neurocognitive):ti,ab,kw OR (Mild Neurocognitive Disorder*):ti,ab,kw OR (Mild Cognitive Impairment*):ti,ab,kw OR (Neurocognitive Disorder*, Mild):ti,ab,kw (Word variations have been searched) | 5434 |
| 9# | (Cognitive Impairment*, Mild):ti,ab,kw OR (Dysfunction*, Cognitive):ti,ab,kw OR (Impairment*, Cognitive):ti,ab,kw OR (Cognitive Dysfunctions):ti,ab,kw OR (Cognitive Impairment*):ti,ab,kw (Word variations have been searched) | 23085 |
| 10# | (Mental Deterioration*):ti,ab,kw OR (Decline*, Cognitive):ti,ab,kw OR (Deterioration*, Mental;):ti,ab,kw OR (Cognitive Decline*):ti,ab,kw (Word variations have been searched) | 8024 |
| 11# | #7 OR #8 OR #9 OR #10 | 28106 |
| 12# | #6 AND #11 | 4714 |
| 13# | MeSH descriptor: [Aged] explode all trees | 242328 |
| 14# | (Elderly):ti,ab,kw (Word variations have been searched) | 57985 |
| 15# | #13 OR #14 | 283159 |
| 16# | #12 AND #15 | 1492 |

For peer review only

Supplementary Table 1： Baseline characteristics of included trials

| Aerobic exercise type | study | region | | Participants | | | intervene | | | | |
|---|---|---|---|---|---|---|---|---|---|---|---|
| | | | | Sample size | age | female | manner | frequency | strength | MMSE | MoCA |
| multi-component aerobic exercise | Li, L (2021)[12] | Asia/ China | Experimental group | 42 | 60–69（47.6%）、70–79（47.6%）、80 and above（4.8%） | 64% | 5 minutes warm-up, 5 minutes aerobic training, 5 minutes strength training, 5 minutes balance training, 5 minutes coordination training, 5 minutes sensitivity training | 30 minutes × 5 times× 24 weeks (6 months). | The warm-up is a 3-4/10 Perceptual Nu (RPE) level, and the rest of the workout is 4–5/10 RPE | 26.50±1.33 | 21.52±2.05 |
| | | | Control group | 42 | 60–69（42.9%）、70–79（50.0%）、80 and above（7.1%） | 57% | General community health guidance | | | 26.62±1.46 | 21.14±1.97 |
| | Bademli (2019)[15] | Asian/ Turkey | Experimental group | 30 | 72.24± 7.16 | 60% | 10 minutes of warm-up, 20 minutes of rhythmic exercise (including 22 simple exercises), 10 minutes of cooling exercise (low-intensity exercise), 40 minutes of free walking. | 80 minutes ×20 weeks, 4/3/7 days per week | 3-6 metabolic equivalents (ME). | 23.27 ± 2.17 | |
| | | | Control group | 30 | 70.67 ± 8.34 | 57% | Continue with your daily activities | | | 23.42 ± 1.07 | |
| | De Oliveira (2019)[14] | Euro-American/ Brazil | Experimental group | 7 | 71.85 ± 5.69 | 85.71% | 5 minutes of balance training,30 minutes of aerobic training, 20 minutes of strength training, 5 minutes of stretching exercises | 60 minutes × 2 times × 12 weeks | The intensity of the main training is 70% of the maximum oxygen consumption (VO2max) or 80% of the predicted maximum heart rate (HRmax). | 29 ±0.61 | |
| | | | Control group | 12 | 78.20± 5.26 | 41.66% | Clinical follow-up without any physical training | | | 27.8 ±2.57 | |
| | Langoni (2019)[13] | Euro-American/ Brazil | Experimental group | 26 | 72.6 ±7.8 | 76.9% | Strength training takes about30 minutes (with equipment including ankle weight, resistance latex tape and dumbbells) and aerobic training (20-30 minutes of walking). | 60 minutes× 2 times × 24 weeks | Aerobic training to walk at a maximum heart rate of 60% to 75%. | 21.9 (4.8) | |
| | | | Control group | 26 | 71.9 ±7.9 | 76.9% | Do not initiate any form of physical or cognitive activity and keep in touch by phone | | | 25.0 (4.7) | |
| | Lam (2015)[16] | Asia/ China | Experimental group | 147 | 75.5±6.7 | 76.9% | Stretching and conditioning exercises, mind-body exercises (such as tai chi) and aerobic exercises (such as static cycling). | 120 minutes× 3×48 weeks (12 months). | Unknown | 25.8(2.3) | |

For peer review only

| | | | | | | | | | | | |
|---|---|---|---|---|---|---|---|---|---|---|---|
| | | | Control group | 131 | 75.4±6.1 | 77.9% | Social activities (e.g., tea parties, movie watching). | | | 25.6(2.4) | |
| mind-body exercise | Li,F (2022) | Euro-America/ United States | Experimental group | 22 | 74.5 ±5.6 | 36.4% | Traditional Tai Chi training | 60 minutes× 2 times × 16 weeks | The metabolic equivalent of Tai Chi exercise is 3.5-4.3 MET | | 25.09±2.43 |
| | | | Control group | 24 | 74.9±6.3 | 62.5% | Stretching exercises | | | | 25.13±2.19 |
| | Tao,J (2019)[10] ① | Asia/ China | Experimental group | 20 | 66.17±4.17 | 75% | 15 minutes to warm up, 40 minutes to eight danjin training and 5 minutes to cool down | 60 minutes × 3 times × 24 weeks | The Batdan Nishiki Movement MET is 2.68 | | 22.45(2.16) |
| | | | Control group | 20 | 65.97±5.66 | 70% | Maintain their original level of physical activity, do not receive any specific exercise interventions, health education | | | | 21.00(2.36) |
| | Qi,M (2019)[18] | Asia/ China | Experimental group | 16 | 70.6±6.2 | 69% | 35 minutes of moderate intensity aerobic dance (5 minutes of warm-up, 25 minutes of target heart rate dance and 5 minutes of music cooling). | 35 minutes × 3 times× 12 weeks (three months). | 60-80% maximum heart rate as the target heart rate for aerobic dance | 27.3 (1.3) | 22.6 (2.1) |
| | | | Control group | 16 | 69.1±8.1 | 75% | Routine care | | | 27.1 (1.2) | 22.9 (1.7) |
| | Zhu,Y (2018)[19] | Asia/ China | Experimental group | 29 | 70.3±6.7 | 51.7% | 5 minutes of warm-up, 25 minutes of target heart rate dance and 5 minutes of cooling | 35 minutes × 3 times× 12 weeks (three months | The target heart rate is set at 60%-80% of your maximum heart rate | | 23.2 (1.9) |
| | | | Control group | 31 | 69.0±7.3 | 67.7% | Routine care | | | | 22.9 (2.1) |
| | Lazarou, (2017)[20] | Euro-American/ Greece | Experimental group | 66 | 65.89± 10.76 | 80% | International Ballroom Dance (5 minutes of warm-up, review of previous dance lessons, 45 minutes of new material (tricks/dances), and 10 minutes of cooling time). | 60 minutes× 2 times× 40 weeks (10 months). | Dancing About 6 METs | 27.60 2.19 | 24.70 2.25 |
| | | | Control group | 33 | 67.92±9.47 | 76% | No intervention was accepted, the educational meeting was received | | | 26.88 2.1 | 23.81 3.1 |
| | Doi,T (2017)[21] | Asia/ Japan | Experimental group | 67 | 75.7±4.1 | 50.7% | The dance program is based on ballroom dances, including salsa, rumba, waltz, cha-cha, bruce, kitba and tango | 60 minutes × once × 40 weeks | Dancing About 6 METs | 26.0 (2.6) | |
| | | | Control group | 67 | 76.0±4.9 | 46.3% | Health education class | | | 25.8 (2.4) | |
| | Lam (2014)[22] | Asia/ China | Experimental group | 171 | 77.2±6.3 | 73% | 24 Styles of Tai Chi " Training. | During the introductory phase, the coaching process is 4 to 6 weeks. | The metabolic equivalent of Tai Chi exercise is 3.5-4.3 MET | 25.1±3.0 | |

For peer review only

| | | | | | | | | | | | |
|---|---|---|---|---|---|---|---|---|---|---|---|
| | | | | | | | | During the maintenance phase, the frequency of interventions was ≥ 30 minutes per day and ≥ 3 days a week. Until the 1stmonth of the 2nd month | | | |
| | | | Control group | 218 | 78.3±6.6 | 79% | Stretching and relaxation exercises | | | 24.4±2.9 | |
| Conventional aerobic exercise | Tomoto, (2021)[23] | Euro-American /USA | Experimental group | 22 | 64.8 ± 6.4 | 55% | Treadmill running | 1-10 weeks: 25-30 minutes × 3 times.<br>11-25 weeks: 30-35minutes× 3 or 4 times.26 weeks - 1 year: 30-40 minutes× 4-5 times | The intensity is 75%-85% of the maximum heart rate (HR). | 29.0 ± 1.5 | |
| | | | Control group | 30 | 66. 1 ± 6.8 | 53% | Stretching and conditioning interventions | | | 28.8 ± 1.4 | |
| | Song,D (2019)[24] | Asia/ China | Experimental group | 60 | 76.22 ±5.76 | 80% | Aerobic tread exercises | 60 minutes× 3 times × 16 weeks | For moderate-intensity workouts using the Borg Scale, the Borg score is 12-14 | | 22.03 ? 1.81 |
| | | | Control group | 60 | 75.33 ±6.78 | 70% | Health education class | | | | 22.10 ? 1.92 |
| | Tao,J (2019)[10] ② | Asia/ China | Experimental group | 17 | 64.32±2.60 | 59% | Warm up for 15 minutes, walk fast for 40 minutes, cool down for 5 minutes. | 60 minutes × 3 times × 24 weeks | The participants' heart rate was controlled at 55%-75% of the reserve heart rate | | 21.47(2.27) |
| | | | Control group | 20 | 65.97±5.6 | 70% | Maintain the original level of physical activity and do not receive any specific exercise interventions, health education | | | | 21.00(2.36) |
| | Choi,W (2018)[25] | Asia/ South Korea | Experimental group | 30 | 74.90±5.10 | 80% | 10min warm-up activity, 40min GKP exercise and 10min cooling activity. | 60 minutes× 2 times × 6 weeks | Unknown | | 21.66 (3.24 |
| | | | Control group | 30 | 74.23±4.38 | 83% | Stretching, warm-up and cooling exercises are the same as in the exercise group | | | | 20.76 (3.02) |
| | Kohanpour (2017)[26] | Asia/ Iran | Experimental group | 10 | 60-70 | 0% | 8min warm-up, (8-26)min running and cooling down for 5min | 21-39 minutes × 3 times × 12 weeks | The intensity is 75% to 85% of the maximum heart rate | 22.70±1.63 | |
| | | | Control group | 10 | 60-70 | 0% | Habitual lifestyle | | | 24.30±0.94 | |
| | Wei, X H (2014)[27] | Asia/ China | Experimental group | 30 | 66.73±5.48 | 30% | Handball training | 30 minutes × 5 times × 6 months (120 times). | The average intensity of aerobic exercise is about 60% of your maximum heart rate | 24.33(1.65) | |
| | | | Control group | 30 | 65.27±4.63 | 36.7% | Maintain the original way of life and entertainment | | | 25.00(1.29) | |

For peer review only

| | | | | | | | | | |
|---|---|---|---|---|---|---|---|---|---|
| Varela, S (2012)[11] ① | Euro-American/ Spain | Experimental group | 17 | 79.24± 10.07 | Unknown | 5 minutes of warm-up, 20 minutes of recumbent bike ride, and 5 minutes of stretching to cool down | 30 minutes × 3 times × 12 weeks | Exercise intensity is set at 40% of the participant's reserve heart rate (maximum heart rate - resting heart rate). | 19.86 ? 5.12 |
| | | Control group | 15 | 79.40 ±6.72 | | Social entertainment | | | 21.80 ? 3.23 |
| Varela, S (2012)[11] ② | Euro-American/ Spain | Experimental group | 16 | 76.44 ±11.38 | Unknown | 5 minutes of warm-up, 20 minutes of recumbent bike ride, and 5 minutes of stretching to cool down | 30 minutes × 3 times × 12 weeks | The exercise intensity is set at 60% of the participant's reserve heart rate (maximum heart rate - resting heart rate). | 20.81 ? 4.69 |
| | | Control group | 15 | 79.40 ±6.72 | | Social entertainment | | | 21.80 ? 3.23 |

For peer review only

| | Deviation risk rating | Number of studies | Numberof participants | MD | 95% CI | $I^2$ | Q-test | P |
|---|---|---|---|---|---|---|---|---|
| Multi-component exercise (MMSE) | Low | 0 | – | – | – | – | – | – |
| | Moderate | 1 | 52 | 4.60 | 2.20,7.00 | – | – | – |
| | High | 4 | 441 | 1.72 | 1.33,2.11 | 96% | <0. 00001 | <0. 00001 |
| mind-body exercise (MMSE) | Low | 0 | – | – | – | – | – | – |
| | Moderate | 2 | 383 | 1.03 | 0.43,1.64 | 0% | 0.57 | 0.0008 |
| | High | 2 | 161 | 1.61 | 0.91,2.30 | 76% | 0.04 | <0. 00001 |
| conventional aerobic exercise (MMSE) | Low | 0 | – | – | – | – | – | – |
| | Moderate | 5 | 195 | 0.51 | 0.09,0.93 | 0% | 0.46 | 0.02 |
| | High | 0 | – | – | – | – | – | – |
| Multi-component exercise(MoCA) | Low | 0 | – | – | – | – | – | – |
| | Moderate | 0 | – | – | – | – | – | – |
| | High | 1 | 84 | 5.74 | 5.02,6.46 | – | – | <0. 00001 |
| mind-body exercise ( MoCA) | Low | 1 | 58 | 1.10 | 0.06,2.14 | – | – | 0.04 |
| | Moderate | 1 | 40 | 2.45 | 0.92,3.98 | – | – | 0.002 |
| | High | 2 | 78 | 1.04 | 0.17,1.91 | 6% | 0.46 | 0.02 |
| conventional aerobic exercise(MoCA) | Low | 1 | 120 | 2.26 | 1.51,3.01 | – | – | <0. 00001 |
| | Moderate | 1 | 37 | 0.25 | -1.04,1.54 | – | – | 0.70 |
| | High | 1 | 60 | 3.67 | 2.18,5.16 | – | – | <0. 00001 |

For peer review only

The subgroup analysis of Multi-component exercise (MMSE)

| Subgroups | | Number of studies | Numberof participants | MD | 95% CI | $I^2$ | Q-test | $P^g$ |
|---|---|---|---|---|---|---|---|---|
| Sample size [a] | Small | 2 | 71 | 1.69* | 0. 30，3. 08 | 88% | ＜0.01 | =0. 88 |
| | Large | 3 | 422 | 1.80** | 1. 40，2. 20 | 97% | ＜0. 01 | |
| region | Asia | 3 | 422 | 1.80** | 1. 40，2. 20 | 97% | ＜0. 01 | =0. 88 |
| | Europe | 2 | 71 | 1.69* | 0. 30，3. 08 | 88% | ＜0. 01 | |
| Exercise intensity [b] | low | 1 | 84 | 2.37** | 1. 59，3. 15 | — | — | =0.01 |
| | Medium to high | 3 | 131 | 3.68** | 3. 00，4. 36 | 88% | ＜0. 01 | |
| Duration of intervention [c] | short | 1 | 84 | 2.37** | 1. 59，3. 15 | — | — | ＜0. 01 |
| | middle | 3 | 349 | 0.33 | -0. 20，0. 87 | 84% | ＜0. 01 | |
| | long | 1 | 60 | 4.30** | 3. 53，5. 08 | — | — | |
| Frequency of intervention [d] | middle | 3 | 349 | 0.33 | -0. 20，0. 87 | 84% | ＜0. 01 | ＜0. 01 |
| | high | 2 | 144 | 3.34** | 2. 79，3. 88 | 92% | ＜0. 01 | |
| Total intervention time [e] | middle | 4 | 409 | 1.61** | 1. 17，2. 05 | 96% | ＜0. 01 | =0.09 |
| | long | 1 | 84 | 2.37** | 1. 59，3. 15 | — | — | |
| Deviation risk rating [f] | middle | 1 | 52 | 4.60** | 2.20，7.00 | — | — | ＜0. 01 |
| | high | 4 | 441 | 1.72** | 1.33， 2.11 | 96% | ＜0. 01 | |

[a]＜60=Small sample size；≥60=Large sample size，[b]Maximum heart rate <60% or Borg scale (10th grade) <5 or Borg scale (20th grade) < 12 or metabolic equivalent (MET) <3 or reserve heart rate <59% =Low strength；Maximum heart rate ≥ 60% or Borg scale (10th grade) ≥5 or Borg scale (20th grade) ≥ 12 or metabolic equivalent (MET) ≥3 or reserve heart rate ≥59% = medium to high intensity，[c]≤30 minutes = short；＞30 且≤60 minutes = middle； >60

minutes =long [d] 1 time/week = low; 2-3 times / week = middle； 4 times and more /week =high [e]＜3 months (12 weeks) = short； ≥ 3 months and < 6 months (12-23 weeks) = middle； ≥6 months (24 weeks) =long [f]All criteria are rated as low risk= Low risk of bias；Some standards are rated as unclear and not rated as high risk = medium deviation risk; As long as a standard is rated as high risk = high risk of deviation, [g]Differences between groups

[*] P＜0.05

[**] P＜0.01

For peer review only

## Quality of the evidence(GRADE)

| Quality assessment | | | | | | | No of patients | | Effect | | Quality | Importance |
|---|---|---|---|---|---|---|---|---|---|---|---|---|
| No of studies | Design | Risk of bias | Inconsistency | Indirectness | Imprecision | Other considerations | Aerobic exercise group | Control group | Relative (95% CI) | Absolute | | |
| **Subgroup analysis of different Exercise Types (MMSE) - multi-component aerobic exercise VS controls (Better indicated by lower values)** | | | | | | | | | | | | |
| 1 | randomised trials | very serious[1] | very serious[2] | no serious indirectness | no serious imprecision | none | 257 | 236 | - | MD 1.79 higher (1.41 to 2.17 higher) | ⊕OOO VERY LOW | |
| **Subgroup analysis of different Exercise Types (MMSE) - mind-body exercise VS controls (Better indicated by lower values)** | | | | | | | | | | | | |
| 2 | randomised trials | serious[3] | no serious inconsistency | no serious indirectness | no serious imprecision | none | 233 | 311 | - | MD 1.28 higher (0.83 to 1.74 higher) | ⊕⊕⊕O MODERATE | |
| **Subgroup analysis of different Exercise Types (MMSE) - Conventional aerobic exercise VS controls (Better indicated by lower values)** | | | | | | | | | | | | |
| 3 | randomised trials | serious[3] | no serious inconsistency | no serious indirectness | serious[4] | none | 95 | 100 | - | MD 0.51 higher (0.09 to 0.93 higher) | ⊕⊕OO LOW | |

[1] Downgraded two levels due to most studies are high risk.

[2] Downgraded two levels due to $I^2 > 75\%$.

[3] Downgraded one level due to most studies are of low and moderate risk.

[4] Downgraded one level due to the samle size is small.

| Quality assessment | | | | | | | No of patients | | Effect | | Quality | Importance |
|---|---|---|---|---|---|---|---|---|---|---|---|---|
| No of studies | Design | Risk of bias | Inconsistency | Indirectness | Imprecision | Other considerations | Aerobic exercise group | Control group | Relative (95% CI) | Absolute | | |
| **Subgroup analysis of different Exercise Types (MoCA)- multi-component aerobic exercise VS controls (Better indicated by lower values)** | | | | | | | | | | | | |
| 1 | randomised trials | very serious[1] | no serious inconsistency | no serious indirectness | very serious[2] | none | 42 | 42 | - | MD 5.74 higher (5.02 to 6.46 higher) | ⊕OOO VERY LOW | |
| **Subgroup analysis of different Exercise Types (MoCA) - mind-body exercise VS controls (Better indicated by lower values)** | | | | | | | | | | | | |
| 2 | randomised trials | serious[3] | no serious inconsistency | no serious indirectness | serious[4] | none | 85 | 91 | - | MD 1.29 higher (0.67 to 1.9 higher) | ⊕⊕OO LOW | |
| **Subgroup analysis of different Exercise Types (MoCA) - Conventional aerobic exercise VS controls (Better indicated by lower values)** | | | | | | | | | | | | |
| 3 | randomised trials | serious[3] | very serious[5] | no serious indirectness | serious[4] | none | 107 | 110 | - | MD 2.06 higher (1.46 to 2.65 higher) | ⊕OOO VERY LOW | |

[1] Downgraded two levels due to most studies are high risk.

[2] Downgraded two levels due to the sample size is too small.

[3] Downgraded one level due to most studies are of low and moderate risk.

[4] Downgraded one level due to the sample size is small.

[5] Downgraded two levels due to I² > 75%.

For peer review only

| Total sensitivity analysis（MMSE） | | | Total sensitivity analysis(MoCA) | | |
|---|---|---|---|---|---|
| Study omitted | Mean difference | p-value | Study omitted | Mean difference | p-value |
| Bademli K 2019 | 0.91 [0.65,1.16] | <0.00001 | Choi W 2018 | 2.90[2.53,3.26] | <0.00001 |
| de Oliveira2019 | 1.25 [1.01,1.50] | <0.00001 | Lazarou 2017 | 2.74[2.37,3.10] | <0.00001 |
| Doi T 2017 | 1.26 [1.01,1.51] | <0.00001 | Li F 2022 | 3.14[2.77,3.52] | <0.00001 |
| Kohanpour 2017 | 1.30 [1.05,1.55] | <0.00001 | Li L 2021 | 2.03[1.62,2.44] | <0.00001 |
| Lam LC 2014 | 1.24[0.98,1.49] | <0.00001 | Qi M 2019 | 3.09[2.72,3.46] | <0.00001 |
| Lam LCW 2015 | 1.47[1.21,1.74] | <0.00001 | Song D 2019 | 3.14[2.73,3.54] | <0.00001 |
| Langoni 2019 | 1.20[0.96,1.44] | <0.00001 | Tao J 2019① | 2.97[2.60,3.33] | <0.00001 |
| Lazarou 2017 | 1.16[0.92,1.41] | <0.00001 | Tao J 2019② | 3.16[2.79,3.53] | <0.00001 |
| Li L 2021 | 1.11[0.86,1.37] | <0.00001 | Zhu Y 2018 | 3.18[2.80,3.56] | <0.00001 |
| Qi M 2019 | 1.25[1.01,1.50] | <0.00001 | Choi W 2018 | 2.90[2.53,3.26] | <0.00001 |
| Tomoto T 2021 | 1.35[1.10,1.60] | <0.00001 | | | |
| Varela S 2012① | 1.23[0.99,1.47] | <0.00001 | | | |
| Varela S 2012② | 1.23[0.99,1.47] | <0.00001 | | | |
| Wei XH 2014 | 1.31[1.04,1.57] | <0.00001 | | | |
| The sensitivity analysis of Multi-component motion (MMSE) | | | | | |
| Study omitted | Mean difference | p-value | | | |
| Bademli K 2019 | 0.99[0.55,1.43] | <0.0001 | | | |
| by Oliveira2019 | 1.88[1.48,2.27] | <0.00001 | | | |
| Lam LCW 2015 | 3.11[2.60,3.63] | <0.00001 | | | |
| 2019 Langoni | 1.72[1.33,2.11] | <0.00001 | | | |
| Li L 2021 | 1.61[1.17,2.05] | <0.00001 | | | |
| The sensitivity analysis of mind-body exercise（MMSE） | | | The sensitivity analysis of mind-body exercise (MoCA) | | |
| Study omitted | Mean difference | p-value | Study omitted | Mean difference | p-value |
| Doi T 2017 | 1.44 [0.91,1.97] | <0.00001 | Li F 2022 | 1.29(0.55,2.03) | 0. 0007 |
| Lam LC 2014 | 1.31 [0.77,1.86] | <0.00001 | Qi M 2019 | 1.43(0.76,2.11) | <0.0001 |
| Lazarou 2017 | 1.00 [0.48,1.51] | <0.00001 | Tao J 2019① | 1.06(0.40,1.73) | 0. 002 |
| Qi M 2019 | 1.39[0.87,1.90] | <0.00001 | Zhu Y 2018 | 1.38(0.63,2.14) | 0. 0003 |
| The sensitivity analysis of conventional aerobic exercise (MMSE） | | | The sensitivity analysis of conventional aerobic exercise (MoCA). | | |
| Study omitted | Mean difference | p-value | Study omitted | Mean difference | p-value |
| Kohanpour 2017 | 0.59(0.12,1.06) | 0. 01 | Choi W 2018 | 1.75(1.10,2.40) | <0.0001 |
| Tomoto T 2021 | 0.70(0.21,1.19) | 0.005 | Song D 2019 | 1.71(0.74,2.69) | <0.00001 |
| Varela S 2012① | 0.51(0.08,0.93) | 0. 02 | Tao J 2019② | 2.55(1.87,3.22) | <0.00001 |
| Varela S 2012② | 0.50(0.07,0.92) | 0. 02 | | | |
| Wei XH 2014 | 0.51(0.09,0.93) | 0. 65 | | | |

For peer review only

# PRISMA 2020 Checklist

| Section and Topic | Item # | Checklist item | Location where item is reported |
|---|---|---|---|
| **TITLE** | | | |
| Title | 1 | Identify the report as a systematic review. | Page 1 |
| **ABSTRACT** | | | |
| Abstract | 2 | See the PRISMA 2020 for Abstracts checklist. | Page 2 |
| **INTRODUCTION** | | | |
| Rationale | 3 | Describe the rationale for the review in the context of existing knowledge. | Page 2 |
| Objectives | 4 | Provide an explicit statement of the objective(s) or question(s) the review addresses. | Page 1 |
| **METHODS** | | | |
| Eligibility criteria | 5 | Specify the inclusion and exclusion criteria for the review and how studies were grouped for the syntheses. | Page 2 |
| Information sources | 6 | Specify all databases, registers, websites, organisations, reference lists and other sources searched or consulted to identify studies. Specify the date when each source was last searched or consulted. | Page 2 and figure |
| Search strategy | 7 | Present the full search strategies for all databases, registers and websites, including any filters and limits used. | supplementary statement |
| Selection process | 8 | Specify the methods used to decide whether a study met the inclusion criteria of the review, including how many reviewers screened each record and each report retrieved, whether they worked independently, and if applicable, details of automation tools used in the process. | Page 2 |
| Data collection process | 9 | Specify the methods used to collect data from reports, including how many reviewers collected data from each report, whether they worked independently, any processes for obtaining or confirming data from study investigators, and if applicable, details of automation tools used in the process. | Page 3 |
| Data items | 10a | List and define all outcomes for which data were sought. Specify whether all results that were compatible with each outcome domain in each study were sought (e.g. for all measures, time points, analyses), and if not, the methods used to decide which results to collect. | Page 3 |
| | 10b | List and define all other variables for which data were sought (e.g. participant and intervention characteristics, funding sources). Describe any assumptions made about any missing or unclear information. | Page 2 |
| Study risk of bias assessment | 11 | Specify the methods used to assess risk of bias in the included studies, including details of the tool(s) used, how many reviewers assessed each study and whether they worked independently, and if applicable, details of automation tools used in the process. | Page 3 |
| Effect measures | 12 | Specify for each outcome the effect measure(s) (e.g. risk ratio, mean difference) used in the synthesis or presentation of results. | Page 3 |
| Synthesis methods | 13a | Describe the processes used to decide which studies were eligible for each synthesis (e.g. tabulating the study intervention characteristics and comparing against the planned groups for each synthesis (item #5)). | Page 2 |
| | 13b | Describe any methods required to prepare the data for presentation or synthesis, such as handling of missing summary statistics, or data conversions. | Page 3 |
| | 13c | Describe any methods used to tabulate or visually display results of individual studies and syntheses. | Page 3 |
| | 13d | Describe any methods used to synthesize results and provide a rationale for the choice(s). If meta-analysis was performed, describe the model(s), method(s) to identify the presence and extent of statistical heterogeneity, and software package(s) used. | Page 3 |
| | 13e | Describe any methods used to explore possible causes of heterogeneity among study results (e.g. subgroup analysis, meta-regression). | Page 3 |
| | 13f | Describe any sensitivity analyses conducted to assess robustness of the synthesized results. | Page 3 |
| Reporting bias assessment | 14 | Describe any methods used to assess risk of bias due to missing results in a synthesis (arising from reporting biases). | -( Does not meet the number of |

For peer review only - http://bmjopen.bmj.com/site/about/guidelines.xhtml

# PRISMA 2020 Checklist

| Section and Topic | Item # | Checklist item | Location where item is reported |
|---|---|---|---|
| | | | studies required for funnel plots) |
| Certainty assessment | 15 | Describe any methods used to assess certainty (or confidence) in the body of evidence for an outcome. | Page 8 |
| **RESULTS** | | | |
| Study selection | 16a | Describe the results of the search and selection process, from the number of records identified in the search to the number of studies included in the review, ideally using a flow diagram. | Figure 1 |
| | 16b | Cite studies that might appear to meet the inclusion criteria, but which were excluded, and explain why they were excluded. | Figure 1 |
| Study characteristics | 17 | Cite each included study and present its characteristics. | Table 2 |
| Risk of bias in studies | 18 | Present assessments of risk of bias for each included study. | Figure 2 |
| Results of individual studies | 19 | For all outcomes, present, for each study: (a) summary statistics for each group (where appropriate) and (b) an effect estimate and its precision (e.g. confidence/credible interval), ideally using structured tables or plots. | All figure |
| Results of syntheses | 20a | For each synthesis, briefly summarise the characteristics and risk of bias among contributing studies. | Page 8 |
| | 20b | Present results of all statistical syntheses conducted. If meta-analysis was done, present for each the summary estimate and its precision (e.g. confidence/credible interval) and measures of statistical heterogeneity. If comparing groups, describe the direction of the effect. | Figure 3-6<br>Page 8-10 |
| | 20c | Present results of all investigations of possible causes of heterogeneity among study results. | Page 8<br>Figure 2 |
| | 20d | Present results of all sensitivity analyses conducted to assess the robustness of the synthesized results. | Figure 11 |
| Reporting biases | 21 | Present assessments of risk of bias due to missing results (arising from reporting biases) for each synthesis assessed. | - |
| Certainty of evidence | 22 | Present assessments of certainty (or confidence) in the body of evidence for each outcome assessed. | Table 4 |
| **DISCUSSION** | | | |
| Discussion | 23a | Provide a general interpretation of the results in the context of other evidence. | Page 10 |
| | 23b | Discuss any limitations of the evidence included in the review. | Page 11 |
| | 23c | Discuss any limitations of the review processes used. | Page 11 |
| | 23d | Discuss implications of the results for practice, policy, and future research. | Page 11 |
| **OTHER INFORMATION** | | | |
| Registration and protocol | 24a | Provide registration information for the review, including register name and registration number, or state that the review was not registered. | Page 2 |
| | 24b | Indicate where the review protocol can be accessed, or state that a protocol was not prepared. | - |
| | 24c | Describe and explain any amendments to information provided at registration or in the protocol. | -( Not modified) |
| Support | 25 | Describe sources of financial or non-financial support for the review, and the role of the funders or sponsors in the review. | Page 15 |
| Competing | 26 | Declare any competing interests of review authors. | - |

For peer review only - http://bmjopen.bmj.com/site/about/guidelines.xhtml

# PRISMA 2020 Checklist

| Section and Topic | Item # | Checklist item | Location where item is reported |
|---|---|---|---|
| interests | | | |
| Availability of data, code and other materials | 27 | Report which of the following are publicly available and where they can be found: template data collection forms; data extracted from included studies; data used for all analyses; analytic code; any other materials used in the review. | Page 2 and Page 3 |

*From:* Page MJ, McKenzie JE, Bossuyt PM, Boutron I, Hoffmann TC, Mulrow CD, et al. The PRISMA 2020 statement: an updated guideline for reporting systematic reviews. BMJ 2021;372:n71. doi: 10.1136/bmj.n71

For more information, visit: http://www.prisma-statement.org/

For peer review only
